# A Luminescence-Based Screening Platform for Lanthanide-Binding Peptides and Proteins

**DOI:** 10.1021/acschembio.5c00670

**Published:** 2025-11-17

**Authors:** Robert Klassen, Anna Heider, Hannah Kugler, Michael Groll, Cathleen Zeymer

**Affiliations:** Center for Functional Protein Assemblies & Department of Bioscience, TUM School of Natural Sciences, 9184 Technical University of Munich (TUM), Garching 85748, Germany

## Abstract

The specific incorporation of lanthanide ions is a promising strategy to equip biomolecules with a new function. Their long-lived luminescence, strong anomalous X-ray scattering, paramagnetism, Lewis acidity, and photoredox activity are attractive features for protein-based probes, materials, and catalysts. However, natural lanthanide-binding proteins are rare, and de novo design is often complicated by unspecific binding to negatively charged patches on protein surfaces. We thus aimed to develop an efficient workflow to screen libraries of protein scaffolds for their ability to coordinate lanthanides. Here, we introduce a microtiter plate-based assay, which employs commercial filter plates and a dual readout based on sensitized Tb^3+^ luminescence. We first benchmarked our procedure using control proteins with and without lanthanide-binding sites, demonstrating that site-specific coordination and surface binding can be distinguished. The stringency of this protocol also allowed screening for small lanthanide-binding peptides in the presence of a large expression tag. We then designed a de novo scaffold library derived from a helical bundle protein and applied our screening platform. We could identify lanthanide-binding variants with nanomolar affinity, distinct lanthanide specificity, and increased thermostability in response to metal binding. Our approach will support the discovery and evolution of lanthanide-binding peptides and proteins for various applications in vitro and in living cells.

## Graphical abstract

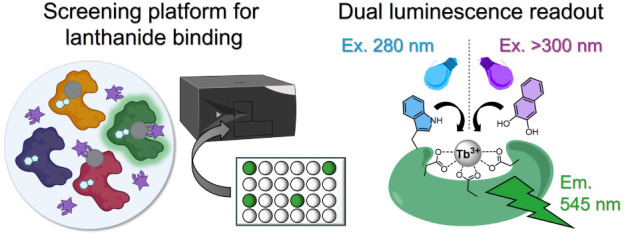

## Introduction

Lanthanides (Ln) comprise the 4*f* block elements of the periodic table. Their trivalent cations are characterized by strong Lewis acidity, oxophilicity, and a preference for high coordination numbers.^1,2^ Ligand binding to Ln^3+^ ions is mediated through nondirective Coulomb attraction, allowing the construction of a chiral coordination environment. Such complexes are powerful Lewis acid catalysts, for a broad panel of chemical transformations.^3,4^ Moreover, cerium, europium, and samarium compounds have been applied in photoredox chemistry proceeding via single electron transfer.^5–10^ To enhance the catalytic utility of lanthanides, their incorporation into proteins is an attractive strategy, as recently demonstrated by developing an artificial cerium-dependent photoenzyme.^11^

Lanthanide-based materials find applications in displays, lasers, magnets, and batteries and are thus in high demand.^12–15^ To further exploit their distinct optical and magnetic properties for these various technologies, more sustainable and geopolitically independent alternatives to the classic mining of rare earth ores are urgently needed. Triggered by the discovery of lanthanide-utilizing bacteria and the in-depth characterization of the proteins they evolved to specifically bind these metals,^16–18^ bioinspired lanthanide recycling and separation approaches are currently in development.^19–23^

Lanthanide coordination in proteins is mediated by carboxylates (Asp/Glu side chains) and amide carbonyl groups (Asn/Gln side chains and protein backbone).^17^ In contrast to structurally related calcium binding sites, an additional carboxylate typically increases the specificity for the trivalent lanthanide ions. Alcohol dehydrogenases (e.g., XoxF from *M. extorquens* ^24^ or PedH from *P. putida* ^25^) remain the only natural lanthanide-dependent enzyme class known to date. These enzymes preferably accommodate the abundant light Ln^3+^ ions in their active sites to activate a pyrroloquinoline quinone (PQQ) redox cofactor for alcohol oxidation.^18^ Furthermore, noncatalytic binding proteins with high affinity and selectivity for lanthanides, such as lanmodulin (LanM), have been identified in the respective bacteria.^20,26–28^ A valuable alternative source of lanthanide-coordinating proteins is their de novo design. Due to recent AI-driven developments in the field, it now becomes feasible to tailor protein properties for desired applications.^29–32^ However, experimental testing and optimization requires a robust and efficient screening assay for lanthanide binding, which we set out to develop in this work.

First reports on engineered lanthanide-binding proteins involved the grafting of isolated EF hand motifs (12–15 amino acid long Ca^2+^ binding loops) onto natural proteins to study metal binding.^33^ Linking DNA-binding helix-turn-helix domains with an EF hand sequence resulted in small artificial nucleases, which utilized the hydrolytic activity of the bound lanthanide to cleave phosphate ester bonds.^34,35^ An elegant protocol to develop high-affinity lanthanide-binding tags (LBTs) from EF hand peptides applied a combinatorial split-and-pool method for synthetic peptide library generation and tryptophan-sensitized Tb^3+^ luminescence screening in a Tb^3+^-doped agarose matrix.^36,37^ The resulting LBT sequences can be genetically encoded to harness lanthanide luminescence, anomalous X-ray scattering, and paramagnetism when studying protein structure, function, and dynamics.^38^ With the advent of computational protein design, the precise installation of metal binding sites into de novo protein scaffolds has drastically improved.^39–43^ Examples of de novo designs include coiled coils^44,45^ and a TIM-ferredoxin dimer scaffold,^46^ both offering tailor-made coordination spheres for their desired lanthanide-dependent function: gadolinium-based MRI contrast enhancement^44,47^ and cerium-based photoenzymatic activity,^11^ respectively.

Here, we report a luminescence-based assay to efficiently identify lanthanide-coordinating proteins and peptides in 96-well plate format after recombinant expression in *E. coli* rather than chemical synthesis of library members. We successfully applied our screening platform to select nanomolar binders from a de novo scaffold library. Our work thus provides a valuable tool for the development of designer proteins for future lanthanide-based biotechnological applications.

## Results and Discussion

### Assay Design

To screen for lanthanide binding to proteins, we envisaged a dual luminescence readout utilizing a representative Ln^3+^ ion together with an internal and an external sensitizer (Figure 1A). The sensitizers serve as an antenna to enhance the lanthanide emission via energy transfer. The internal antenna must be covalently linked to the protein and positioned in close proximity to the intended lanthanide binding site, while the external antenna is a freely diffusing small molecule that can coordinate the Ln^3+^ ion. By choosing antennas with different excitation wavelengths, we intended to distinguish four possible binding scenarios: i) Both channels show a strong luminescence signal, indicating specific lanthanide binding inside the protein and an accessible binding pocket. ii) Only the internal antenna channel shows a signal, which implies that the bound lanthanide is not accessible for the external antenna molecule. iii) Only the external antenna channel shows a signal, suggesting unspecific lanthanide binding to the protein. iv) Both channels show only background signal, indicating no significant lanthanide binding.

**Figure 1:**
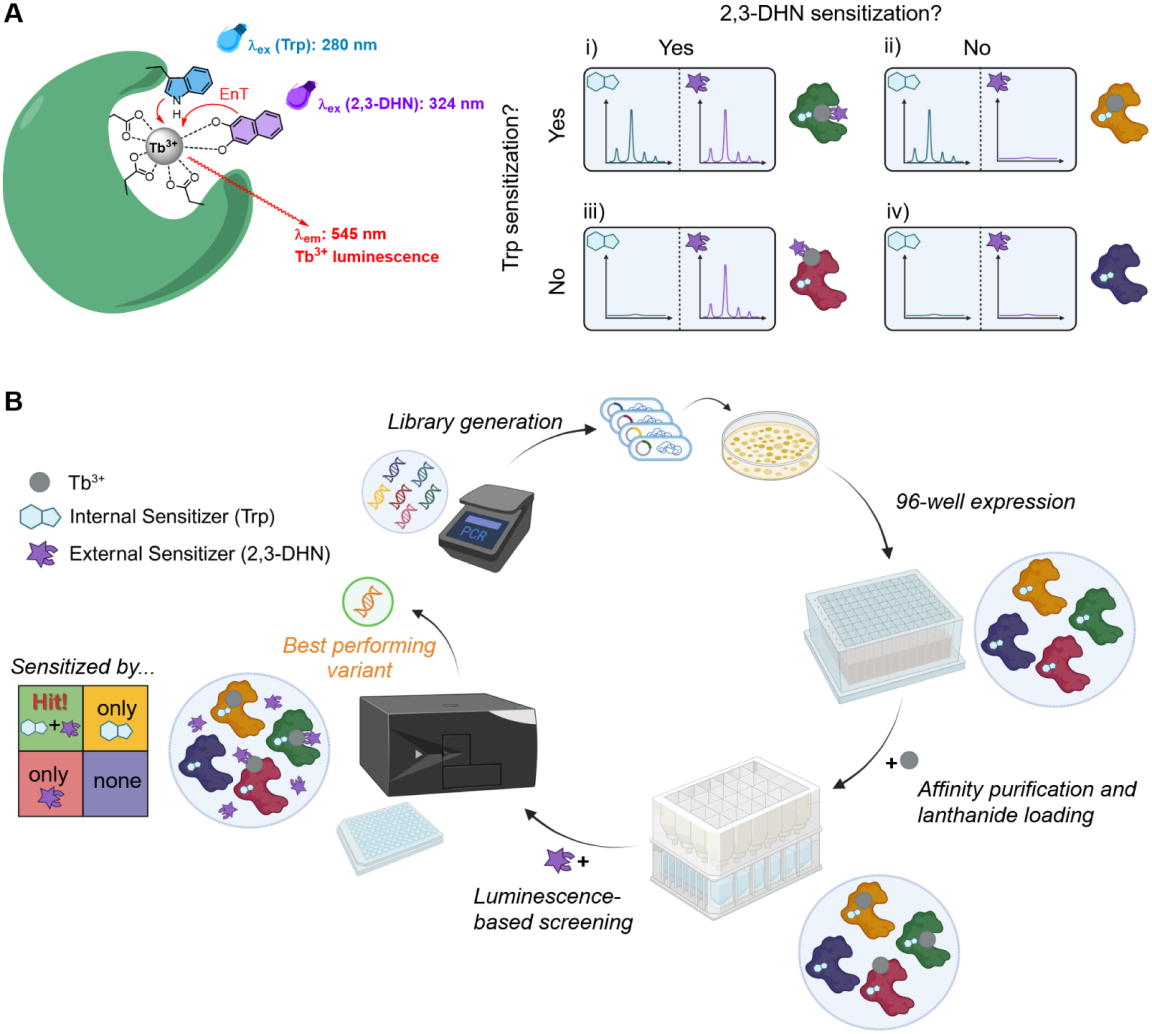
Design and overall workflow of the lanthanide binding assay. (A) The dual luminescence readout detects the Tb^3+^ emission sensitized either by Trp (internal antenna) or 2,3-DHN (external antenna). (B) General procedure to screen recombinantly expressed protein and peptide libraries for lanthanide binding. EnT—Energy transfer. Parts of this figure were designed using BioRender (panel A: https://BioRender.com/ocmmitg, panel B: https://BioRender.com/u3d9t2y).

The lanthanide ion of choice in biological assays is Tb^3+^, as its luminescence can be sensitized by tryptophan (Trp) upon excitation at 280 nm.^48,49^ We could thus conveniently use a natural amino acid side chain as the internal antenna in our assay. As a matching external antenna we selected 2,3-dihydroxynaphthalene (2,3-DHN), which was previously utilized as a sensitizer in Tb^3+^-based assays and can be excited at wavelengths >300 nm,^50,51^ thus minimizing the overlap of the sensitized luminescence channels.

Lanthanides are known to interact unspecifically with many cellular components,^52–55^ which could interfere with our assay. Most prominently, nucleotides and nucleic acids could sequester the added lanthanide ions,^56^ which may severely impair our readout. As screening in cell lysate was therefore not feasible, we included a protein purification step into our microtiter plate-based workflow. To that end, we used a commercial filter plate with an affinity resin, which enabled sequential purification, on-resin Tb^3+^ loading, and washing of the His_6_-tagged target proteins prior to elution. Metal affinity resins for Ni^2+^ complexation, such as Ni-NTA, also display high affinity for lanthanide ions.^57^ To avoid metal displacement and thus reduced binding capacity as well as an increased concentration of free Tb^3+^ in the elution fraction, we applied a plate regeneration procedure after each screening round. For the dual luminescence detection, the microtiter plate reader was used in time-resolved fluorescence (TRF) mode to increase the signal-to-noise ratio. The overall workflow is shown in Figure 1B.

While the four binding scenarios defined in Figure 1A represent idealized cases, real proteins may also fall in between these categories. Their absolute luminescence values will depend on the specific distances of Trp side chain or 2,3-DHN to the bound Tb^3+^ ions. Trp residues can also be located close to the protein surface, where Tb^3+^ may bind unspecifically, causing different levels of background signal. These factors prevented a statistically sound definition of threshold values. However, as demonstrated below, a qualitative categorization was feasible.

### Assay Validation

To confirm the suitability of on-resin Tb^3+^ loading and dual luminescence detection, we tested the assay with a set of control proteins. As a positive control, we selected the alcohol dehydrogenase PedH from *P. putida* KT2440, which binds a catalytically essential Ln^3+^ ion adjacent to its redox cofactor PQQ.^25^ The binding site possesses two Trp residues, W285 and W263, which served as internal antennas (Figure S1A). Recombinant expression in *E. coli*, which cannot biosynthesize PQQ, yields the *apo* form of PedH that still exhibits nanomolar Tb^3+^ affinity even in the absence of PQQ (Figure S1C).^11^ We hypothesized that the external antenna 2,3-DHN may occupy the empty PQQ binding pocket, which would lead to an enhanced luminescence intensity. Here, we used a previously engineered double mutant (PedH^F412 V/W561A^, subsequently referred to as “PedH”) with an enlarged substrate entry tunnel.^58^ Replacing the lanthanide-coordinating aspartates with asparagine (D323N and D325N) yielded the loss-of-function variant PedH_2xMut, which shows significantly impaired Tb^3+^ binding and was thus used as a negative control (Figure S1B/C). Furthermore, we used purified PedH to assess the spectral characteristics of our dual luminescence readout, confirming that the presence of both sensitizers does not cause significant crosstalk. (Figure S2).

We sought to evaluate if a maltose-binding protein (MBP) expression tag was compatible with our screening approach. MBP is frequently employed to improve solubility and generate uniformly high expression levels.^59^ However, it may also cause background signals due to unspecific lanthanide binding on its surface. We thus created a control protein consisting of His_6_-tagged MBP and superfolder green fluorescent protein (*sf*GFP),^60^ *N*-terminally linked via a TEV protease cleavage site. For comparison, we also included only His_6_-tagged variants of *sf*GFP, with and without TEV protease cleavage site.

Another control protein was lanmodulin (LanM), a small natural Ln^3+^ binder with picomolar affinity.^26^ It possesses EF hand-like loops that coordinatively saturate the bound Ln^3+^ ion. We thus hypothesized that the external antenna was unable to efficiently access the binding site. As wild-type LanM lacks a suitably positioned Trp residue for sensitization, we introduced mutation T41W in the EF1 loop (Figure S3, subsequently referred to as LanM_Trp), which had been reported previously.^61^ To unify protein yields, we used also LanM variants as MBP fusion constructs.

Lastly, we chose the de novo protein scaffold MID1 as a potential starting point for implementing a lanthanide binding site. We worked with variant MID1sc9, a previously evolved Zn^2+^-dependent esterase,^62^ to evaluate the extent of background signal originating from this scaffold. All our control proteins possess surface-exposed glutamate and aspartate residues as well as at least one tryptophan (Figure S3).

We started by expressing the control proteins in *E. coli* using shake flask cultures to prepare uniform and highly concentrated cell-free lysates, which were then applied on a commercial 96-well HIS Select filter plate for purification, Tb^3+^ loading, and detection procedure as outlined above (see also Figure S4). Protein concentrations in the eluted fractions ranged from 20 to 60 μM (Figure 2A). The characteristic Tb^3+^ luminescence spectrum with emission bands between 450 and 650 nm, recorded upon excitation of the sensitizers at 280 and 324 nm, respectively, confirmed the presence of protein-bound Tb^3+^ in the eluted fractions (Figure 2B). PedH showed sensitized luminescence values 10 to 100-fold higher compared to the PedH_2xMut knockout variant, depending on the excitation channel. The strong Trp-sensitized luminescence for PedH is promoted by the proximity of two Trp residues lining the Ln^3+^ binding site. The background signal detected for PedH_2xMut likely stems from surface-bound Tb^3+^ ions in proximity to one of the scaffold’s other Trp residues (Figure S3). The different ratios of Trp-sensitized and 2,3-DHN-sensitized signals for wild-type and mutant demonstrate that specific and unspecific Tb^3+^ binding can be qualitatively differentiated with the antenna duo. We next expressed PedH and PedH_2xMut in a repetitive pattern in a deep-well plate to simulate the screening scenario more closely. This experiment also showed a similarly clear distinction between both variants using the two channels (Figure 2C).

**Figure 2:**
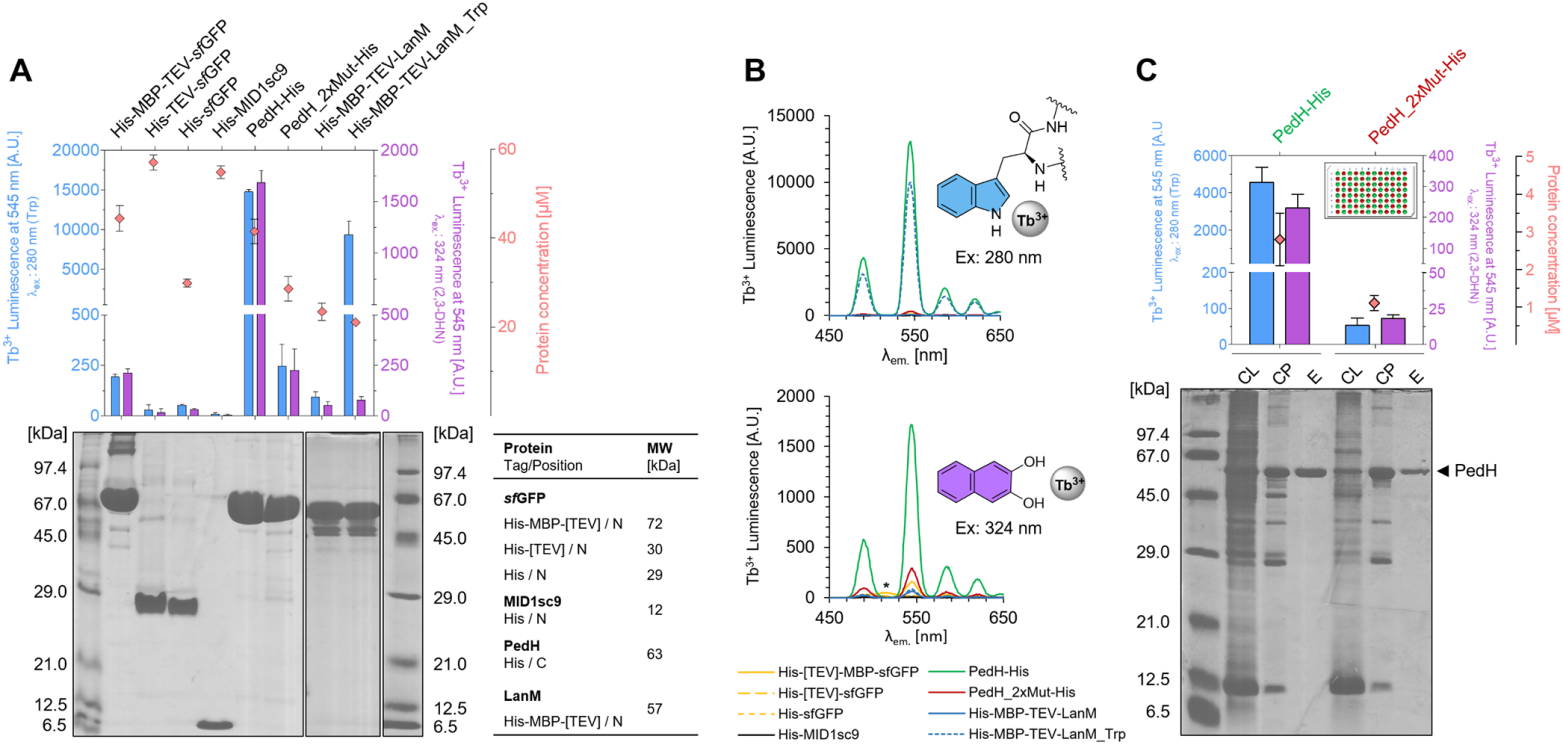
Assay validation using control proteins for on-resin Tb^3+^ loading and detection. (A) Tb^3+^ luminescence upon excitation at 280 nm (Trp-sensitized, blue bars) and 324 nm (2,3-DHN-sensitized, purple bars). Protein concentrations of the elution fractions are indicated as red diamonds. All values are averaged from eight replicates, with standard deviations shown as error bars. The SDS-PAGE gel underneath shows the elution fractions of the respective proteins, with the corresponding molecular weight, type, and position of tags listed in the table. (B) Exemplary luminescence spectra showing the characteristic Tb^3+^ emission. The responsible sensitizers are shown as structures (Trp—blue, top; 2,3-DHN—purple, bottom). The asterisk indicates a small fluorescence peak of *sf*GFP likely originating from luminescence resonance energy transfer (LRET).^63^ (C) Tb^3+^ luminescence of PedH and PedH_2xMut, which were expressed and purified in 96-well format (plate layout is shown as inset). The values are averages from 48 wells, with standard deviations shown as error bars. The SDS-PAGE gel underneath shows samples of the cell lysate (CL), cell pellet (CP), and elution (E) of the respective protein.

The MBP tag turned out to be suitable for our approach. Its background luminescence signal was comparable to that of PedH_2xMut, indicating some degree of unspecific Tb^3+^ binding on the protein surface, while *sf*GFP alone gave a neglectable signal (Figure 2A/B). The MBP fusion of LanM_Trp gave the expected strong luminescence signal for excitation at 280 nm, but remained at background level for the 2,3-DHN-sensitized signal. This suggests that the bound Tb^3+^ ion is indeed not accessible for coordination by the external antenna in the case of lanmodulin. MID1sc9 showed the lowest background luminescence, indicating no coordination sites for Tb^3+^, which confirmed its choice as a suitable starting point for later library generation and screening.

### Screening of Short Peptide Sequences

We next asked if our screening platform would also be applicable to identify short peptidic lanthanide binders. We selected the previously reported class of Trp zipper peptides as our test case. Trp zippers (TrpZip) are short peptide sequences (12–16 aa), which fold into an antiparallel β-hairpin (Figure S6A) with strong intrinsic rigidity and thermostability.^64^ The fold is stabilized by four Trp residues, which would readily serve as efficient internal antennas for our Tb^3+^ luminescence readout. Previous work showed the conversion of a TrpZip sequence into a transition metal binder, Tz2H3, with nM affinities toward Cu^2+^, Ni^2+^ and Zn^2+^. This was achieved by replacing residues T3, T10 and K12 by metal-coordinating histidines.^65^ Inspired by this design, we targeted the same positions to introduce Asp and Glu, respectively, for dative lanthanide coordination. The two peptides were termed LanPep1 and LanPep2 (Figure 3A and Figure S6B).

**Figure 3:**
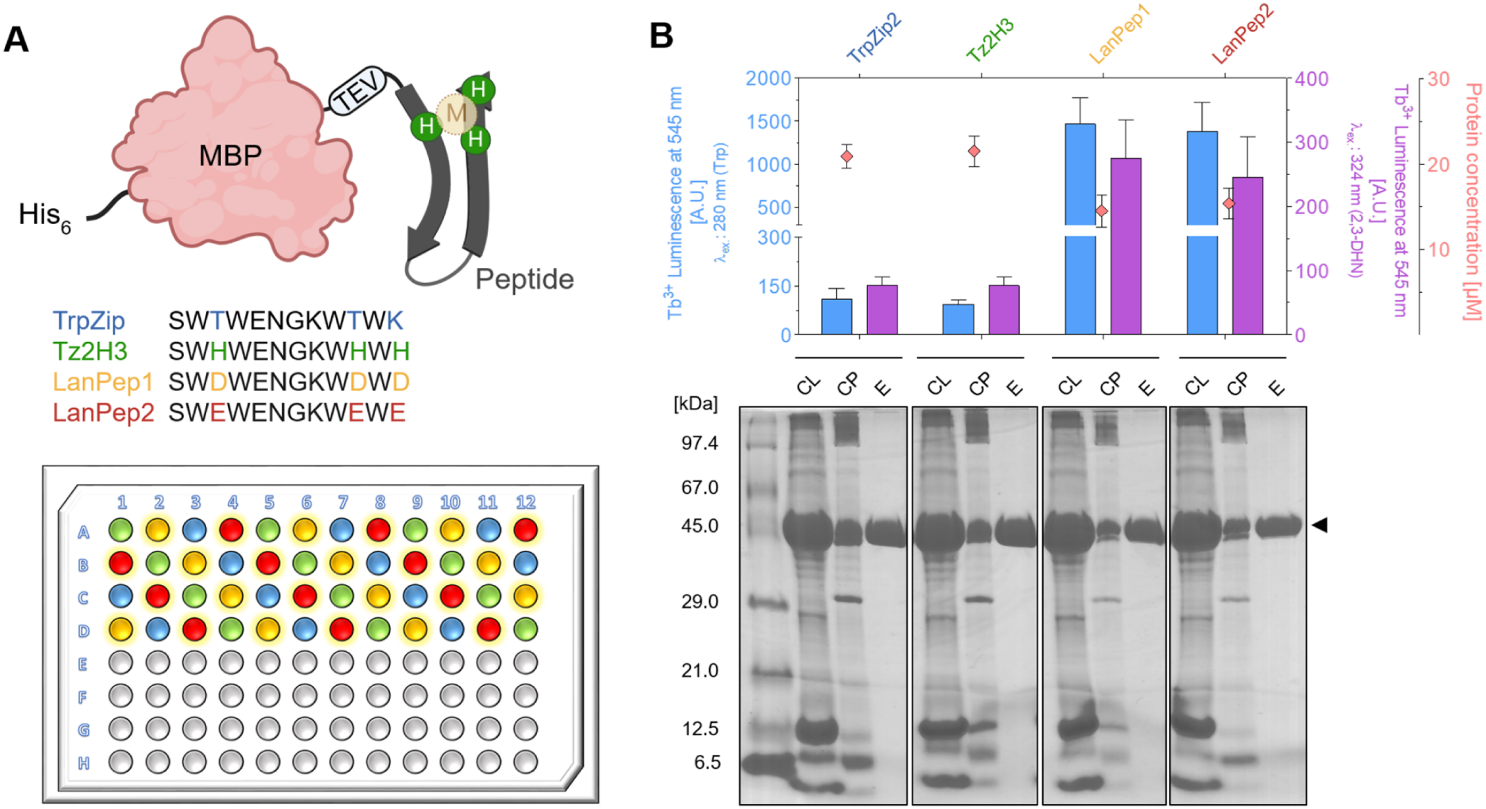
Screening MBP-peptide fusions for Tb^3+^ binding. (A) Schematic representation of the fusion construct His_6_-MBP-[TEV]-Peptide used in this study. The positions targeted for metal coordination are highlighted in color in the TrpZip sequences. The plate layout with the color-coded positions of respective fusion peptides is shown below. (B) Screening assay results. Tb^3+^ luminescence at 545 nm emission wavelength was measured upon excitation of Trp (λ_ex_: 280 nm, blue) and 2,3-DHN (λ_ex_: 324 nm, purple), respectively. Values are averages from 12 wells with standard deviation represented in error bars. Protein concentration (red diamonds) of the elution fraction was determined by UV/vis absorption at 280 nm (average ± standard deviation). The SDS-PAGE gel shows exemplary samples of cell-free lysate (CL), cell pellet (CP), and elution (E) fraction. Parts of this figure were designed using BioRender (https://BioRender.com/m88hkof).

We first synthesized all peptide variants chemically by solid-phase peptide synthesis (SPPS), verified them by mass spectrometry (Figures S7–S10), and measured Tb^3+^ binding to Tz2H3, LanPep1 and LanPep2 in a luminescence-based titration. For LanPep1 and LanPep2, a *K*_D_ of 184 ± 9 μM and 292 ± 17 μM, respectively, was determined, while Tz2H3 showed no specific Tb^3+^ binding within the measurable range (Figure S6C). Circular dichroism (CD) spectroscopy revealed the characteristic exciton-coupled bands at 213 and 228 nm of the interacting Trp residues for TrpZip2, Tz2H3 and LanPep2, inferring a folded structure (Figure S6D). In contrast, LanPep1 was majorly unfolded, also in the presence of TbCl_3_ (Figure S6E). We reasoned that the low β-propensity of aspartate could not be counterbalanced, even in this highly stabilized fold.^66^ The effect of the repulsive acidic groups on structural integrity was also observed in CD melting curves (Figure S6F).

Despite their relatively weak lanthanide binding affinity, the set of TrpZip-derived peptides was tested in our recombinant expression-based screening assay. We fused all peptide sequences to an N-terminal His_6_-MBP tag and expressed the variants in *E. coli* (Figure 3A). Following the established procedure, we obtained elution fractions with 15–20 μM of pure fusion protein (Figure 3B). The Tb^3+^ luminescence signals of MBP-LanPep1 and MBP-LanPep2 were more than 10-fold above the background signal of the unmodified TrpZip fusions when using Trp sensitization (Figure 3B). The 2,3-DHN-sensitized signal was also distinct, but low in absolute values. To assess the potential role of the external antenna as a competing chelator, we performed further control experiments (see the Extended Discussion section including Figures S28–S30 in the Supporting Information). Overall, our findings demonstrate that the screening assay is suitable to identify lanthanide-binding to short peptides, even in the presence of the large MBP expression tag and for relatively weak lanthanide binding in the micromolar range.

### Screening a Library of De Novo Protein Scaffolds for Lanthanide Binding

The MID1 scaffold, a small helical de novo protein,^67^ was previously engineered for zinc-dependent esterase^62^ and Diels–Alderase activity,^68^ where it exhibited a pronounced structural plasticity over the course its laboratory evolution. As it also showed no detectable background signal for lanthanide binding (Figure 2A), we considered the scaffold an optimal starting point for our efforts. For scaffold library generation, we chose a semirational approach, targeting the original zinc-coordinating residues H35, H39, H61 and H65 for replacements by Asp/Glu (Figure S11). Additionally, we introduced sequence diversity by using two different template sequences: MID1sc9, the evolved esterase,^62^ and DA0, an evolutionary predecessor of the most efficient Diels–Alderase variant DA07.^68^ Both templates share 86% sequence identity and already possess a suitably positioned Trp residue (W38). The library was generated from synthetic oligo pools. The full genes were assembled by overlap-extension PCR and subcloned into an expression vector. Sanger sequencing of the whole library confirmed the nucleotide distribution at the targeted positions (Figures S12 and S13), although single variant sequencing showed some degree of mutational shuffling between the two templates, which is known for oligo pool assembly.^69^

We screened ca. 400 library members for lanthanide binding according to the established protocol (Figure S14) and characterized the four best-performing variants regarding lanthanide affinity. Three out of four hits originated from the MID1sc9 template (termed MID1sc9_4xE, MID1sc9_EEED, and MID1sc9_EDED), while one hybrid variant hit (termed MID1sc9_H1_EEED) showed shuffling with the DA0 sequence in 4 positions, namely T11I, T13S, N19K, and E32L. Titrations were performed to assess the Tb^3+^ binding affinity of the His_6_-tagged versions of all variants. Here, we observed a time-dependent luminescence signal change, which stabilized after approximately 1 h at 25 °C (Figure S15A–D). The *K*_D_ values for Tb^3+^ ranged between 29 and 209 nM (Figure S16A–D). When adding equimolar amounts of another Ln^3+^ ion to protein samples preincubated with Tb^3+^, metal ion displacements could be monitored by the decline in the Tb^3+^ luminescence signal, which provided information on the Ln^3+^ ion selectivity. All variants showed a similar trend, preferring the medium-sized Ln^3+^ ions, mainly Eu^3+^ and Sm^3+^, over the larger and smaller ones of the series. The more pronounced decline from La^3+^ to Eu^3+^ may suggest a better discrimination against the larger Ln^3+^ ions. Similar findings have also been reported for trimeric de novo coiled coils.^45^

MID1sc9_4xE and MID1sc9_EDED were selected for further analysis. To that end, we removed the His_6_-tag and worked with the tag-less variants (Figure 4 and Figure S17). Notably, the tag removal improved the *K*_D_ value for MID1sc9_4xE by 3-fold (*K*_D_ = 32 nM, Figure 4A). For MID1sc9_EDED, the affinity was too tight to be precisely determined by Tb^3+^ titrations and thus was approximated to be lower than 5 nM (Figure S17A). The lanthanide selectivity, defined as differences in relative affinity within the lanthanide series, remained the same (Figure 4B and Figure S17B). Furthermore, we determined absolute binding affinities from terbium displacement titrations of MID1sc9_4xE for La^3+^ (*K*_D_ = 258 nM), Eu^3+^ (*K*_D_ = 26 nM), and Yb^3+^ (*K*_D_ = 59 nM) (Figure S18). The nanomolar lanthanide affinities, selectivity trends, as well as the proposed 1:1 binding stoichiometry were also independently confirmed by isothermal titration calorimetry (ITC) measurements (Figures S19–S22). Lastly, we evaluated the discrimination between Ln^3+^ and Ca^2+^ ions, which can typically occupy similar coordination sites. Notably, the obtained *K*_D_ = 4.66 mM for Ca^2+^ binding of MID1sc9_4xE (Figure S18) reflects a 10^5^-fold selectivity for Ln^3+^ ions over Ca^2+^. For comparison, even up to 10^8^-fold discrimination was previously reported for lanmodulin,^26^ rationalized by its binding-responsive folding.^70^ Overall, these measurements confirmed that selective high-affinity lanthanide binders could be obtained from our semirational library design approach.

**Figure 4:**
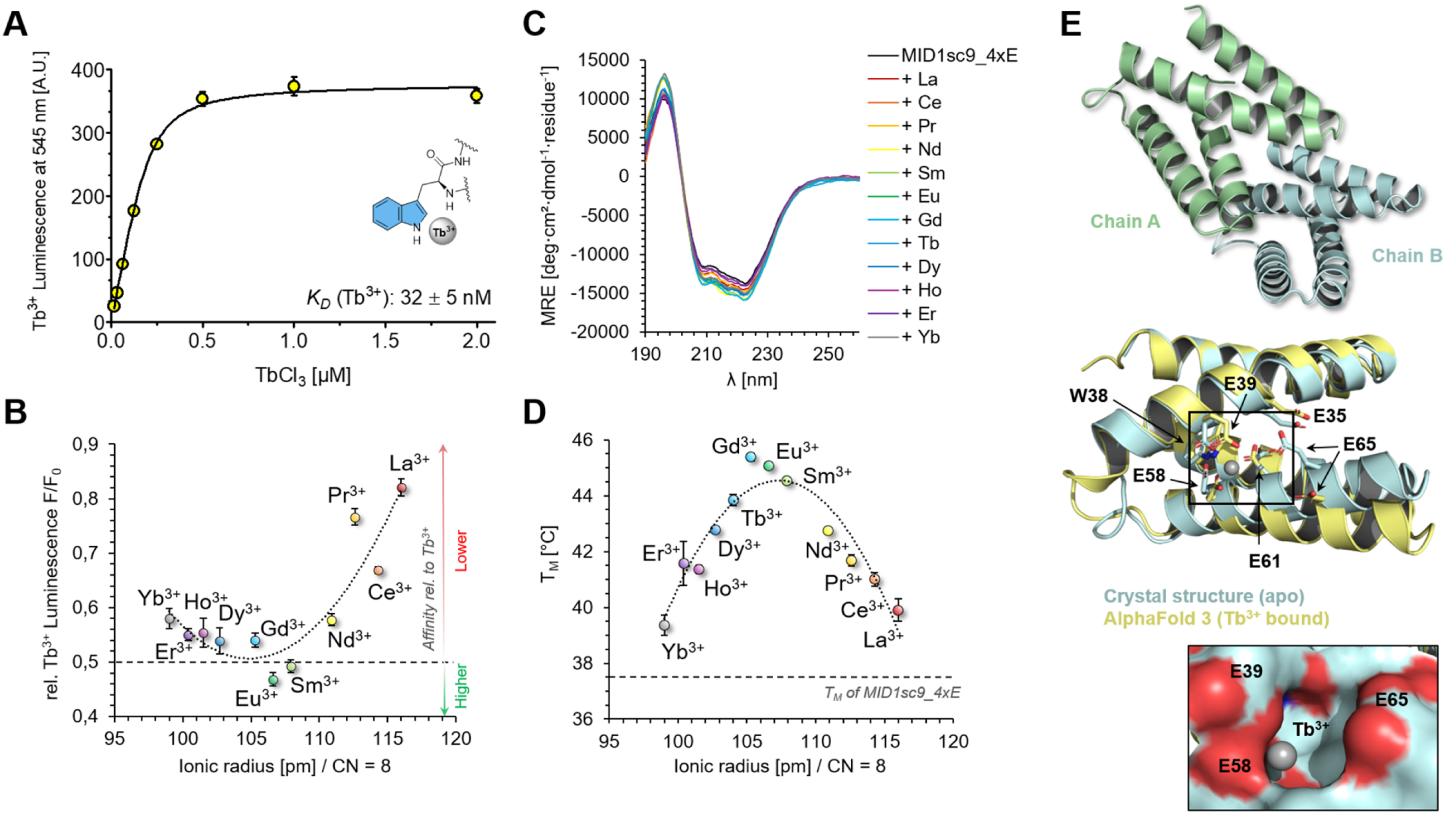
Lanthanide binding affinity, selectivity, and crystal structure of MID1sc9_4xE. (A) The Tb^3+^ affinity was measured by Trp-sensitized luminescence. 200 nM protein was titrated with 15.6 nM to 2.0 μM TbCl_3_. The apparent *K*_D_ was determined using a quadratic equation fit. (B) Ln^3+^ selectivity was assessed by Tb^3+^ displacement. An equimolar amount of LnCl_3_ was added to a 1:1 mix of 1 μM MID1sc9_4xE and TbCl_3_. The selectivity is expressed by the luminescence decrease, referenced to the signal of MID1sc9_4xE:Tb^3+^, and plotted against the ionic radii reported for octa-coordinated Ln^3+^ ions.^72^ The dashed line at 0.5 indicates that the Ln^3+^ affinity is equal to that of Tb^3+^ (*K*_D_ = 32 nM). The data are averages from technical triplicates with standard deviation given as error bars. (C) CD spectra of MID1sc9_4xE in the presence and absence of different Ln^3+^ ions. 10 μM MID1sc9_4xE was incubated with an equimolar amount of LnCl_3_ for at least 1 h prior to measurement. (D) CD-based thermal denaturation of metal-free and Ln^3+^-bound MID1sc9_4xE to determine the melting temperatures *T*_M_, which are plotted against the literature-reported ionic radii for CN = 8. The dashed line indicates the melting temperature of metal-free MID1sc9_4xE (*T*_M_: 37.5 °C). The shown data are averages from technical duplicates with standard deviation as error bars. The dotted line indicates a trend and does not represent an actual fit. (E) Crystal structure of MID1sc9_4xE (PDB entry: 9S7R) shown in cartoon representation. Below, the AlphaFold3 prediction of MID1sc9_4xE (yellow) bound to Tb^3+^ (gray sphere) was overlaid with chain A (pale blue) of the crystal structure. Residues E35, E39, W38, E58, E61, and E65 are indicated by arrows. The inset shows the surface of chain A with residues E39, E58, and E65 forming a cavity. Tb^3+^ is predicted to bind inside the cavity.

CD spectra of the proteins resembled the spectrum of the parent scaffold MID1sc9, with minor changes upon Tb^3+^ treatment (Figure 4C and Figures S17C and S23A). However, thermal denaturation experiments showed a drastic drop in melting temperature for the metal-free proteins, from 66.3 °C for the MID1sc9 parent scaffold to 37.5 and 32.9 °C for MID1sc9_4xE and MID1sc9_EDED, respectively (Figure 4D and Figures S17D and S23B). This destabilization is likely due to electrostatic repulsion of the neighboring negatively charged Asp/Glu residues, even though the helical fold remains largely intact at ambient temperature. Lanthanide binding restabilized both proteins to melting temperatures of 43.9 and 45.3 °C, respectively, in the presence of Tb^3+^. Leveraging the metal-dependent melting temperatures, we tested 12 different Ln^3+^ ions with MID1sc9_4xE and verified the preference for medium-sized ions observed in the metal displacement experiment described above (Figure 4D).

Next, we crystallized MID1sc9_4xE and determined the structure to a resolution of 1.25 Å (Figure 4E; PDB entry: 9S7R). It largely resembles the overall structure of the zinc-bound esterase variants MID1sc9 and MID1sc10 (PDB entries: 5OD9 and 5OD1) with RMSD values ranging from 1.1 to 1.5 Å, (Figure S24). The asymmetric unit contains a scaffold dimer with polar interactions in the crystallographic dimer interface (Figure S25). However, the dimeric state was not present in solution, as determined by analytical size-exclusion chromatography with static light scattering detection (SEC-SLS) in the presence and absence of TbCl_3_ (Figure S26). Notably, the parent MID1sc9 sequence contributes two more glutamates (E32 and E58) in close proximity to the putative lanthanide binding site, generating a long negatively charged cleft with overall six potentially coordinating carboxylates. Even though the protein was cocrystallized with TbCl_3_, we could not unambiguously assign electron density for a clearly positioned lanthanide ion. The absence of Tb^3+^ in the protein structure may also be a consequence of the crystallization conditions, which contained both malonate and citrate. These acids in high concentrations may compete with the protein for lanthanide binding, similarly to the phosphate treatment introduced in the next chapter. To still approximate the metal binding site location, we used *BioMetAll*,^71^ a structure-based prediction tool that identifies regions of potential metal coordination. It allocated the most probable metal binding area between the glutamate residues E35, E39, E61, and E65 (Figure S27). A Tb^3+^-bound structure predicted by AlphaFold3^32^ also positioned the Tb^3+^ ion in between the residues E39, E58 and E65 (Figure 4E), further suggesting that these residues mediate lanthanide binding. However, the precise binding geometry remained elusive.

### Phosphate Addition to Distinguish Weak and Tight Lanthanide Binders in the Screening Assay

As demonstrated above, our screening platform enables the identification of lanthanide-binding polypeptides with affinities ranging from low nM to moderate μM. However, it would be desirable to select for high-affinity binders based on the luminescence intensity. We thus adapted a strategy to enhance the screen’s stringency by adding phosphate as a competing lanthanide binder.^36^ To test this approach, we selected two of our variants, MID1sc9_H1_EEED and MID1sc9_EDED, which differ by 1 order of magnitude in binding affinity. In an initial titration experiment, increasing amounts of buffered phosphate solution were added to Tb^3+^-treated protein samples, resulting in a concentration-dependent decline of the Trp-sensitized Tb^3+^ luminescence signal. This effect was significantly more pronounced for the weaker binding MID1sc9_H1_EEED than for MID1sc9_EDED, which exhibits low nM affinity (Figure 5A). We concluded that phosphate addition may indeed allow us to wash away the low-affinity binder prior to elution, thereby discriminating between both variants in our assay. We continued with the on-resin Tb^3+^ loading plate-based workflow for both variants and included a step incubating with varying phosphate concentrations to find the optimal concentration range (Figure 5B). Here, we observed a similar trend for the relative luminescence values, indicating discrimination against the lower affinity binder in a certain phosphate concentration window of 50–100 μM (Figure 5C). Depending on the target protein, we can thus adjust the assay stringency for lanthanide binding affinities with a certain dynamic range.

**Figure 5:**
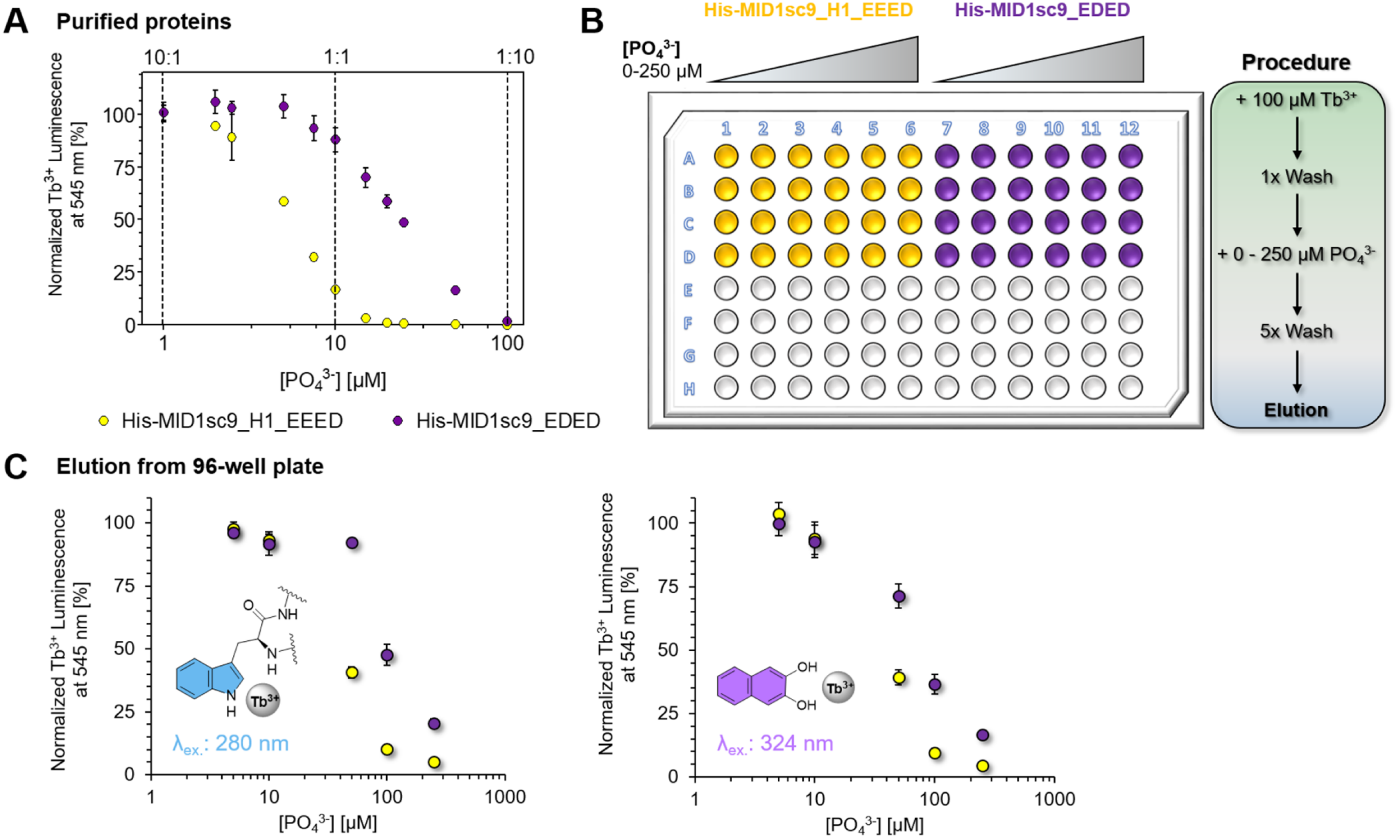
Phosphate competition to increase the stringency of the lanthanide binding assay. (A) Titration of phosphate (as a competing lanthanide binder) to MID1sc9_H1_EEED (yellow) and MID1sc9_EDED (purple) samples pretreated with TbCl_3_. 10 μM protein was mixed with 10 μM TbCl_3_ and incubated for 1 h before titration. A buffered phosphate solution was added in varying concentrations, and Tb^3+^ luminescence was recorded at λ_ex._: 280 nm and λ_em._: 545 nm. Luminescence values were normalized to a phosphate-free sample. Dashed lines indicate 10:1, 1:1, and 1:10 ratios of Tb^3+^ bound protein to phosphate. Shown averages derived from technical triplicates, with error bars representing the standard deviation. (B) Plate-based workflow with on-resin Tb^3+^ loading and addition of varying phosphate concentrations. Excess phosphate was removed by stringent washing. Colored wells indicate the protein variant. (C) Relative luminescence of the eluted proteins His-MID1sc9_H1_EEED (yellow) and His-MID1sc9_EDED (purple), after on-resin Tb^3+^ loading and phosphate treatment. The Tb^3+^ luminescence sensitized by Trp (left, λ_ex._: 280 nm) or 2,3-DHN (right, λ_ex._: 324 nm) was recorded after the addition of 100 μM 2,3-DHN to the eluted fractions. Tb^3+^ luminescence values are plotted against the phosphate concentration applied before the washing and elution steps. Averages derived from four measured wells, with error bars representing the standard deviation.

## Conclusions

We developed a robust and accessible screening platform to identify lanthanide-binding proteins and peptides in 96-well plate format. The method combines a dual Tb^3+^ luminescence readout with commercially available filter plates for affinity purification of recombinantly expressed fusion constructs and on-resin lanthanide loading. Importantly, in the absence of a suitably positioned native tryptophan in a protein of interest, several residues should be mutated to identify the optimal tryptophan position, ideally enabling efficient internal sensitization while not interfering with lanthanide binding. We demonstrated the selection of nanomolar binders from a library of de novo protein scaffolds and thoroughly characterized the best-performing variants regarding lanthanide affinity and selectivity. Our platform will enable the efficient engineering of lanthanide-utilizing biotechnological tools in the future.

## Supplementary Material



## Notes

Materials and methods, DNA and protein sequences, supplementary figures (showing lanthanide binding data, SDS gels, protein structures, LC-MS data, and sequencing data), and an extended discussion (PDF)
The crystal structure has been deposited in the RCSB Protein Data Bank (PDB entry: 9S7R). All other raw data associated with this work are publicly available via the university’s research data repository “mediaTUM” at https://doi.org/10.14459/2025mp1836402.
