# Supplementary material for: A Luminescence-Based Screening Platform for Lanthanide-Binding Peptides and Proteins
Source: ACS Chem Biol. 2025 Nov 17;20(12):2897–906. doi: 10.1021/acschembio.5c00670 (PMC12723673; doi:10.1021/acschembio.5c00670)
Supplement: Supplementary file 1 [file cb5c00670_si_001.pdf]

Supporting Information

A luminescence-based screening platform for lanthanide-binding peptides and proteins

Robert Klassen, Anna Heider, Hannah Kugler, Michael Groll, and Cathleen Zeymer*

Center for Functional Protein Assemblies & Department of Bioscience, TUM School of Natural Sciences, Technical University of Munich (TUM), 85748 Garching, Germany

* E-mail: cathleen.zeymer@tum.de

Table of Contents

1) Materials and Methods **3**

Chemicals and materials 3

Molecular cloning 3

DNA and protein sequences of all reported constructs 10

Recombinant expression and protein purification 22

Plate-based screening assay for Tb^{3+} binding 23

Excitation scans and controls evaluating the spectral crosstalk for the terbium luminescence signals sensitized by Trp and 2,3-DHN 26

Tb^{3+} titrations to determine binding affinities 27

Tb^{3+} displacement titrations with Ca^{2+} and other Ln^{3+} ions 28

Competition measurements of protein and 2,3-DHN for Tb^{3+} 29

Circular dichroism (CD) spectroscopy 30

Isothermal titration calorimetry (ITC) 31

Analytical size exclusion chromatography with static light scattering detection (SEC-SLS) 31

Mass spectrometry with peptides and proteins 31

Protein X-ray crystallography 32

Solid phase peptide synthesis 35

Prediction of the Ln^{3+} binding site in MID1sc9_4xE 36

2) Supplementary Figures **37**

3) Extended Discussion **58**

4) References **60**

1) Materials and Methods

Chemicals and materials

All chemicals were purchased from Sigma Aldrich, Carl Roth, SERVA, BLD, and Merck in p.a. grade quality and used without any further purification. All $LnCl_3$ salts were purchased as anhydrous or hexahydrate salts from Sigma Aldrich in trace metal quality (≥ 99.9%) and stock solutions of 200 mM were prepared in bidestilled H_2O and stored at -20°C until usage. DNaseI (≥ 3000 U/mg) was purchased from AppliChem GmbH. Lysozyme (≥45000 FIP U/mg) was purchased from Carl Roth. T4 ligase and BsaI_HF®v2 were purchased from New England Biolabs. *Escherichia coli* strains DH10β and BL21(DE3) GOLD were used for cloning and expression, respectively. Primers were ordered at Sigma Aldrich. Plasmid Purification Kits were obtained from Promega. HIS Select© Filter plates were purchased from Sigma Aldrich.

Molecular cloning

Synthetic genes and oligo pools were ordered from Twist Bioscience (South San Francisco, CA, USA). Fragment amplification was conducted by Touchdown-PCR. Briefly, 10 µL 5X Q5 buffer, 8.3 µL 6X Loading Dye (6% glycerol, 0.01% bromophenol blue), 1 µL 10 mM dNTP mixture (*New England Biolabs GmbH*, Frankfurt am Main, Germany), 2.5 µL 10 µM primer (**Table S2**), 2 µL Q5 high-fidelity polymerase and 1 µL template plasmid (50 – 200 $ng\cdot\mu L^{-1}$) were mixed in 50 µL total volume. The fragments were amplified with a 'touchdown' PCR program (**Table S1**). Amplification was confirmed by agarose gel electrophoresis. Template DNA was removed by DpnI digestion (37°C, 30 min, 20 U) of the PCR mixture and fragments were purified using the Wizards® SV Gel and PCR Clean-up System (*Promega GmbH*, Walldorf, Germany) or PCR Purification Kit (*Jena Bioscience GmbH*, Jena, Germany). Following cloning steps, assembly reactions were transformed into *E. coli* DH10β or BL21(DE3) GOLD via heat shock. Briefly, 2-5 µL reaction mix was added to 50 µL chemo-competent cells and kept at 42°C for 45 s. Cells were recovered in 450 µL SOC medium and incubated for 1 h at 37 °C and 750 rpm. Afterwards, transformed cells were cultivated overnight on ampicillin or kanamycin containing LB agar plates at 37 °C. Plasmid preparation was conducted with the PureYield™ MiniPrep (*Promega GmbH*, Walldorf, Germany) according to manufacturer's protocol from 5 mL LB medium (supplemented with appropriate antibiotic), inoculated with a single colony and propagated at 37°C and

180 rpm overnight. Plasmid sequences were verified by Sanger Sequencing (*GENEWIZ Germany GmbH*, Leipzig, Germany).

Table S1 | General PCR program for amplification of fragments.

Step	T	t	Cycles
**Initial Denaturation**	95°C	15 s	
**Denaturation**	95°C	15 s	
**Annealing** [a]	Primer dependent	15 s	10x
**Elongation**	72°C	30 s per kb	
**Denaturation**	95°C	15 s	
**Annealing** [a]	Primer dependent	15 s	20x
**Elongation**	72°C	30 s per kb	
**Final Elongation**	72°C	2.5 – 7 min	
**Cooling**	10°C	∞	

[a] Annealing temperature in the first cycle was set to 2-3°C above the highest T_M with 0.5-1.0°C decrement per cycle to final 2 °C below lowest T_M.

Table S2 | Primers used for cloning and mutagenesis.

Primer	Sequence (5' → 3')	Comment
1	GAACTGTATAAATAAGGTCTCATAGCTCGAGCGCTTAATTAGCTG	pQE backbone
2	AAGGTCTCTCATTCCTCCCTGAAAATACAGGTTTTCG	pQE backbone
3	GATACCGCGAGAACCACGCTCACCG	Silent mutation in *AmpR*
4	CGGTGAGCGTGGTTCTCGCGGTATC	Silent mutation in *AmpR*
5	GGCTATAACGGCCTCGCTGAAGTCGG	Silent mutation in *MBP*
6	CCGACTTCAGCGAGGCCGTTATAGCC	Silent mutation in *MBP*
7	GAAAACCTGTATTTTCAGGGAGGAATGAGAGACCTTAGCAAAGGTGAAGAACTG	sfGFP Insert, flanked by BsaI sites
8	GCTCAGCTAATTAAGCGCTCGAGCTATGAGACCTTATTTATACAGTTCATCCATACCG	sfGFP Insert, flanked by BsaI sites
9	GAAAACCTGTATTTTCAGGGA	*MBP* deletion in pQE_GGlow
10	CACCATCACGGATCTGAAAACCTGTATTTTCAGGGAGG	*MBP* deletion in pQE_GGlow
11	AGATCCGTGATGGTGATGGTG	*MBP* deletion in pQE_GGlow
12	ATGGTGATGCGATCCTCTC	*MBP* deletion in pQE_GGlow
13	ATGGTGATGCGATCCTCTCATAG	*MBP* and *TEV* deletion in pQE_GGlow
14	CACCATCACGGATCTGGAATGAGAGACCTTAGCAAAGGTG	*MBP* and *TEV* deletion in pQE_GGlow
15	GGAATGAGAGACCTTAGCAAAGGTG	*MBP* and *TEV* deletion in pQE_GGlow
16	AGATCCGTGATGGTGATGGTGATGC	*MBP* and *TEV* deletion in pQE_GGlow
17	TTGGTCTCAAATGGGTCCGCTGGCGCAGCAG	MID1sc cloning, flanked by BsaI
18	TTGGTCTCAGCTATTAGTCGGATTGTTGGAAGTACTC	MID1sc cloning, flanked by BsaI
19	CGATGGTTGGAACTTTAACGGTGTGAATGAACTCATTAG	PedH_2xMut_D323N/D325
20	TGGGGTGTCGATTGAAAATGCCATTTAATC	PedH_2xMut_D323N/D325
21	GAACTCATTAGTTTTAATTATAAAGATGGTGGAAAAG	PedH_2xMut_D323N/D325
**22**	ATTCACACCGTTAAAGTTCCAACCATCGTGGGGTGTC	PedH_2xMut_D323N/D325
23	AGCTGGACCTGGGAAAACGGCAAATGGACCTGGAAA	TrpZip2
24	TTTCCAGGTCCATTTGCCGTTTTCCCAGGTCCAGCT	TrpZip2
25	AGCTGGCATTGGGAAAACGGCAAATGGCATTGGCAT	Tz2H3
26	ATGCCAATGCCATTTGCCGTTTTCCCAATGCCAGCT	Tz2H3
27	AGCTGGGATTGGGAAAACGGCAAATGGGATTGGGAT	LanPep1
28	ATCCCAATCCCATTTGCCGTTTTCCCAATCCCAGCT	LanPep1
29	AGCTGGGAATGGGAAAACGGCAAATGGGAATGGGAA	LanPep2
30	TTCCCATTCCCATTTGCCGTTTTCCCATTCCCAGCT	LanPep2
31	TTGGTCTCAAATGCCGACGACCACCAC	LanM cloning, flanked by BsaI
32	TTGGTCTCAGCTAGCGGATCAGGTTGACCAGTGC	LanM cloning, flanked by BsaI
33	TTGGTCTCAATCGGGATCAAAAGCCGCGATATC	LanM_T41W mutation, flanked by BsaI
34	TTGGTCTCAcGATAAAGACGGTTGGATTGATCTTAAAGAGGCCTTGGC	LanM_T41W mutation, flanked by BsaI

Vector construction and Golden Gate Cloning

Initially, a Golden Gate vector was constructed by 'domesticating' the pQE vector. Fragments were amplified using primer #1-8 (**Table S2**) by PCR to 1) knock-out two off-target *BsaI* recognition sites in the *AmpR* and *MBP* gene by silent mutations, 2) introduce the codon optimized sequence of sfGFP in-frame to the IPTG-inducible expression cassette and flanked by out-warding *BsaI* recognition sites to create a reporter for green/white selection. Vector assembly was carried out using isothermal Gibson assembly (1 h, 50°C)[1]. The vector identity was verified by Sanger Sequencing and termed pQE_GGlow. Derivates of the pQE_GGlow vector missing *MBP* and *TEV* sequences were obtained by SLIM with the respective primers #9-16 (**Table S2**).

The Golden Gate cloning was performed as reported by Marillonnet and Grützner[2]. Briefly, 60 fmol fragment and 20 fmol vector was mixed with 1 µL BsaI-HF®v2 (20 $U \cdot µL^{-1}$), 1.5 µL T4 Ligase (400 $U \cdot µL^{-1}$) and 2 µL 10X Ligase buffer in 20 µL total volume and incubated isothermally at 37°C for 2 h (one fragment) or overnight (> 1 fragment). 5 µL reaction mix was then transformed into *E. coli* DH10β or BL21(DE3) GOLD by heat-shock. 50-100 µL of the inoculum was spread on LB agar (supplemented with appropriate antibiotic and 0.1 mM IPTG) and incubated at 37°C overnight. Non-glowing colonies were picked and propagated in 5 mL LB medium with appropriate antibiotic at 37°C and 180 rpm. Plasmid preparation and sequence verification was performed as previously described.

Site-directed, ligation-independent mutagenesis (SLIM)

Deletions on pQE_GGlow vector and point mutations on PedH were introduced by the SLIM method[3] with primers #9-16 and #19-22 (**Table S2**). Residual template was removed by adding DnpI (20 $U \cdot µL^{-1}$) and incubating the reaction at 37°C for 2.5 h. The hybridization was performed by mixing 10 µL of the complementary PCR products and 10 µL 5x H buffer (125 mM Tris, 750 mM NaCl, 100 mM EDTA, pH 9.0) in 50 µL final volume. The heating program was set to 3 min at 98°C for initial denaturation, followed by two cycles of 5 min at 65°C and 30°C for 40 min. 5 µL of the hybridization mixture was transformed into chemo-competent *E. coli* DH10β or BL21(DE3) GOLD via heat-shock. Plasmid preparation and sequence confirmation was conducted as described before.

Design and cloning of the MID1sc_DE library

The MID1sc_DE library was designed by splitting the genes of MID1sc9 and DA0 into two oligo pools of 143 bp and 157 bp and with a 21 bp complementary region (**Table S4**). The positions 35, 39, 61 and 65 in the amino acids sequence were permutatively replaced by codons for Glu or Asp, yielding at least 64 possible variants originating from both template sequences. Considering also some scrambling of the 14 positions in which MID1sc9 and DA0 differ, the library size could increase to 2^{18}, yielding a theoretical maximum of 262144 possible variants. The oligo pool was subjected to overlap extension PCR: A solution of 10 µL 5X Q5 buffer, 8.3 µL loading dye, 10.3 µL oligo pool (2.9 ng·µL^{-1}), 1 µL 10 mM dNTP mixture and 1 µL Q5 high-fidelity polymerase in 50 µL final volume was prepared, followed by PCR cycles shown in **Table S3**. 4 µL of the reaction mixture was subjected for 'touchdown' PCR using primer #17-18 (**Table S2**) and the purified library was cloned into pQE_GGlow vector (without *TEV* and *MBP* genes) by Golden Gate Cloning. The library composition was evaluated by sequencing whole batch and 10 single variant plasmids.

Table S3 | PCR program for overlap extension.

Step	T	t	Cycles
**Initial Denaturation**	95°C	60 s	
**Denaturation**	95°C	15 s	
**Annealing**	60°C	15 s	15x
**Elongation**	72°C	30 s per kb	
**Final Elongation**	72°C	3 min	
**Cooling**	10°C	∞	

Table S4 | Oligo pools of MID1sc_DE library. D/E mutations are highlighted in orange letters. Sequence differences between MID1sc9 and DA0 variants are highlighted in purple letters (see Figure S11). Complementary sequence region is underlined.

**Sequence (5' → 3')**
MID1sc9_ED_fw
CCGCTGGCGCAGCAGATTAAAAACACTCTGACTTTTATTGGTCAGGCGAATGCGGCGGGCCGTATGGATGAAGTGCGTACCCTGCAGGAAAACCTGGAACCGCTGTGGGAAGAATATTTTCAACAGACTGAAGGCTCTGGTGGTTCCCCTTTAGCCC
CCGCTGGCGCAGCAGATTAAAAACACTCTGACTTTTATTGGTCAGGCGAATGCGGCGGGCCGTATGGATGAAGTGCGTACCCTGCAGGAAAACCTGGAACCGCTGTGGGATGAATATTTTCAACAGACTGAAGGCTCTGGTGGTTCCCCTTTAGCCC
CCGCTGGCGCAGCAGATTAAAAACACTCTGACTTTTATTGGTCAGGCGAATGCGGCGGGCCGTATGGATGAAGTGCGTACCCTGCAGGAAAACCTGGATCCGCTGTGGGAAGAATATTTTCAACAGACTGAAGGCTCTGGTGGTTCCCCTTTAGCCC
CCGCTGGCGCAGCAGATTAAAAACACTCTGACTTTTATTGGTCAGGCGAATGCGGCGGGCCGTATGGATGAAGTGCGTACCCTGCAGGAAAACCTGGATCCGCTGTGGGATGAATATTTTCAACAGACTGAAGGCTCTGGTGGTTCCCCTTTAGCCC
DA0_ED_fw
CCGCTGGCGCAGCAGATTAAAAACATTCTGTCTTTTATTGGTCAGGCGAAAGCGGCGGGCCGTATGGATGAAGTGCGTACCCTGCAGCTGAACCTGGAACCGCTGTGGGAAGAATATTGGCAACAGTCTGATGGCTCTGGTGGTTCCCCTTTAGCCC
CCGCTGGCGCAGCAGATTAAAAACATTCTGTCTTTTATTGGTCAGGCGAAAGCGGCGGGCCGTATGGATGAAGTGCGTACCCTGCAGCTGAACCTGGAACCGCTGTGGGATGAATATTGGCAACAGTCTGATGGCTCTGGTGGTTCCCCTTTAGCCC
CCGCTGGCGCAGCAGATTAAAAACATTCTGTCTTTTATTGGTCAGGCGAAAGCGGCGGGCCGTATGGATGAAGTGCGTACCCTGCAGCTGAACCTGGATCCGCTGTGGGAAGAATATTGGCAACAGTCTGATGGCTCTGGTGGTTCCCCTTTAGCCC
CCGCTGGCGCAGCAGATTAAAAACATTCTGTCTTTTATTGGTCAGGCGAAAGCGGCGGGCCGTATGGATGAAGTGCGTACCCTGCAGCTGAACCTGGATCCGCTGTGGGATGAATATTGGCAACAGTCTGATGGCTCTGGTGGTTCCCCTTTAGCCC
MID1sc9_ED_rev
GTCGGATTGTTGGAAGTACTCTTTCATTAATTGGAGCGTATTCTCCGATAAACGGCGAACCTCGTCCATGCGACCGGCGGCTCTGGCTTGTTCGATGAGAACTTCGCCATACTCGATTTGTTGGGCTAAAGGGGAACCACCAG
GTCGGATTGTTGGAAGTACTCTTTCATTAATTGGAGCGTATTCTCCGATAAACGGCGAACCTCGTCCATGCGACCGGCGGCTCTGGCTTGTTCGATGAGAACATCGCCATACTCGATTTGTTGGGCTAAAGGGGAACCACCAG
GTCGGATTGTTGGAAGTACTCTTTCATTAATTGGAGCGTATTCTCCGATAAACGGCGAACCTCGTCCATGCGACCGGCGGCTCTGGCTTGATCGATGAGAACTTCGCCATACTCGATTTGTTGGGCTAAAGGGGAACCACCAG
GTCGGATTGTTGGAAGTACTCTTTCATTAATTGGAGCGTATTCTCCGATAAACGGCGAACCTCGTCCATGCGACCGGCGGCTCTGGCTTGATCGATGAGAACATCGCCATACTCGATTTGTTGGGCTAAAGGGGAACCACCAG

DA0_ED_rev
GTCGGATTGTTGGAAGTACTCTTTCATTAATTGGAGCACATTCTCACGTAACTTGCGAACCTCGTCCATGCGACCGGCGGCCCAGGCTTGTTCGATGAAAATTTCGCCATTCTCGATTTGTTGGGCTAAAGGGGAACCACCAG
GTCGGATTGTTGGAAGTACTCTTTCATTAATTGGAGCACATTCTCACGTAACTTGCGAACCTCGTCCATGCGACCGGCGGCCCAGGCTTGTTCGATGAAAATATCGCCATTCTCGATTTGTTGGGCTAAAGGGGAACCACCAG
GTCGGATTGTTGGAAGTACTCTTTCATTAATTGGAGCACATTCTCACGTAACTTGCGAACCTCGTCCATGCGACCGGCGGCCCAGGCTTGATCGATGAAAATTTCGCCATTCTCGATTTGTTGGGCTAAAGGGGAACCACCAG
GTCGGATTGTTGGAAGTACTCTTTCATTAATTGGAGCACATTCTCACGTAACTTGCGAACCTCGTCCATGCGACCGGCGGCCCAGGCTTGATCGATGAAAATATCGCCATTCTCGATTTGTTGGGCTAAAGGGGAACCACCAG

Cloning of MBP-tagged peptide constructs

20 µL of the respective primer pair (100 µM, primer #23-30, **Table S2**) were mixed and a hybridization reaction was conducted on the thermocycler with following program: 3 min at 98°C for initial denaturation, 2 cycles of 5 min at 65°C and 40 min at 30°C. The pQE vector and hybridized primer were digested (37°C, 2 h) with 1 µL HindIII and 1 µL BamHI in CutSmart buffer in 50 µL final volume. Following agarose gel electrophoresis, the corresponding bands were excised and purified by using the Monarch® DNA Gel Extraction Kit (*New England Biolabs* GmbH, Frankfurt am Main, Germany). The ligation was performed with 50 ng linearized vector, 250 ng fragment and 1 µL T4 Ligase (400 U·µL^{-1}) in commercial ligase buffer in 20 µL total volume and incubating at room temperature for 1 h. 5 µL of the ligation mixture was transformed into chemo-competent *E. coli* DH10β via heat-shock. Plasmid preparation and sequence confirmation was conducted as described above.

DNA and protein sequences of all reported constructs

His$_6$-MBP-TEV-*sf*GFP (pQE vector)

His$_6$ tag MBP tag TEV protease cleavage site *sf*GFP

ATGAGAGGATCGCATCACCATCACCATCACGGATCTAAAATCGAAGAAGGTAAACTGGTAATCTG
GATTAACGGCGATAAAGGCTATAACGGCCTCGCTGAAGTCGGTAAGAAATTCGAGAAAGATACCG
GAATTAAAGTCACCGTTGAGCATCCGGATAAACTGGAAGAGAAATTCCCACAGGTTGCGGCAACT
GGCGATGGCCCTGACATTATCTTCTGGGCACACGACCGCTTTGGTGGCTACGCTCAATCTGGCC
TGTTGGCTGAAATCACCCCGGACAAAGCGTTCCAGGACAAGCTGTATCCGTTTACCTGGGATGC
CGTACGTTACAACGGCAAGCTGATTGCTTACCCGATCGCTGTTGAAGCGTTATCGCTGATTTATA
ACAAAGACCTGCTGCCGAACCCGCCAAAAACCTGGGAAGAGATCCCGGCGCTGGATAAAGAACT
GAAAGCGAAAGGTAAGAGCGCGCTGATGTTCAACCTGCAAGAACCGTACTTCACCTGGCCGCTG
ATTGCTGCTGACGGGGGTTATGCGTTCAAGTATGAAAACGGCAAGTACGACATTAAAGACGTGG
GCGTGGATAACGCTGGCGCGAAAGCGGGTCTGACCTTCCTGGTTGACCTGATTAAAAACAAACA
CATGAATGCAGACACCGATTACTCCATCGCAGAAGCTGCCTTTAATAAAGGCGAAACAGCGATGA
CCATCAACGGCCCGTGGGCATGGTCCAACATCGACACCAGCAAAGTGAATTATGGTGTAACGGT
ACTGCCGACCTTCAAGGGTCAACCATCCAAACCGTTCGTTGGCGTGCTGAGCGCAGGTATTAAC
GCCGCCAGTCCGAACAAAGAGCTGGCAAAAGAGTTCCTCGAAAACTATCTGCTGACTGATGAAG
GTCTGGAAGCGGTTAATAAAGACAAACCGCTGGGTGCCGTAGCGCTGAAGTCTTACGAGGAAGA
GTTGGCGAAAGATCCACGTATTGCCGCCACTATGGAAAACGCCCAGAAAGGTGAAATCATGCCG
AACATCCCGCAGATGTCCGCTTTCTGGTATGCCGTGCGTACTGCGGTGATCAACGCCGCCAGCG
GTCGTCAGACTGTCGATGAAGCCCTGAAAGACGCGCAGACTAATTCGAGCTCGAACAACAACAA
CAATAACAATAACAACAACCTCGGGATCGAGGGAACGACCGAAAACCTGTATTTTCAGGGAGGAA
TGAGAGACCTTAGCAAAGGTGAAGAACTGTTTACCGGCGTTGTGCCGATTCTGGTGGAACTGGAT
GGTGATGTGAATGGCCATAAATTTAGCGTTCGTGGCGAAGGCGAAGGTGATGCGACCAACGGTA
AACTGACCCTGAAATTTATTTGCACCACCGGTAAACTGCCGGTTCCGTGGCCGACCCTGGTGACC
ACCCTGACCTATGGCGTTCAGTGCTTTAGCCGCTATCCGGATCATATGAAACGCCATGATTTCTTT
AAAAGCGCGATGCCGGAAGGCTATGTGCAGGAACGTACCATTAGCTTCAAAGATGATGGCACCT
ATAAAACCCGTGCGGAAGTTAAATTTGAAGGCGATACCCTGGTGAACCGCATTGAACTGAAAGGT
ATTGATTTTAAAGAAGATGGCAACATTCTGGGTCATAAACTGGAATATAATTTCAACAGCCATAAC
GTGTATATTACCGCCGATAAACAGAAAAATGGCATCAAAGCGAACTTTAAAATCCGTCACAACGTG
GAAGATGGTAGCGTGCAGCTGGCGGATCATTATCAGCAGAATACCCCGATTGGTGATGGCCCGG
TGCTGCTGCCGGATAATCATTATCTGAGCACCCAGAGCGTTCTGAGCAAAGATCCGAATGAAAAA
CGTGATCATATGGTGCTGCTGGAATTTGTTACCGCCGCGGGCATTACCCACGGTATGGATGAACT
GTATAAATAA

MW: 71852 Da $\varepsilon_{280\ nm}$: 86750 $M^{-1}\cdot cm^{-1}$

MRGSHHHHHHGSKIEEGKLVIWINGDKGYNGLAEVGKKFEKDTGIKVTVEHPDKLEEKFPQVAATGD
GPDIIFWAHDRFGGYAQSGLLAEITPDKAFQDKLYPFTWDAVRYNGKLIAYPIAVEALSLIYNKDLLPNP
PKTWEEIPALDKELKAKGKSALMFNLQEPYFTWPLIAADGGYAFKYENGKYDIKDVGVDNAGAKAGLT
FLVDLIKNKHMNADTDYSIAEAAFNKGETAMTINGPWAWSNIDTSKVNYGVTVLPTFKGQPSKPFVGV
LSAGINAASPNKELAKEFLENYLLTDEGLEAVNKDKPLGAVALKSYEEELAKDPRIAATMENAQKGEIM
PNIPQMSAFWYAVRTAVINAASGRQTVDEALKDAQTNSSSNNNNNNNNNLGIEGTTENLYFQGGM
RDLSKGEELFTGVVPILVELDGDVNGHKFSVRGEGEGDATNGKLTLKFICTTGKLPVPWPTLVTTLTY
GVQCFSRYPDHMKRHDFFKSAMPEGYVQERTISFKDDGTYKTRAEVKFEGDTLVNRIELKGIDFKED
GNILGHKLEYNFNSHNVYITADKQKNGIKANFKIRHNVEDGSVQLADHYQQNTPIGDGPVLLPDNHYL
STQSVLSKDPNEKRDHMVLLEFVTAAGITHGMDELYK

His6-TEV-*sf*GFP (pQE vector)

His6 tag TEV protease cleavage site *sf*GFP

ATGAGAGGATCGCATCACCATCACCATCACGGATCTGAAAACCTGTATTTTCAGGGAGGAATGAG
AGACCTTAGCAAAGGTGAAGAACTGTTTACCGGCGTTGTGCCGATTCTGGTGGAACTGGATGGT
GATGTGAATGGCCATAAATTTAGCGTTCGTGGCGAAGGCGAAGGTGATGCGACCAACGGTAAAC
TGACCCTGAAATTTATTTGCACCACCGGTAAACTGCCGGTTCCGTGGCCGACCCTGGTGACCAC
CCTGACCTATGGCGTTCAGTGCTTTAGCCGCTATCCGGATCATATGAAACGCCATGATTTCTTTAA
AAGCGCGATGCCGGAAGGCTATGTGCAGGAACGTACCATTAGCTTCAAAGATGATGGCACCTAT
AAAACCCGTGCGGAAGTTAAATTTGAAGGCGATACCCTGGTGAACCGCATTGAACTGAAAGGTAT
TGATTTTAAAGAAGATGGCAACATTCTGGGTCATAAACTGGAATATAATTTCAACAGCCATAACGT
GTATATTACCGCCGATAAACAGAAAAATGGCATCAAAGCGAACTTTAAAATCCGTCACAACGTGG
AAGATGGTAGCGTGCAGCTGGCGGATCATTATCAGCAGAATACCCCGATTGGTGATGGCCCGGT
GCTGCTGCCGGATAATCATTATCTGAGCACCCAGAGCGTTCTGAGCAAAGATCCGAATGAAAAAC
GTGATCATATGGTGCTGCTGGAATTTGTTACCGCCGCGGGCATTACCCACGGTATGGATGAACTG
TATAAATAA

MW: 29473 Da $\varepsilon_{280\ nm}$: 20400 $M^{-1} \cdot cm^{-1}$

MRGSHHHHHHGSENLYFQGGMRDLSKGEELFTGVVPILVELDGDVNGHKFSVRGEGEGDATNGKLT
LKFICTTGKLPVPWPTLVTTLTYGVQCFSRYPDHMKRHDFFKSAMPEGYVQERTISFKDDGTYKTRAE
VKFEGDTLVNRIELKGIDFKEDGNILGHKLEYNFNSHNVYITADKQKNGIKANFKIRHNVEDGSVQLAD
HYQQNTPIGDGPVLLPDNHYLSTQSVLSKDPNEKRDHMVLLEFVTAAGITHGMDELYK

His6-*sf*GFP (pQE vector)

His6 tag *sf*GFP

ATGAGAGGATCGCATCACCATCACCATCACGGATCTGGAATGAGAGACCTTAGCAAAGGTGAAG
AACTGTTTACCGGCGTTGTGCCGATTCTGGTGGAACTGGATGGTGATGTGAATGGCCATAAATTT
AGCGTTCGTGGCGAAGGCGAAGGTGATGCGACCAACGGTAAACTGACCCTGAAATTTATTTGCA
CCACCGGTAAACTGCCGGTTCCGTGGCCGACCCTGGTGACCACCCTGACCTATGGCGTTCAGTG
CTTTAGCCGCTATCCGGATCATATGAAACGCCATGATTTCTTTAAAAGCGCGATGCCGGAAGGCT
ATGTGCAGGAACGTACCATTAGCTTCAAAGATGATGGCACCTATAAAACCCGTGCGGAAGTTAAA
TTTGAAGGCGATACCCTGGTGAACCGCATTGAACTGAAAGGTATTGATTTTAAAGAAGATGGCAA
CATTCTGGGTCATAAACTGGAATATAATTTCAACAGCCATAACGTGTATATTACCGCCGATAAACA
GAAAAATGGCATCAAAGCGAACTTTAAAATCCGTCACAACGTGGAAGATGGTAGCGTGCAGCTGG
CGGATCATTATCAGCAGAATACCCCGATTGGTGATGGCCCGGTGCTGCTGCCGGATAATCATTAT
CTGAGCACCCAGAGCGTTCTGAGCAAAGATCCGAATGAAAAACGTGATCATATGGTGCTGCTGG
AATTTGTTACCGCCGCGGGCATTACCCACGGTATGGATGAACTGTATAAATAA

MW: 28621 Da $\varepsilon_{280\ nm}$: 18910 $M^{-1} \cdot cm^{-1}$

MRGSHHHHHHGSGMRDLSKGEELFTGVVPILVELDGDVNGHKFSVRGEGEGDATNGKLTLKFICTT
GKLPVPWPTLVTTLTYGVQCFSRYPDHMKRHDFFKSAMPEGYVQERTISFKDDGTYKTRAEVKFEGD
TLVNRIELKGIDFKEDGNILGHKLEYNFNSHNVYITADKQKNGIKANFKIRHNVEDGSVQLADHYQQNT
PIGDGPVLLPDNHYLSTQSVLSKDPNEKRDHMVLLEFVTAAGITHGMDELYK

PedH-His6 (wildtype) (pET29b vector)

His6 tag PedH

ATGGCAGTGTCTAACGAGGAGATTCTGCAAGATCCAAAGAATCCACAACAAATTGTAACGAACGG
ATTAGGAGTACAAGGTCAACGTTATTCGCCTTTGGATTTGCTGAACGTTAACAATGTGAAAGAATT
GCGTCCTGTATGGGCTTTCTCGTTTGGTGGCGAGAAACAACGTGGTCAACAAGCGCAACCATTAA
TTAAAGATGGCGTTATGTATCTCACGGGAAGCTATAGCCGCGTATTTGCGGTTGACGCACGTACT
GGTAAGAAGTTGTGGCAGTATGACGCCCGTCTCCCCGACGATATTCGTCCATGTTGTGATGTTAT
TAATCGTGGGGTGGCCCTTTATGGTAATTTAGTATTCTTTGGAACTCTCGATGCGAAACTTGTAGC
GCTTAATAAAGATACGGGTAAAGTAGTTTGGTCAAAGAAAGTAGCGGATCATAAAGAGGGATATT
CGATTAGTGCTGCCCCCATGATTGTGAACGGAAAATTGATTACCGGTGTAGCAGGTGGTGAATTT
GGGGTTGTCGGGAAAATTCAAGCATATAATCCCGAAAATGGTGAGTTATTGTGGATGCGTCCTAC
AGTCGAGGGCCACATGGGTTACGTATATAAAGACGGTAAAGCTATTGAAAATGGCATCAGTGGCG
GCGAAGCCGGGAAAACGTGGCCAGGGGATCTCTGGAAAACTGGTGGTGCGGCCCCCTGGTTAG
GCGGCTATTATGATCCGGAGACGAATTTGATTTTATTCGGGACGGGGAATCCTGCACCTTGGAAT
AGCCATTTGCGTCCAGGCGATAATCTTTATAGCTCGTCACGTCTTGCCCTTAATCCAGATGATGGT
ACGATTAAATGGCATTTTCAATCGACACCCCACGATGGTTGGGATTTTGATGGTGTGAATGAACTC
ATTAGTTTTAATTATAAAGATGGTGGAAAAGAAGTGAAAGCAGCAGCTACCGCGGATCGTAATGG
CTTCTTCTATGTATTAGATCGTACGAATGGTAAATTTATTCGTGGTTTTCCTTTTGTAGATAAAATTA
CTTGGGCGACCGGTCTGGATAAAGATGGGCGCCCCATTTATAATGATGCAAGTCGTCCTGGGGC
CCCGGGGTCGGAAGCGAAAGGGTCATCCGTATTTGTGGCCCCAGCGGTACTGGGTGCGAAGAA
TTGGATGCCAATGGCGTATAATAAAGATACGGGCTTGTTTTATGTACCAAGTAATGAATGGGGAAT
GGATATTTGGAATGAGGGAATTGCATACAAGAAGGGCGCCGCCTTTCTGGGCGCGGGGTTTACT
ATTAAACCACTTAACGAGGATTATATTGGTGTTCTTCGTGCAATTGATCCCGTTTCCGGGAAAGAG
GTCTGGCGTCATAAGAATTACGCCCCATTATGGGGTGGCGTTCTTACTACGAAAGGGAATTTAGT
GTTTACTGGAACTCCTGAAGGGTTTCTTCAAGCGTTTAATGCCAAAACTGGGGATAAAGTTTGGG
AGTTTCAAACTGGTAGCGGTGTATTGGGGTCACCGGTGACGTGGGAGATGGATGGTGAACAGTA
TGTCTCCGTGGTGTCGGGTTGGGGTGGTGCAGTCCCTTTAGCGGGTGGAGAGGTAGCGAAGCG
TGTGAAAGATTTTAATCAAGGTGGAATGTTGTGGACATTTAAACTGCCGAAACAACTGCAACAGAC
CGCCTCGGTTAAACCGCTCGAGCACCACCACCACCACCACTGA

MW: 63097 Da $\varepsilon_{280\ nm}$: 141290 $M^{-1}\cdot cm^{-1}$

MAVSNEEILQDPKNPQQIVTNGLGVQGQRYSPLDLLNVNNVKELRPVWAFSFGGEKQRGQQAQPLIK
DGVMYLTGSYSRVFAVDARTGKKLWQYDARLPDDIRPCCDVINRGVALYGNLVFFGTLDAKLVALNK
DTGKVVWSKKVADHKEGYSISAAPMIVNGKLITGVAGGEFGVVGKIQAYNPENGELLWMRPTVEGHM
GYVYKDGKAIENGISGGEAGKTWPGDLWKTGGAAPWLGGYYDPETNLILFGTGNPAPWNSHLRPGD
NLYSSSRLALNPDDGTIKWHFQSTPHDGWDFDGVNELISFNYKDGGKEVKAAATADRNGFFYVLDRT
NGKFIRGFPFVDKITWATGLDKDGRPIYNDASRPGAPGSEAKGSSVFVAPAVLGAKNWMPMAYNKDT
GLFYVPSNEWGMDIWNEGIAYKKGAAFLGAGFTIKPLNEDYIGVLRAIDPVSGKEVWRHKNYAPLWG
GVLTTKGNLVFTGTPEGFLQAFNAKTGDKVWEFQTGSGVLGSPVTWEMDGEQYVSVVSGWGGAVP
LAGGEVAKRVKDFNQGGMLWTFKLPKQLQQTASVKPLEHHHHHH

PedH_2xMut-His$_6$ (D323N D325N) (pET29b vector)

His$_6$ tag Mutation position PedH_2xMut

ATGGCAGTGTCTAACGAGGAGATTCTGCAAGATCCAAAGAATCCACAACAAATTGTAACGAACGG
ATTAGGAGTACAAGGTCAACGTTATTCGCCTTTGGATTTGCTGAACGTTAACAATGTGAAAGAATT
GCGTCCTGTATGGGCTTTCTCGTTTGGTGGCGAGAAACAACGTGGTCAACAAGCGCAACCATTAA
TTAAAGATGGCGTTATGTATCTCACGGGAAGCTATAGCCGCGTATTTGCGGTTGACGCACGTACT
GGTAAGAAGTTGTGGCAGTATGACGCCCGTCTCCCCGACGATATTCGTCCATGTTGTGATGTTAT
TAATCGTGGGGTGGCCCTTTATGGTAATTTAGTATTCTTTGGAACTCTCGATGCGAAACTTGTAGC
GCTTAATAAAGATACGGGTAAAGTAGTTTGGTCAAAGAAAGTAGCGGATCATAAAGAGGGATATT
CGATTAGTGCTGCCCCCATGATTGTGAACGGAAAATTGATTACCGGTGTAGCAGGTGGTGAATTT
GGGGTTGTCGGGAAAATTCAAGCATATAATCCCGAAAATGGTGAGTTATTGTGGATGCGTCCTAC
AGTCGAGGGCCACATGGGTTACGTATATAAAGACGGTAAAGCTATTGAAAATGGCATCAGTGGCG
GCGAAGCCGGGAAAACGTGGCCAGGGGATCTCTGGAAAACTGGTGGTGCGGCCCCCTGGTTAG
GCGGCTATTATGATCCGGAGACGAATTTGATTTTATTCGGGACGGGGAATCCTGCACCTTGGAAT
AGCCATTTGCGTCCAGGCGATAATCTTTATAGCTCGTCACGTCTTGCCCTTAATCCAGATGATGGT
ACGATTAAATGGCATTTTCAATCGACACCCCACGATGGTTGGAACTTTAACGGTGTGAATGAACTC
ATTAGTTTTAATTATAAAGATGGTGGAAAAGAAGTGAAAGCAGCAGCTACCGCGGATCGTAATGG
CTTCTTCTATGTATTAGATCGTACGAATGGTAAATTTATTCGTGGTTTTCCTTTTGTAGATAAAATTA
CTTGGGCGACCGGTCTGGATAAAGATGGGCGCCCCATTTATAATGATGCAAGTCGTCCTGGGGC
CCCGGGGTCGGAAGCGAAAGGGTCATCCGTATTTGTGGCCCCAGCGGTACTGGGTGCGAAGAA
TTGGATGCCAATGGCGTATAATAAAGATACGGGCTTGTTTTATGTACCAAGTAATGAATGGGGAAT
GGATATTTGGAATGAGGGAATTGCATACAAGAAGGGCGCCGCCTTTCTGGGCGCGGGGTTTACT
ATTAAACCACTTAACGAGGATTATATTGGTGTTCTTCGTGCAATTGATCCCGTTTCCGGGAAAGAG
GTCTGGCGTCATAAGAATTACGCCCCATTATGGGGTGGCGTTCTTACTACGAAAGGGAATTTAGT
GTTTACTGGAACTCCTGAAGGGTTTCTTCAAGCGTTTAATGCCAAAACTGGGGATAAAGTTTGGG
AGTTTCAAACTGGTAGCGGTGTATTGGGGTCACCGGTGACGTGGGAGATGGATGGTGAACAGTA
TGTCTCCGTGGTGTCGGGTTGGGGTGGTGCAGTCCCTTTAGCGGGTGGAGAGGTAGCGAAGCG
TGTGAAAGATTTTAATCAAGGTGGAATGTTGTGGACATTTAAACTGCCGAAACAACTGCAACAGAC
CGCCTCGGTTAAACCGCTCGAGCACCACCACCACCACCACTGA

MW: 63095 Da $\varepsilon_{280\ nm}$: 141290 $M^{-1}\cdot cm^{-1}$

MAVSNEEILQDPKNPQQIVTNGLGVQGQRYSPLDLLNVNNVKELRPVWAFSFGGEKQRGQQAQPLIK
DGVMYLTGSYSRVFAVDARTGKKLWQYDARLPDDIRPCCDVINRGVALYGNLVFFGTLDAKLVALNK
DTGKVVWSKKVADHKEGYSISAAPMIVNGKLITGVAGGEFGVVGKIQAYNPENGELLWMRPTVEGHM
GYVYKDGKAIENGISGGEAGKTWPGDLWKTGGAAPWLGGYYDPETNLILFGTGNPAPWNSHLRPGD
NLYSSSRLALNPDDGTIKWHFQSTPHDGWNFNGVNELISFNYKDGGKEVKAAATADRNGFFYVLDRT
NGKFIRGFPFVDKITWATGLDKDGRPIYNDASRPGAPGSEAKGSSVFVAPAVLGAKNWMPMAYNKDT
GLFYVPSNEWGMDIWNEGIAYKKGAAFLGAGFTIKPLNEDYIGVLRAIDPVSGKEVWRHKNYAPLWG
GVLTTKGNLVFTGTPEGFLQAFNAKTGDKVWEFQTGSGVLGSPVTWEMDGEQYVSVVSGWGGAVP
LAGGEVAKRVKDFNQGGMLWTFKLPKQLQQTASVKPLEHHHHHH

His$_6$-MID1sc9 (pQE vector)

His$_6$ tag D/E mutation position MID1sc9

ATGAGAGGATCGCATCACCATCACCATCACGGATCTGGAATGGGTCCGCTGGCGCAGCAGATTA
AAAACACTCTGACTTTTATTGGTCAGGCGAATGCGGCGGGCCGTATGGATGAAGTGCGTACCCT
GCAGGAAAACCTGCATCCGCTGTGGCATGAATATTTTCAACAGACTGAAGGCTCTGGTGGTTCCC
CTTTAGCCCAACAAATCGAGTATGGCCACGTTCTCATCCACCAAGCCAGAGCCGCCGGTCGCAT
GGACGAGGTTCGCCGTTTATCGGAGAATACGCTCCAATTAATGAAAGAGTACTTCCAACAATCCG
ACTAA

MW: 12282 Da $\varepsilon_{280\ nm}$: 9970 $M^{-1}\cdot cm^{-1}$

MRGSHHHHHHGSGMGPLAQQIKNTLTFIGQANAAGRMDEVRTLQENLHPLWHEYFQQTEGSGGSP
LAQQIEYGHVLIHQARAAGRMDEVRRLSENTLQLMKEYFQQSD

His$_6$-TEV-MID1sc9 (pQE vector)

His$_6$ tag TEV protease cleavage site D/E mutation position MID1sc9

ATGAGAGGATCGCATCACCATCACCATCACGGATCTGAAAACCTGTATTTTCAGGGAGGAATGGG
TCCGCTGGCGCAGCAGATTAAAAACACTCTGACTTTTATTGGTCAGGCGAATGCGGCGGGCCGT
ATGGATGAAGTGCGTACCCTGCAGGAAAACCTGCATCCGCTGTGGCATGAATATTTTCAACAGAC
TGAAGGCTCTGGTGGTTCCCCTTTAGCCCAACAAATCGAGTATGGCCACGTTCTCATCCACCAAG
CCAGAGCCGCCGGTCGCATGGACGAGGTTCGCCGTTTATCGGAGAATACGCTCCAATTAATGAA
AGAGTACTTCCAACAATCCGACTAA

MW: 13134 Da $\varepsilon_{280\ nm}$: 11460 $M^{-1}\cdot cm^{-1}$

MRGSHHHHHHGSENLYFQGGMGPLAQQIKNTLTFIGQANAAGRMDEVRTLQENLHPLWHEYFQQT
EGSGGSPLAQQIEYGHVLIHQARAAGRMDEVRRLSENTLQLMKEYFQQSD

after TEV cleavage

MW: 10940 Da $\varepsilon_{280\ nm}$: 9970 $M^{-1}\cdot cm^{-1}$

GGMGPLAQQIKNTLTFIGQANAAGRMDEVRTLQENLHPLWHEYFQQTEGSGGSPLAQQIEYGHVLIH
QARAAGRMDEVRRLSENTLQLMKEYFQQSD

His$_6$-TEV-MID1sc9_4xE (pQE vector)

His$_6$ tag TEV protease cleavage site D/E mutation position MID1sc9_4xE

ATGAGAGGATCGCATCACCATCACCATCACGGATCTGAAAACCTGTATTTTCAGGGAGGAATGGG
TCCGCTGGCGCAGCAGATTAAAAACACTCTGACTTTTATTGGTCAGGCGAATGCGGCGGGCCGT
ATGGATGAAGTGCGTACCCTGCAGGAAAACCTGGAACCGCTGTGGGAAGAATATTTTCAACAGAC
TGAAGGCTCTGGTGGTTCCCCTTTAGCCCAACAAATCGAGTATGGCGAAGTTCTCATCGAACAAG
CCAGAGCCGCCGGTCGCATGGACGAGGTTCGCCGTTTATCGGAGAATACGCTCCAATTAATGAA
AGAGTACTTCCAACAATCCGACTAA

MW: 13102 Da $\varepsilon_{280\ nm}$: 11460 $M^{-1}\cdot cm^{-1}$

MRGSHHHHHHGSENLYFQGGMGPLAQQIKNTLTFIGQANAAGRMDEVRTLQENLEPLWEEYFQQTE
GSGGSPLAQQIEYGEVLIEQARAAGRMDEVRRLSENTLQLMKEYFQQSD

after TEV cleavage

MW: 10908 Da $\varepsilon_{280\ nm}$: 9970 $M^{-1}\cdot cm^{-1}$

GGMGPLAQQIKNTLTFIGQANAAGRMDEVRTLQENLEPLWEEYFQQTEGSGGSPLAQQIEYGEVLIE
QARAAGRMDEVRRLSENTLQLMKEYFQQSD

His$_6$-TEV-MID1sc9_EDED (pQE vector)

His$_6$ tag TEV protease cleavage site D/E mutation position MID1sc9_EDED

ATGAGAGGATCGCATCACCATCACCATCACGGATCTGAAAACCTGTATTTTCAGGGAGGAATGGG
TCCGCTGGCGCAGCAGATTAAAAACACTCTGACTTTTATTGGTCAGGCGAATGCGGCGGGCCGT
ATGGATGAAGTGCGTACCCTGCAGGAAAACCTGGAACCGCTGTGGGATGAATATTTTCAACAGAC
TGAAGGCTCTGGTGGTTCCCCTTTAGCCCAACAAATCGAGTATGGCGAAGTTCTCATCGATCAAG
CCAGAGCCGCCGGTCGCATGGACGAGGTTCGCCGTTTATCGGAGAATACGCTCCAATTAATGAA
AGAGTACTTCCAACAATCCGACTAA

MW: 13073 Da $\varepsilon_{280\ nm}$: 11460 $M^{-1}\cdot cm^{-1}$

MRGSHHHHHHGSENLYFQGGMGPLAQQIKNTLTFIGQANAAGRMDEVRTLQENLEPLWDEYFQQTE
GSGGSPLAQQIEYGEVLIDQARAAGRMDEVRRLSENTLQLMKEYFQQSD

after TEV cleavage

MW: 10880 Da $\varepsilon_{280\ nm}$: 9970 $M^{-1}\cdot cm^{-1}$

GGMGPLAQQIKNTLTFIGQANAAGRMDEVRTLQENLEPLWDEYFQQTEGSGGSPLAQQIEYGEVLID
QARAAGRMDEVRRLSENTLQLMKEYFQQSD

His$_6$-MID1sc9_4xE (pQE vector)

His$_6$ tag D/E mutation position MID1sc9_4xE

ATGAGAGGATCGCATCACCATCACCATCACGGATCTGGAATGGGTCCGCTGGCGCAGCAGATTA
AAAACACTCTGACTTTTATTGGTCAGGCGAATGCGGCGGGCCGTATGGATGAAGTGCGTACCCT
GCAGGAAAACCTGGAACCGCTGTGGGAAGAATATTTTCAACAGACTGAAGGCTCTGGTGGTTCC
CCTTTAGCCCAACAAATCGAGTATGGCGAAGTTCTCATCGAACAAGCCAGAGCCGCCGGTCGCA
TGGACGAGGTTCGCCGTTTATCGGAGAATACGCTCCAATTAATGAAAGAGTACTTCCAACAATCC
GACTAA

MW: 12250 Da $\varepsilon_{280\ nm}$: 9970 $M^{-1}\cdot cm^{-1}$

MRGSHHHHHHGSGMGPLAQQIKNTLTFIGQANAAGRMDEVRTLQENLEPLWEEYFQQTEGSGGSP
LAQQIEYGEVLIEQARAAGRMDEVRRLSENTLQLMKEYFQQSD

His$_6$-MID1sc9_H1_EEED (pQE vector)

His$_6$ tag D/E mutation position MID1sc9_H1_EEED **DA0**

ATGAGAGGATCGCATCACCATCACCATCACGGATCTGGAATGGGTCCGCTGGCGCAGCAGATTA
AAAACATTCTGTCTTTTATTGGTCAGGCGAAAGCGGCGGGCCGTATGGATGAAGTGCGTACCCTG
CAGCTGAACCTGGAACCGCTGTGGGAAGAATATTTTCAACAGACTGAAGGCTCTGGTGGTTCCC
CTTTAGCCCAACAAATCGAGTATGGCGAAGTTCTCATCGATCAAGCCAGAGCCGCCGGTCGCAT
GGACGAGGTTCGCCGTTTATCGGAGAATACGCTCCAATTAATGAAAGAGTACTTCCAACAATCCG
ACTAA

MW: 12232 Da $\varepsilon_{280\ nm}$: 9970 $M^{-1}\cdot cm^{-1}$

MRGSHHHHHHGSGMGPLAQQIKN**I**L**S**FIGQA**K**AAGRMDEVRTLQ**L**NLEPLWEEYFQQTEGSGGSPL
AQQIEYGEVLIDQARAAGRMDEVRRLSENTLQLMKEYFQQSD

His$_6$-MID1sc9_EEED (pQE vector)

His$_6$ tag D/E mutation position MID1sc9_EEED

ATGAGAGGATCGCATCACCATCACCATCACGGATCTGGAATGGGTCCGCTGGCGCAGCAGATTA
AAAACACTCTGACTTTTATTGGTCAGGCGAATGCGGCGGGCCGTATGGATGAAGTGCGTACCCT
GCAGGAAAACCTGGAACCGCTGTGGGAAGAATATTTTCAACAGACTGAAGGCTCTGGTGGTTCC
CCTTTAGCCCAACAAATCGAGTATGGCGAAGTTCTCATCGATCAAGCCAGAGCCGCCGGTCGCA
TGGACGAGGTTCGCCGTTTATCGGAGAATACGCTCCAATTAATGAAAGAGTACTTCCAACAATCC
GACTAA

MW: 12236 Da $\varepsilon_{280\ nm}$: 9970 $M^{-1}\cdot cm^{-1}$

MRGSHHHHHHGSGMGPLAQQIKNTLTFIGQANAAGRMDEVRTLQENLEPLWEEYFQQTEGSGGSP
LAQQIEYGEVLIDQARAAGRMDEVRRLSENTLQLMKEYFQQSD

His$_6$-MID1sc9_EDED (pQE vector)

His$_6$ tag D/E mutation position MID1sc9_EDED

ATGAGAGGATCGCATCACCATCACCATCACGGATCTGGAATGGGTCCGCTGGCGCAGCAGATTA
AAAACACTCTGACTTTTATTGGTCAGGCGAATGCGGCGGGCCGTATGGATGAAGTGCGTACCCT
GCAGGAAAACCTGGAACCGCTGTGGGATGAATATTTTCAACAGACTGAAGGCTCTGGTGGTTCC
CCTTTAGCCCAACAAATCGAGTATGGCGAAGTTCTCATCGATCAAGCCAGAGCCGCCGGTCGCA
TGGACGAGGTTCGCCGTTTATCGGAGAATACGCTCCAATTAATGAAAGAGTACTTCCAACAATCC
GACTAA

MW: 12222 Da $\varepsilon_{280\ nm}$: 9970 $M^{-1}\cdot cm^{-1}$

MRGSHHHHHHGSGMGPLAQQIKNTLTFIGQANAAGRMDEVRTLQENLEPLWDEYFQQTEGSGGSP
LAQQIEYGEVLIDQARAAGRMDEVRRLSENTLQLMKEYFQQSD

His$_6$-MBP-[TEV]-LanM (pQE vector)

His$_6$ tag MBP tag TEV protease cleavage site LanM

ATGAGAGGATCGCATCACCATCACCATCACGGATCTAAAATCGAAGAAGGTAAACTGGTAATCTG
GATTAACGGCGATAAAGGCTATAACGGCCTCGCTGAAGTCGGTAAGAAATTCGAGAAAGATACCG
GAATTAAAGTCACCGTTGAGCATCCGGATAAACTGGAAGAGAAATTCCCACAGGTTGCGGCAACT
GGCGATGGCCCTGACATTATCTTCTGGGCACACGACCGCTTTGGTGGCTACGCTCAATCTGGCC
TGTTGGCTGAAATCACCCCGGACAAAGCGTTCCAGGACAAGCTGTATCCGTTTACCTGGGATGC
CGTACGTTACAACGGCAAGCTGATTGCTTACCCGATCGCTGTTGAAGCGTTATCGCTGATTTATA
ACAAAGACCTGCTGCCGAACCCGCCAAAAACCTGGGAAGAGATCCCGGCGCTGGATAAAGAACT
GAAAGCGAAAGGTAAGAGCGCGCTGATGTTCAACCTGCAAGAACCGTACTTCACCTGGCCGCTG
ATTGCTGCTGACGGGGGTTATGCGTTCAAGTATGAAAACGGCAAGTACGACATTAAAGACGTGG
GCGTGGATAACGCTGGCGCGAAAGCGGGTCTGACCTTCCTGGTTGACCTGATTAAAAACAAACA
CATGAATGCAGACACCGATTACTCCATCGCAGAAGCTGCCTTTAATAAAGGCGAAACAGCGATGA
CCATCAACGGCCCGTGGGCATGGTCCAACATCGACACCAGCAAAGTGAATTATGGTGTAACGGT
ACTGCCGACCTTCAAGGGTCAACCATCCAAACCGTTCGTTGGCGTGCTGAGCGCAGGTATTAAC
GCCGCCAGTCCGAACAAAGAGCTGGCAAAAGAGTTCCTCGAAAACTATCTGCTGACTGATGAAG
GTCTGGAAGCGGTTAATAAAGACAAACCGCTGGGTGCCGTAGCGCTGAAGTCTTACGAGGAAGA
GTTGGCGAAAGATCCACGTATTGCCGCCACTATGGAAAACGCCCAGAAAGGTGAAATCATGCCG
AACATCCCGCAGATGTCCGCTTTCTGGTATGCCGTGCGTACTGCGGTGATCAACGCCGCCAGCG
GTCGTCAGACTGTCGATGAAGCCCTGAAAGACGCGCAGACTAATTCGAGCTCGAACAACAACAA
CAATAACAATAACAACAACCTCGGGATCGAGGGAACGACCGAAAACCTGTATTTTCAGGGAGGAA
TGCCGACGACCACCACTAAAGTAGATATCGCGGCTTTTGATCCCGATAAAGACGGTACCATTGAT
CTTAAAGAGGCCTTGGCGGCAGGATCAGCGGCCTTTGATAAACTTGATCCAGATAAAGACGGTAC
TCTGGATGCCAAGGAACTGAAAGGCCGTGTTTCTGAAGCTGATCTGAAGAAGCTGGACCCCGAT
AATGACGGCACCTTAGATAAGAAAGAGTATCTGGCGGCTGTTGAGGCTCAGTTTAAAGCCGCCAA
TCCTGATAATGATGGTACCATTGATGCCCGCGAACTCGCATCGCCGGCGGGCTCTGCACTGGTC
AACCTGATCCGCTAG

MW: 56503 Da $\varepsilon_{280\ nm}$: 69330 $M^{-1}\cdot cm^{-1}$

MRGSHHHHHHGSKIEEGKLVIWINGDKGYNGLAEVGKKFEKDTGIKVTVEHPDKLEEKFPQVAATGD
GPDIIFWAHDRFGGYAQSGLLAEITPDKAFQDKLYPFTWDAVRYNGKLIAYPIAVEALSLIYNKDLLPNP
PKTWEEIPALDKELKAKGKSALMFNLQEPYFTWPLIAADGGYAFKYENGKYDIKDVGVDNAGAKAGLT
FLVDLIKNKHMNADTDYSIAEAAFNKGETAMTINGPWAWSNIDTSKVNYGVTVLPTFKGQPSKPFVGV
LSAGINAASPNKELAKEFLENYLLTDEGLEAVNKDKPLGAVALKSYEEELAKDPRIAATMENAQKGEIM
PNIPQMSAFWYAVRTAVINAASGRQTVDEALKDAQTNSSSNNNNNNNNNNLGIEGTTENLYFQGGM
PTTTTKVDIAAFDPDKDGTIDLKEALAAGSAAFDKLDPDKDGTLDAKELKGRVSEADLKKLDPDNDGTL
DKKEYLAAVEAQFKAANPDNDGTIDARELASPAGSALVNLIR

His$_6$-MBP-[TEV]-LanM_Trp (pQE vector)

His$_6$ tag MBP tag TEV protease cleavage site Mutation position LanM

ATGAGAGGATCGCATCACCATCACCATCACGGATCTAAAATCGAAGAAGGTAAACTGGTAATCTG
GATTAACGGCGATAAAGGCTATAACGGCCTCGCTGAAGTCGGTAAGAAATTCGAGAAAGATACCG
GAATTAAAGTCACCGTTGAGCATCCGGATAAACTGGAAGAGAAATTCCCACAGGTTGCGGCAACT
GGCGATGGCCCTGACATTATCTTCTGGGCACACGACCGCTTTGGTGGCTACGCTCAATCTGGCC
TGTTGGCTGAAATCACCCCGGACAAAGCGTTCCAGGACAAGCTGTATCCGTTTACCTGGGATGC
CGTACGTTACAACGGCAAGCTGATTGCTTACCCGATCGCTGTTGAAGCGTTATCGCTGATTTATA
ACAAAGACCTGCTGCCGAACCCGCCAAAAACCTGGGAAGAGATCCCGGCGCTGGATAAAGAACT
GAAAGCGAAAGGTAAGAGCGCGCTGATGTTCAACCTGCAAGAACCGTACTTCACCTGGCCGCTG

ATTGCTGCTGACGGGGGTTATGCGTTCAAGTATGAAAACGGCAAGTACGACATTAAAGACGTGG
GCGTGGATAACGCTGGCGCGAAAGCGGGTCTGACCTTCCTGGTTGACCTGATTAAAAACAAACA
CATGAATGCAGACACCGATTACTCCATCGCAGAAGCTGCCTTTAATAAAGGCGAAACAGCGATGA
CCATCAACGGCCCGTGGGCATGGTCCAACATCGACACCAGCAAAGTGAATTATGGTGTAACGGT
ACTGCCGACCTTCAAGGGTCAACCATCCAAACCGTTCGTTGGCGTGCTGAGCGCAGGTATTAAC
GCCGCCAGTCCGAACAAAGAGCTGGCAAAAGAGTTCCTCGAAAACTATCTGCTGACTGATGAAG
GTCTGGAAGCGGTTAATAAAGACAAACCGCTGGGTGCCGTAGCGCTGAAGTCTTACGAGGAAGA
GTTGGCGAAAGATCCACGTATTGCCGCCACTATGGAAAACGCCCAGAAAGGTGAAATCATGCCG
AACATCCCGCAGATGTCCGCTTTCTGGTATGCCGTGCGTACTGCGGTGATCAACGCCGCCAGCG
GTCGTCAGACTGTCGATGAAGCCCTGAAAGACGCGCAGACTAATTCGAGCTCGAACAACAACAA
CAATAACAATAACAACAACCTCGGGATCGAGGGAACGACCGAAAACCTGTATTTTCAGGGAGGAA
TGCCGACGACCACCACTAAAGTAGATATCGCGGCTTTTGATCCCGATAAAGACGGTTGGATTGAT
CTTAAAGAGGCCTTGGCGGCAGGATCAGCGGCCTTTGATAAACTTGATCCAGATAAAGACGGTAC
TCTGGATGCCAAGGAACTGAAAGGCCGTGTTTCTGAAGCTGATCTGAAGAAGCTGGACCCCGAT
AATGACGGCACCTTAGATAAGAAAGAGTATCTGGCGGCTGTTGAGGCTCAGTTTAAAGCCGCCAA
TCCTGATAATGATGGTACCATTGATGCCCGCGAACTCGCATCGCCGGCGGGCTCTGCACTGGTC
AACCTGATCCGCTAG

MW: 56589 Da $\varepsilon_{280\ nm}$: 74830 $M^{-1}\cdot cm^{-1}$

MRGSHHHHHHGSKIEEGKLVIWINGDKGYNGLAEVGKKFEKDTGIKVTVEHPDKLEEKFPQVAATGD
GPDIIFWAHDRFGGYAQSGLLAEITPDKAFQDKLYPFTWDAVRYNGKLIAYPIAVEALSLIYNKDLLPNP
PKTWEEIPALDKELKAKGKSALMFNLQEPYFTWPLIAADGGYAFKYENGKYDIKDVGVDNAGAKAGLT
FLVDLIKNKHMNADTDYSIAEAAFNKGETAMTINGPWAWSNIDTSKVNYGVTVLPTFKGQPSKPFVGV
LSAGINAASPNKELAKEFLENYLLTDEGLEAVNKDKPLGAVALKSYEEELAKDPRIAATMENAQKGEIM
PNIPQMSAFWYAVRTAVINAASGRQTVDEALKDAQTNSSSNNNNNNNNNNLGIEGTTENLYFQGGM
PTTTTKVDIAAFDPDKDGWIDLKEALAAGSAAFDKLDPDKDGTLDAKELKGRVSEADLKKLDPDNDGT
LDKKEYLAAVEAQFKAANPDNDGTIDARELASPAGSALVNLIR

His_6-MBP-[TEV]-TrpZip2 (pQE vector)

His_6 tag MBP tag TEV protease cleavage site Mutation position TrpZip2

ATGAGAGGATCGCATCACCATCACCATCACGGATCTAAAATCGAAGAAGGTAAACTGGTAATCTG
GATTAACGGCGATAAAGGCTATAACGGTCTCGCTGAAGTCGGTAAGAAATTCGAGAAAGATACCG
GAATTAAAGTCACCGTTGAGCATCCGGATAAACTGGAAGAGAAATTCCCACAGGTTGCGGCAACT
GGCGATGGCCCTGACATTATCTTCTGGGCACACGACCGCTTTGGTGGCTACGCTCAATCTGGCC
TGTTGGCTGAAATCACCCCGGACAAAGCGTTCCAGGACAAGCTGTATCCGTTTACCTGGGATGC
CGTACGTTACAACGGCAAGCTGATTGCTTACCCGATCGCTGTTGAAGCGTTATCGCTGATTTATA
ACAAAGACCTGCTGCCGAACCCGCCAAAAACCTGGGAAGAGATCCCGGCGCTGGATAAAGAACT
GAAAGCGAAAGGTAAGAGCGCGCTGATGTTCAACCTGCAAGAACCGTACTTCACCTGGCCGCTG
ATTGCTGCTGACGGGGGTTATGCGTTCAAGTATGAAAACGGCAAGTACGACATTAAAGACGTGG
GCGTGGATAACGCTGGCGCGAAAGCGGGTCTGACCTTCCTGGTTGACCTGATTAAAAACAAACA
CATGAATGCAGACACCGATTACTCCATCGCAGAAGCTGCCTTTAATAAAGGCGAAACAGCGATGA
CCATCAACGGCCCGTGGGCATGGTCCAACATCGACACCAGCAAAGTGAATTATGGTGTAACGGT
ACTGCCGACCTTCAAGGGTCAACCATCCAAACCGTTCGTTGGCGTGCTGAGCGCAGGTATTAAC
GCCGCCAGTCCGAACAAAGAGCTGGCAAAAGAGTTCCTCGAAAACTATCTGCTGACTGATGAAG
GTCTGGAAGCGGTTAATAAAGACAAACCGCTGGGTGCCGTAGCGCTGAAGTCTTACGAGGAAGA
GTTGGCGAAAGATCCACGTATTGCCGCCACTATGGAAAACGCCCAGAAAGGTGAAATCATGCCG
AACATCCCGCAGATGTCCGCTTTCTGGTATGCCGTGCGTACTGCGGTGATCAACGCCGCCAGCG
GTCGTCAGACTGTCGATGAAGCCCTGAAAGACGCGCAGACTAATTCGAGCTCGAACAACAACAA

CAATAACAATAACAACAACCTCGGGATCGAGGGAACGACCGAAAACCTGTATTTTCAGGGATCCA
GCTGGACCTGGGAAAACGGCAAATGGACCTGGAAATAA

MW: 46325 Da $\varepsilon_{280\ nm}$: 89840 $M^{-1}\cdot cm^{-1}$

MRGSHHHHHHGSKIEEGKLVIWINGDKGYNGLAEVGKKFEKDTGIKVTVEHPDKLEEKFPQVAATGD
GPDIIFWAHDRFGGYAQSGLLAEITPDKAFQDKLYPFTWDAVRYNGKLIAYPIAVEALSLIYNKDLLPNP
PKTWEEIPALDKELKAKGKSALMFNLQEPYFTWPLIAADGGYAFKYENGKYDIKDVGVDNAGAKAGLT
FLVDLIKNKHMNADTDYSIAEAAFNKGETAMTINGPWAWSNIDTSKVNYGVTVLPTFKGQPSKPFVGV
LSAGINAASPNKELAKEFLENYLLTDEGLEAVNKDKPLGAVALKSYEEELAKDPRIAATMENAQKGEIM
PNIPQMSAFWYAVRTAVINAASGRQTVDEALKDAQTNSSSNNNNNNNNNNLGIEGTTENLYFQGSS
WTWENGKWTWK

His_6-MBP-[TEV]-Tz2H3 (pQE vector)

His_6 tag MBP tag TEV protease cleavage site Mutation position Tz2H3

ATGAGAGGATCGCATCACCATCACCATCACGGATCTAAAATCGAAGAAGGTAAACTGGTAATCTG
GATTAACGGCGATAAAGGCTATAACGGTCTCGCTGAAGTCGGTAAGAAATTCGAGAAAGATACCG
GAATTAAAGTCACCGTTGAGCATCCGGATAAACTGGAAGAGAAATTCCCACAGGTTGCGGCAACT
GGCGATGGCCCTGACATTATCTTCTGGGCACACGACCGCTTTGGTGGCTACGCTCAATCTGGCC
TGTTGGCTGAAATCACCCCGGACAAAGCGTTCCAGGACAAGCTGTATCCGTTTACCTGGGATGC
CGTACGTTACAACGGCAAGCTGATTGCTTACCCGATCGCTGTTGAAGCGTTATCGCTGATTTATA
ACAAAGACCTGCTGCCGAACCCGCCAAAAACCTGGGAAGAGATCCCGGCGCTGGATAAAGAACT
GAAAGCGAAAGGTAAGAGCGCGCTGATGTTCAACCTGCAAGAACCGTACTTCACCTGGCCGCTG
ATTGCTGCTGACGGGGGTTATGCGTTCAAGTATGAAAACGGCAAGTACGACATTAAAGACGTGG
GCGTGGATAACGCTGGCGCGAAAGCGGGTCTGACCTTCCTGGTTGACCTGATTAAAAACAAACA
CATGAATGCAGACACCGATTACTCCATCGCAGAAGCTGCCTTTAATAAAGGCGAAACAGCGATGA
CCATCAACGGCCCGTGGGCATGGTCCAACATCGACACCAGCAAAGTGAATTATGGTGTAACGGT
ACTGCCGACCTTCAAGGGTCAACCATCCAAACCGTTCGTTGGCGTGCTGAGCGCAGGTATTAAC
GCCGCCAGTCCGAACAAAGAGCTGGCAAAAGAGTTCCTCGAAAACTATCTGCTGACTGATGAAG
GTCTGGAAGCGGTTAATAAAGACAAACCGCTGGGTGCCGTAGCGCTGAAGTCTTACGAGGAAGA
GTTGGCGAAAGATCCACGTATTGCCGCCACTATGGAAAACGCCCAGAAAGGTGAAATCATGCCG
AACATCCCGCAGATGTCCGCTTTCTGGTATGCCGTGCGTACTGCGGTGATCAACGCCGCCAGCG
GTCGTCAGACTGTCGATGAAGCCCTGAAAGACGCGCAGACTAATTCGAGCTCGAACAACAACAA
CAATAACAATAACAACAACCTCGGGATCGAGGGAACGACCGAAAACCTGTATTTTCAGGGATCCA
GCTGGCATTGGGAAAACGGCAAATGGCATTGGCATTAA

MW: 46406 Da $\varepsilon_{280\ nm}$: 89840 $M^{-1}\cdot cm^{-1}$

MRGSHHHHHHGSKIEEGKLVIWINGDKGYNGLAEVGKKFEKDTGIKVTVEHPDKLEEKFPQVAATGD
GPDIIFWAHDRFGGYAQSGLLAEITPDKAFQDKLYPFTWDAVRYNGKLIAYPIAVEALSLIYNKDLLPNP
PKTWEEIPALDKELKAKGKSALMFNLQEPYFTWPLIAADGGYAFKYENGKYDIKDVGVDNAGAKAGLT
FLVDLIKNKHMNADTDYSIAEAAFNKGETAMTINGPWAWSNIDTSKVNYGVTVLPTFKGQPSKPFVGV
LSAGINAASPNKELAKEFLENYLLTDEGLEAVNKDKPLGAVALKSYEEELAKDPRIAATMENAQKGEIM
PNIPQMSAFWYAVRTAVINAASGRQTVDEALKDAQTNSSSNNNNNNNNNNLGIEGTTENLYFQGSS
WHWENGKWHWH

His$_6$-MBP-[TEV]-LanPep1 (pQE vector)

His$_6$ tag MBP tag TEV protease cleavage site Mutation position LanPep1

ATGAGAGGATCGCATCACCATCACCATCACGGATCTAAAATCGAAGAAGGTAAACTGGTAATCTG
GATTAACGGCGATAAAGGCTATAACGGTCTCGCTGAAGTCGGTAAGAAATTCGAGAAAGATACCG
GAATTAAAGTCACCGTTGAGCATCCGGATAAACTGGAAGAGAAATTCCCACAGGTTGCGGCAACT
GGCGATGGCCCTGACATTATCTTCTGGGCACACGACCGCTTTGGTGGCTACGCTCAATCTGGCC
TGTTGGCTGAAATCACCCCGGACAAAGCGTTCCAGGACAAGCTGTATCCGTTTACCTGGGATGC
CGTACGTTACAACGGCAAGCTGATTGCTTACCCGATCGCTGTTGAAGCGTTATCGCTGATTTATA
ACAAAGACCTGCTGCCGAACCCGCCAAAAACCTGGGAAGAGATCCCGGCGCTGGATAAAGAACT
GAAAGCGAAAGGTAAGAGCGCGCTGATGTTCAACCTGCAAGAACCGTACTTCACCTGGCCGCTG
ATTGCTGCTGACGGGGGTTATGCGTTCAAGTATGAAAACGGCAAGTACGACATTAAAGACGTGG
GCGTGGATAACGCTGGCGCGAAAGCGGGTCTGACCTTCCTGGTTGACCTGATTAAAAACAAACA
CATGAATGCAGACACCGATTACTCCATCGCAGAAGCTGCCTTTAATAAAGGCGAAACAGCGATGA
CCATCAACGGCCCGTGGGCATGGTCCAACATCGACACCAGCAAAGTGAATTATGGTGTAACGGT
ACTGCCGACCTTCAAGGGTCAACCATCCAAACCGTTCGTTGGCGTGCTGAGCGCAGGTATTAAC
GCCGCCAGTCCGAACAAAGAGCTGGCAAAAGAGTTCCTCGAAAACTATCTGCTGACTGATGAAG
GTCTGGAAGCGGTTAATAAAGACAAACCGCTGGGTGCCGTAGCGCTGAAGTCTTACGAGGAAGA
GTTGGCGAAAGATCCACGTATTGCCGCCACTATGGAAAACGCCCAGAAAGGTGAAATCATGCCG
AACATCCCGCAGATGTCCGCTTTCTGGTATGCCGTGCGTACTGCGGTGATCAACGCCGCCAGCG
GTCGTCAGACTGTCGATGAAGCCCTGAAAGACGCGCAGACTAATTCGAGCTCGAACAACAACAA
CAATAACAATAACAACAACCTCGGGATCGAGGGAACGACCGAAAACCTGTATTTTCAGGGATCCA
GCTGGGATTGGGAAAACGGCAAATGGGATTGGGATTAA

MW: 46340 Da $\varepsilon_{280\ nm}$: 89840 $M^{-1}\cdot cm^{-1}$

MRGSHHHHHHGSKIEEGKLVIWINGDKGYNGLAEVGKKFEKDTGIKVTVEHPDKLEEKFPQVAATGD
GPDIIFWAHDRFGGYAQSGLLAEITPDKAFQDKLYPFTWDAVRYNGKLIAYPIAVEALSLIYNKDLLPNP
PKTWEEIPALDKELKAKGKSALMFNLQEPYFTWPLIAADGGYAFKYENGKYDIKDVGVDNAGAKAGLT
FLVDLIKNKHMNADTDYSIAEAAFNKGETAMTINGPWAWSNIDTSKVNYGVTVLPTFKGQPSKPFVGV
LSAGINAASPNKELAKEFLENYLLTDEGLEAVNKDKPLGAVALKSYEEELAKDPRIAATMENAQKGEIM
PNIPQMSAFWYAVRTAVINAASGRQTVDEALKDAQTNSSSNNNNNNNNNLGIEGTTENLYFQGSS
WDWENGKWDWD

His$_6$-MBP-[TEV]-LanPep2 (pQE vector)

His$_6$ tag MBP tag TEV protease cleavage site Mutation position LanPep2

ATGAGAGGATCGCATCACCATCACCATCACGGATCTAAAATCGAAGAAGGTAAACTGGTAATCTG
GATTAACGGCGATAAAGGCTATAACGGTCTCGCTGAAGTCGGTAAGAAATTCGAGAAAGATACCG
GAATTAAAGTCACCGTTGAGCATCCGGATAAACTGGAAGAGAAATTCCCACAGGTTGCGGCAACT
GGCGATGGCCCTGACATTATCTTCTGGGCACACGACCGCTTTGGTGGCTACGCTCAATCTGGCC
TGTTGGCTGAAATCACCCCGGACAAAGCGTTCCAGGACAAGCTGTATCCGTTTACCTGGGATGC
CGTACGTTACAACGGCAAGCTGATTGCTTACCCGATCGCTGTTGAAGCGTTATCGCTGATTTATA
ACAAAGACCTGCTGCCGAACCCGCCAAAAACCTGGGAAGAGATCCCGGCGCTGGATAAAGAACT
GAAAGCGAAAGGTAAGAGCGCGCTGATGTTCAACCTGCAAGAACCGTACTTCACCTGGCCGCTG
ATTGCTGCTGACGGGGGTTATGCGTTCAAGTATGAAAACGGCAAGTACGACATTAAAGACGTGG
GCGTGGATAACGCTGGCGCGAAAGCGGGTCTGACCTTCCTGGTTGACCTGATTAAAAACAAACA
CATGAATGCAGACACCGATTACTCCATCGCAGAAGCTGCCTTTAATAAAGGCGAAACAGCGATGA
CCATCAACGGCCCGTGGGCATGGTCCAACATCGACACCAGCAAAGTGAATTATGGTGTAACGGT
ACTGCCGACCTTCAAGGGTCAACCATCCAAACCGTTCGTTGGCGTGCTGAGCGCAGGTATTAAC
GCCGCCAGTCCGAACAAAGAGCTGGCAAAAGAGTTCCTCGAAAACTATCTGCTGACTGATGAAG

GTCTGGAAGCGGTTAATAAAGACAAACCGCTGGGTGCCGTAGCGCTGAAGTCTTACGAGGAAGA
GTTGGCGAAAGATCCACGTATTGCCGCCACTATGGAAAACGCCCAGAAAGGTGAAATCATGCCG
AACATCCCGCAGATGTCCGCTTTCTGGTATGCCGTGCGTACTGCGGTGATCAACGCCGCCAGCG
GTCGTCAGACTGTCGATGAAGCCCTGAAAGACGCGCAGACTAATTCGAGCTCGAACAACAACAA
CAATAACAATAACAACAACCTCGGGATCGAGGGAACGACCGAAAACCTGTATTTTCAGGGATCCA
GCTGGGAATGGGAAAACGGCAAATGGGAATGGGAATAA

MW: 46382 Da $\varepsilon_{280\ nm}$: 89840 $M^{-1} \cdot cm^{-1}$

MRGSHHHHHHGSKIEEGKLVIWINGDKGYNGLAEVGKKFEKDTGIKVTVEHPDKLEEKFPQVAATGD
GPDIIFWAHDRFGGYAQSGLLAEITPDKAFQDKLYPFTWDAVRYNGKLIAYPIAVEALSLIYNKDLLPNP
PKTWEEIPALDKELKAKGKSALMFNLQEPYFTWPLIAADGGYAFKYENGKYDIKDVGVDNAGAKAGLT
FLVDLIKNKHMNADTDYSIAEAAFNKGETAMTINGPWAWSNIDTSKVNYGVTVLPTFKGQPSKPFVGV
LSAGINAASPNKELAKEFLENYLLTDEGLEAVNKDKPLGAVALKSYEEELAKDPRIAATMENAQKGEIM
PNIPQMSAFWYAVRTAVINAASGRQTVDEALKDAQTNSSSNNNNNNNNNNLGIEGTTENLYFQGSS
WEWENGKWEWE

Recombinant expression and protein purification

For recombinant expression, a 50 µL aliquot of *E. coli* BL21(DE3) GOLD was transformed with 1 µL plasmid solution (50 – 150 ng) via heat shock. The inoculum was transferred into 20 mL LB medium (supplemented with 100 $\mu g \cdot mL^{-1}$ ampicillin) and propagated at 37°C and 180 rpm overnight. For the main culture, 0.5 – 1.5 L TB medium (supplemented with appropriate antibiotic) was inoculated with preculture to an OD_{600} of 0.05 and incubated for 3 – 3.5 h at 37°C and 180 rpm (OD_{600} 0.8-1.2). Gene expression was induced by addition of isopropyl-β-D-thiogalactopyranosid (IPTG, final concentration 0.5 mM) and continued the incubation at 18°C and 180 rpm for at least 16 h. Cells were harvested by centrifugation (4347xg, 4°C, 45 min) washed once with bidestilled H_2O and was stored at -20°C until usage.

Protein purification was conducted by resuspending the cell pellet in lysis buffer (25 mM HEPES, 300 mM NaCl, 20 mM imidazole, 1 $mg \cdot mL^{-1}$ lysozyme, 0.1 $mg \cdot mL^{-1}$ DNaseI, 1 mM $MgCl_2$, pH 7.5) using 7 mL volume per g bio wet mass. After 30 min incubation on ice, the cells were disrupted by sonication using Branson SFX 500 sonifier (*Emerson Electric Co.*, St. Louis, MO, USA; power-on time: 10-12 min, pulsed time: 5 s on, 7 s off, 35% power). The lysate was centrifuged (11000xg, 4°C, 30 min) and the cell-free extract was applied on pre-equilibrated Protino® Ni-NTA agarose resin (*Marchery-Nagel*, Düren, Germany), filled in gravity-flow column (*Bio-Rad*, Feldkirchen, Germany; column volume (CV): 5 or 10 mL). The flow-through was discarded and the resin was washed with 7 CV of wash buffer (25 mM HEPES, 300 mM NaCl, 20 mM imidazole, pH 7.5). The protein was eluted with 3 CV elution buffer (25 mM HEPES, 300 mM NaCl, 300 mM imidazole, pH 7.5).

Fusion proteins comprising a TEV cleavage site were treated with His_6-TEV protease (approx. 0.2 mg per mg target protein), transferred into SERVAPOR® 3 dialysis tubing (*SERVA Electrophoresis GmbH*, Heidelberg, Germany; 3.5 kDa cut-off) and dialysed overnight at 4°C in 4 L dialysis buffer (25 mM HEPES, 300 mM NaCl, pH 7.5) containing Chelex® 100 resin. Afterwards, a subtractive IMAC was performed to remove TEV protease, cleaved tag and non-cleaved protein. The dialysate was applied on pre-equilibrated Ni-NTA resin and washed with 5 CV wash buffer. Bound proteins were eluted with 3 CV elution buffer. Flow-through and wash were collected and concentrated by ultrafiltration using Amicon© Ultra centrifugal filter units (*Merck KGaA*, Darmstadt, Germany; 3 kDa cut-off, 4200xg, 4°C) to a final volume of 2-5 mL.

The concentrated protein solution was passed through a syringe filter (0.45 µm) and subjected to size-exclusion chromatography (SEC) on a NGC Quest 10 Plus Chromatography system (*Bio-Rad Laboratories GmbH*, Feldkirchen, Germany), equipped with a multi-wavelength UV/Vis detector (set wavelengths λ_1: 260 nm, λ_2: 280 nm), conductivity detector, a Superdex 75 Increase 10/300 GL and a HiLoad 16/600 Superdex 75 pg column (*Cytiva*, Marlborough, MA, USA). The purification was conducted either at room temperature (25-28°C) or at 6°C. Columns were pre-equilibrated with SEC buffer (25 mM HEPES, 100 mM NaCl, pH 7.5). Following methods were applied: 1) Superdex 75 Increase 10/300 GL: 1-2 mL protein solution was applied, flow rate: 0.65 $mL \cdot min^{-1}$, equilibration volume: 2 mL, elution volume: 26 mL, fraction volume: 0.5 mL; 2) HiLoad 16/600 Superdex 75 pg: 5 mL protein solution was applied, equilibration volume: 5 mL, flow rate: 1.0 $mL \cdot min^{-1}$, elution volume: 129 mL, fraction volume: 1.0 mL. Fractions were analyzed by SDS-PAGE and those containing the protein of interest were pooled and concentrated using Amicon© Ultracentrifugal filter units (*Merck KGaA*, Darmstadt, Germany; 3 kDa cut-off, 4200xg, 4°C). The concentrated protein solutions were frozen in liquid N_2 and stored at -70°C. Protein concentrations were determined spectroscopically at 280 nm using calculated ε_{280nm} values.

Plate-based screening assay for Tb^{3+} binding

General procedure: Tb^{3+} binding screening was carried out on commercial HIS Select© Filter plates with following procedure: 1) Equilibration of HIS Select® filter plates with 600 µL per well wash buffer (25 mM HEPES, 300 mM NaCl, 5 mM imidazole); 2) 800 µL per well cell-free lysate was applied; 3) 600 µL per well wash buffer was applied, repeated 2x; 4) 600 µL per well Tb^{3+} loading buffer (25 mM HEPES, 300 mM NaCl, pH 7.5, 100 µM $TbCl_3$) was added to the pleated, centrifuge and incubated for 10-15 min at 4°C after centrifugation; 5) 800 µL per well imidazole-free wash buffer (25 mM HEPES, 300 mM NaCl, pH 7.5) was applied, 6x repeated; 6) Protein was eluted with 500 µL per well elution buffer (25 mM HEPES, 300 mM NaCl, 300 mM imidazole, pH 7.5). The filter plates were centrifuged (2000xg, 6°C, 2 min), if not stated otherwise.

Tb^{3+} binding was evaluated on a Varioskan LUX microplate reader (*Thermo Fisher Scientific*). 198 µL per well of the eluted fraction was transferred in 96-well Nunc™ MicroWell™ microplate (black, F-bottom; *Thermo Fisher Scientific*) and 2 µL per well of 10 mM 2,3-Dihydroxynaphtahlene (2,3-DHN, final concentration: 100 µM, in 25 mM

HEPES, 300 mM NaCl, pH 7.5) was added. The microtiter plate was shaken prior to the measurement (600 rpm, 5s, orbital mode). Tb^{3+} luminescence was measured using the time-resolved fluorescence mode (TRF, 200 µs delay time, 1 ms integration time, 100 ms measurement time; excitation wavelength: 280 ± 12 nm (Trp) and 324 ± 12 nm (2,3-DHN), excitation wavelength: 544 - 546 ± 1 nm).

For the assay validation, the Trp-sensitized Tb^{3+} luminescence was measured before and after addition of 2,3-DHN for all control proteins and the MBP-peptide fusions.

To remove any Tb^{3+} ions bound to the resin, the filter plates had to be regenerated after each usage with following protocol: 1) 600 µL per well bidestilled H_2O, 2) 500 µL per well stripping buffer (50 mM Na_2HPO_4, 300 mM NaCl, 100 mM EDTA, pH 8.0), 3) 800 µL per well bidestilled H_2O, 5x repeats, 4) 500 µL per well 100 mM $NiCl_2$ solution was added, centrifuged and incubated for 30-60 min room temperature, 5) 800 µL per well bidestilled H_2O was added, 5x repeats, 6) 800 µL per well wash buffer (25 mM HEPES, 300 mM NaCl, 5 mM imidazole, pH 7.5) was added, 3x repeats. The filter plates were stored at 4°C until usage.

10 mM 2,3-DHN stocks were prepared by mixing the solids with an appropriate volume of degassed buffer (25 mM HEPES, 300 mM NaCl, pH 7.5) in 2-mL Eppendorf tubes and dissolving it on a Thermoshaker (55°C, 1200 rpm, 5-10 min). The solution was centrifuged (13000xg, 5 min) and the supernatant was split into 1-mL aliquots. For long-term storage, the aliquots were frozen in liquid N_2 and stored at -70°C.

<u>Initial tests and assay validation with control proteins (see Figure S4):</u> Plasmids encoding for the different proteins (His_6-MBP-TEV-sfGFP, His_6-TEV-sfGFP, His_6-sfGFP, His_6-TEV-MID1sc9, PedH-His_6, PedH_2xMut-His_6, His_6-MBP-TEV-LanM, His_6-MBP-TEV-LanM_Trp) were transformed in *E. coli* BL21(DE3) GOLD and expression was conducted as described before in 500 mL culture volume. Cells were harvested by centrifugation and resuspended in lysis buffer (25 mM HEPES, 300 mM NaCl, 5 mM imidazole, 1 mg·mL^{-1} lysozyme, 0.1 mg·mL^{-1} DNaseI, 1 mM $MgCl_2$, pH 7.5) using 7 mL volume per g bio wet mass, following sonification. The cell-free extract was subjected onto pre-equilibrated HIS Select® filter plates, following the described procedure. The concentration of eluted protein was determined spectroscopically at 280 nm on NanoDrop One (*Thermo Fisher Scientific*) using calculated ε_{280nm} values. The average from 4-8 wells was determined, and standard deviation error was

reported. Protein purification was monitored by SDS-PAGE with samples withdrawn at different stages of the purification.

Screening after expression in deep-well plates: In general, plasmids which harbor the encoding genes (PedH-His_6, PedH_2xMut-His_6, His_6-MBP tagged peptides TrpZip2, Tz2H3, LanPep1, LanPep2, MID1sc_DE library) were transformed into *E. coli* BL21(DE3) GOLD via heat-shock and spread out on selective LB agar with appropriate antibiotics. A 96-well Nunc™ MicroWell™ microplate (transparent, F-bottom; *Thermo Fisher Scientific*) was filled with 120 µL per well LB medium (supplemented with appropriate antibiotic) and inoculated with a single colony (or 10 µL inoculum from a prior master plate). The microtiter plate was sealed with a Breath-easy® membrane (*Thermo Fisher Scientific*) and incubated overnight on microplate incubator (*Ohaus Europe GmbH*, Nänikon, Switzerland; 37°C, 900 rpm). 990 µL TB medium (supplemented with appropriate antibiotic) in 96-deepwell plate (U-bottom; *Carl Roth GmbH+Co. KG*, Karlsruhe, Germany) was inoculated with 10 µL pre-culture, sealed with a BreathEasier® membrane (*Diversified Biotech*) and incubated for 3 h on a microplate incubator (37°C, 900 rpm). Gene expression was induced with 50 µL IPTG solution (10.5 mM in supplemented TB medium, final concentration: 0.5 mM) and incubation was continued (21-23°C, 1000 rpm) for at least 16 h. Plates were centrifuged (4347xg, 45 min, 4°C) and stored at -70°C until usage.

Prior to cell lysis, the plates were subjected to two freeze/thaw cycles by thawing in a water bath (30°C) with ultrasound for 10 min, followed by freezing at -70°C for at least 30 min. The pellets were resuspended in 1.0 mL per well lysis buffer (25 mM HEPES, 300 mM NaCl, 5 mM imidazole, 1.25% Triton X-100, 30 µM Polymyxine B, 1 $mg \cdot mL^{-1}$ lysozyme, 1 mM $MgCl_2$, 1 $\mu g \cdot mL^{-1}$ DNaseI, pH 7.5) and incubated for 1 h on microplate incubator (750 rpm, 37°C) with intermittent resuspension using a 96-well multichannel pipette (10x up/down, after 30 min and 60 min) to support cell disruption. The microplates were centrifuged (4347xg, 45 min, 4°C) and 800 µL per well of the cell-free extract were transferred on pre-equilibrated HIS Select® filter plates, following the screening procedure described above. The protein concentration in the elution fraction of PedH-His_6/PedH_2xMut-His_6 and the His_6-MBP tagged peptides was determined spectroscopically at 280 nm on NanoDrop One (*Thermo Fisher Scientific*) using calculated ε_{280nm} values. The average from 3 wells was determined and standard deviation error was calculated.

For the library screening of the MID1sc_DE library, five plates (~ 400 variants) were screened following this procedure. The plate layout is shown in **Figure S14A**. Following controls were included: His_6-sfGFP (not binding Tb^{3+}, expression control), His_6-MID1sc9 (parental scaffold), uninduced cells (host cell background) and sterile medium (blank background). Expression and purification were monitored by denatured SDS-PAGE of withdrawn samples (see **Figure S14B**). The detected luminescence values from excitation channels at 280 nm (Trp) and 324 nm (2,3-DHN) were multiplied and 3-5 wells with the highest combined luminescence values were subjected for a validation screening (see **Figure S14C**). In total, 20 variants were re-screened and the four hits with the highest values were selected for large scale expression and characterization.

<u>Phosphate addition to increase the assay stringency</u>: For initial testing, 10 µM of an equimolar mixture of $TbCl_3$ and purified His_6-MID1sc9_H1_EEED (weak binder) or His_6-MID1sc9_EDED (strong binder), respectively, was prepared in technical triplicates in buffered solution (25 mM HEPES, 300 mM NaCl, pH 7.5) and incubated for 1 h at room temperature prior to titration. 0 – 100 µM buffered sodium phosphate was titrated in a black Nunc™ MicroWell™ microplate and 200 µL total volume. The microtiter plate was shaken (600 rpm, 5 s, orbital mode) and Tb^{3+} luminescence was monitored (total: 0.5 h, interval: 1 min) with same TRF settings as described above.

The phosphate treatment was then implemented for full plate-based screening procedure. His_6-MID1sc9_H1_EEED and His_6-MID1sc9_EDED were expressed in *E. coli* BL21(DE3) GOLD and a uniform cell-free extract was prepared and loaded onto pre-equilibrated filter plates. Following the described procedure, a stringency wash step using phosphate wash buffer (25 mM HEPES, 300 mM NaCl, pH 7.5, 0-250 µM potassium phosphate) as depicted in **Figure 5B** was applied after the first wash with imidazole-free wash buffer. Trp and 2,3-DHN sensitized Tb^{3+} luminescence and protein concentration of the eluted fraction were determined as previously described.

Excitation scans and controls evaluating the spectral crosstalk for the terbium luminescence signals sensitized by Trp and 2,3-DHN

Equimolar solutions of PedH:Tb^{3+} (10 µM) and 2,3-DHN:Tb^{3+} (100 µM) were prepared in buffer (25 mM HEPES, 100 mM NaCl, pH 7.0), respectively, and 200 µL were transferred into a Nunc™ MicroWell™ microplate (black, F-bottom; *Thermo Fisher*

Scientific). Excitation spectra were recorded on a Varioskan LUX microplate reader (*Thermo Fisher Scientific*) in time-resolved fluorescence mode (TRF, 50 µs delay time, 1 ms integration time, 200 ms measurement time; excitation wavelength: 200 – 400 ± 5 nm, emission wavelength: 545 ± 1 nm (Tb^{3+}), T: 25°C).

To assess potential effects of the spectral overlap of both antennas at the specified excitation wavelengths, equimolar mixtures of PedH:Tb^{3+}, 2,3-DHN:Tb^{3+}, and PedH:Tb^{3+}:2,3-DHN were prepared at 10 µM concentration each in buffer. Sensitized Tb^{3+} luminescence was measured in TRF mode (50 µs delay time, 1 ms integration time, 200 ms measurement time; excitation wavelengths: 280 ± 12 nm (Trp) and 324 ± 12 nm (2,3-DHN), emission wavelength: 544 - 546 ± 1 nm (Tb^{3+}), T: 25°C).

Tb^{3+} titrations to determine binding affinities

All measurements were prepared in Nunc™ MicroWell™ microplate (black, F-bottom; *Thermo Fisher Scientific*) and recorded on a Varioskan LUX microplate reader (*Thermo Fisher Scientific*) in time-resolved fluorescence mode (TRF, 200 µs delay time, 1 ms integration time, 100 ms measurement time; excitation wavelength: 280 ± 12 nm (Trp), emission wavelength: 545 ± 1 nm (Tb^{3+}), T: 25°C). MID1sc9 variants were diluted to 0.2 µM in titration buffer (25 mM HEPES, 100 mM NaCl, pH 7.0) and titrated with 15.6 nM – 2.0 µM $TbCl_3$ (except His_6-MID1sc9_H1_EEED: 39.1 nM – 5 µM) in 200 µL total volume. Peptides were diluted to 15 µM in titration buffer and titrated with 7.3 µM – 15 mM $TbCl_3$ (LanPep1, LanPep2) and 0.15 – 15 mM $TbCl_3$ (Tz2H3), respectively. Immediately after $TbCl_3$ addition, the microtiter plate was shaken (600 rpm, 5s, orbital mode) and Tb^{3+} luminescence was monitored (t_{total}: 1 h, $t_{interval}$: 1 min). Values at 1 h were plotted and the dissociation constant K_D was determined with *GraphPad Prism 5* using quadratic equation fit:

$$F = F_0 - \frac{F_{Ampl}}{2 \cdot [P]} \cdot \left([P] + [Tb^{3+}] + K_D - \sqrt{([P] + [Tb^{3+}] + K_D)^2 - 4 \cdot [P] \cdot [Tb^{3+}]}\right)$$

F_0 is the minimal luminescence signal, F_{Ampl} is the luminescence amplitude, *[P]* is the protein/peptide concentration in µM, $[Tb^{3+}]$ is the $TbCl_3$ concentration in µM, K_D is the dissociation constant in µM.

For PedH and PedH_2xMut, samples were diluted to 0.5 µM in buffer (25 mM HEPES, 150 mM NaCl, pH 7.5) and titrated with 0 µM – 10 mM $TbCl_3$ in 100 µL total volume. The time-resolved fluorescence (TRF, 50 µs delay time, 1 ms integration time, 200 ms

measurement time; excitation wavelength: 280 ± 12 nm (Trp), emission wavelength: 545 ± 1 nm (Tb^{3+}), T: 25°C) was monitored for 100 min every 1.5 min and the Tb^{3+} affinity was determined by quadratic fit.

To assess the apparent Tb^{3+} affinity of the external antenna 2,3-DHN, samples of 10 µM 2,3-DHN in buffer (25 mM HEPES, 100 mM NaCl, pH 7.0) were prepared and titrated with 15.6 – 750 µM $TbCl_3$ in 200 µL total volume. The sensitized terbium luminescence signal (TRF mode, 200 µs delay time, 1 ms integration time, 200 ms measurement time; excitation wavelength: 324 ± 12 nm (2,3-DHN), emission wavelength: 545 ± 1 nm (Tb^{3+}), T: 25°C) was monitored for 30 min every 1 min and the apparent Tb^{3+} affinity was estimated by data fitting with the Hill equation.

Tb^{3+} displacement titrations with Ca^{2+} and other Ln^{3+} ions

To estimate the relative Ln^{3+} selectivity, a solution of 4 µM equimolar $TbCl_3$ and MID1sc9 variant was prepared in titration buffer and incubated for 1 h at room temperature. In a black 96-well microtiter plate, 50 µL of $TbCl_3$:MID1sc9 variant solution (1 µM final concentration) and 50 µL of 4 µM $LnCl_3$ (La^{3+}, Ce^{3+}, Pr^{3+}, Nd^{3+}, Sm^{3+}, Eu^{3+}, Gd^{3+}, Dy^{3+}, Ho^{3+}, Er^{3+}, Yb^{3+}; 1 µM final concentration) was combined in 200 µL total volume. For MID1sc9_H1_EEED, displacement was conducted by incubation of 1 µM protein and 5 µM $TbCl_3$, followed by addition of 5 µM of $LnCl_3$. After shaking the microtiter plate (600 rpm, 5s, orbital mode), the Tb^{3+} luminescence decrease was monitored utilizing the same settings as for Tb^{3+} titration. The luminescence values were normalized to $TbCl_3$:MID1sc9 in buffer.

The dissociation constants K_D of MID1sc9_4xE for binding Ca^{2+}, La^{3+}, Eu^{3+} and Yb^{3+} were determined by titrating 0-750 mM $CaCl_2$, 0-128 µM $LaCl_3$, 0-10 µM $EuCl_3$ or 0-20 µM $YbCl_3$ to an equilibrated solution of 1 µM MID1sc9_4xE + 1 µM $TbCl_3$. The microtiter plate was shaken (600 rpm, 5s, orbital mode) and Tb^{3+} luminescence was monitored (t_{total}: 1 h, $t_{Interval}$: 1 min). Values at 1 h were plotted and K_D values were determined using a cubic equation fit for metal ion displacement:

$$[AB]^3 + a_1[AB]^3 + a_2[AB]^3 + a_3 = 0$$

$$a_0 = K_{D_{AB}} - K_{D_{AC}}$$

$$a_1 = \frac{[A_0] \cdot (K_{D_{AC}} - K_{D_{AB}}) + [B_0] \cdot (2 \cdot K_{D_{AC}} - K_{D_{AB}}) + [C_0] \cdot K_{D_{AB}} - (K_{D_{AB}})^2 + K_{D_{AB}} \cdot K_{D_{AC}}}{[A_0]}$$

$$a_2 = \frac{[A_0]\cdot[B_0](K_{D_{AB}} - 2\cdot K_{D_{AB}}) + [B_0]^2\cdot K_{D_{AC}} - [B_0]\cdot K_{D_{AB}}\cdot([C_0]\cdot K_{D_{AB}})}{[A_0]}$$

$$a_3 = \frac{[A_0]\cdot[B_0]^2\cdot K_{D_{AC}}}{[A_0]}$$

$$Q = \frac{{a_1}^2 - 3\cdot a_2}{9}$$

$$R = \frac{2\cdot {a_1}^3 - 9\cdot a_1\cdot a_2 + 27\cdot a_3}{54}$$

$$\theta = \arccos\left(\frac{R}{\sqrt{Q}^3}\right)$$

$$[AB]_1 = -2\cdot\sqrt{Q}\cdot cos\left(\frac{\theta}{3}\right) - \frac{a_1}{3}$$

$$[AB]_2 = -2\cdot\sqrt{Q}\cdot cos\left(\frac{\theta + 2\cdot\pi}{3}\right) - \frac{a_1}{3}$$

$$[AB]_3 = -2\cdot\sqrt{Q}\cdot cos\left(\frac{\theta + 4\cdot\pi}{3}\right) - \frac{a_1}{3}$$

$$F = F_0 + F_{Ampl}\cdot\frac{[AB]_X}{[A_0]}$$

F_0 is the minimal luminescence signal, F_{Ampl} is the luminescence amplitude, $[A_0]$ is the protein concentration in µM, $[B_0]$ is the $TbCl_3$ concentration in µM, $[C_0]$ is the $LnCl_3$ concentration in µM, $K_{D,\ AB}$ is the dissociation constant of Tb^{3+} in µM (for MID1sc9_4xE: 0.0316 µM), and $K_{D,\ AC}$ is the unknown dissociation constant of Ca^{2+} or Ln^{3+} in µM. All measurements were performed in technical triplicates.

Competition measurements of protein and 2,3-DHN for Tb^{3+}

4 µM $TbCl_3$ + 4 µM protein (MID1sc9_4xE or PedH-His) were prepared in buffered solution (25 mM HEPES, 100 mM NaCl, pH 7.0) and incubated for 1 h at room temperature ([MID1sc9_4xE:Tb^{3+}]) or on ice ([PedH-His:Tb^{3+}]). In a 96-well Nunc™ MicroWell™ microplate (black, F-bottom; *Thermo Fisher Scientific*), 50 µL of protein:Tb^{3+} solution (1 µM final concentration) and 50 µL 0-1 mM 2,3-DHN solution (in the same buffer, 0-250 µM final concentration) was combined in 200 µL total volume. The plate was shaken (600 rpm, 5 s, orbital mode) and the Tb^{3+} luminescence was monitored in time-resolved fluorescence mode (TRF, 200 µs delay time, 1 ms

integration time, 200 ms measurement time; excitation wavelengths: 280 ± 12 nm (Trp) and 324 ± 12 nm (2,3-DHN), emission wavelength: 545 ± 1 nm (Tb^{3+}), T: 25°C) was monitored for 30 min every 1 min.

Circular dichroism (CD) spectroscopy

CD measurements were performed on a Jasco J-815 CD spectrometer, equipped with a Jasco PTC-423S Peltier thermostatted cell holder. The device was flushed with a stream of N_2 of 5.0 – 5.5 L·min^{-1}. Following settings were used: Scanning speed: 100 nm·min^{-1}, data pitch: 0.1 nm, sensitivity: low, D. I. T.: 2 s, bandwidth: 1.0 nm. The spectra were recorded at 20 ± 0.1 °C from 190 – 350 nm with 10 accumulations. All signals were recorded in millidegree (mdeg) and converted to mean residue molar ellipticity (MRE). Prior to CD measurements, MID1sc9 samples were dialyzed overnight in 1.0 L buffer (20 mM NH_4HCO_3, pH 8.0) using Xpress Micro Dialyzer tubes (MWCO: 3.5 kDa; *SERVA*, Heidelberg, Germany) at 4°C, followed by dilution to a final concentration of 10 µM. Peptides samples were diluted to a final concentration of 50 µM in 10 mM HEPES, 100 mM NaCl, pH 7.0. PedH and PedH-2xMut were dialyzed overnight in buffer (10 mM Tris, 150 mM NaF, pH 7.5) and diluted to 5 µM. Here, the spectra were recorded at 5°C. $LnCl_3$ containing samples were incubated for at least 1 h at room temperature before the measurement.

Thermal unfolding of proteins was measured from 20 to 95°C following a thermal ramp of 1°C·min^{-1} and data pitch every 5°C with 30 s of equilibration time. For peptides, a temperature range of 0 to 95°C was followed with a thermal ramp of 1°C·min^{-1} and data pitch every 2°C with 30 s of equilibration time. Whole spectra were recorded with aforementioned settings and 3 accumulations. The melting temperature T_M was determined by fitting the plotted MRE values at 222 nm (proteins) or 228 nm (peptides) with the sigmoidal Boltzmann equation:

$$MRE = A_0 + A \cdot \frac{1}{1 + e^{-\frac{(T-T_M)}{\Delta T}}}$$

MRE represents the measured mean residue ellipticity, A_0 is minimum value of the signal, A is the signal amplitude, T is the temperature, T_M is the melting temperature, and ΔT is the slope at the transition point. Except for peptides, T_M determinations were conducted in technical duplicates and reported as average and standard deviation errors.

Isothermal titration calorimetry (ITC)

ITC measurements were conducted on a MicroCal PEAQ-ITC® (Malvern Pananalytical, Worcestershire, UK). Prior to measurements, a sample of MID1sc9_4xE (2.42 mM, ~ 400 µL) was dialyzed overnight against 1.0 L buffer (25 mM HEPES, 100 mM NaCl, pH 7.0) using Xpress Micro Dialyzer tubes (MWCO: 3.5 kDa; *SERVA*, Heidelberg, Germany) at 4°C, followed by dilution to a final concentration of 25 µM and filled in the sample cell (V_{Sample}: 280 µL). 0.5 mM $LnCl_3$ (Ln = La, Tb, Eu, Yb) titrant solution was freshly prepared by diluting a 200 mM solution in the dialysis buffer. The following experimental settings were used: 6 $µcal·s^{-1}$ reference power, 500 rpm stir speed, 0.4 µL initial injection volume with 60 s delay time and 18x 1 µL injection volume with 150 s spacing time. The reference cell was filled with MilliQ water. Control titrations ($LnCl_3$ into buffer) were conducted with the same settings to determine the background signal. The recorded data were plotted as differential power (DP) vs. time. The data were analyzed using the manufacturer's proprietary analysis software (Version 1.41). Following subtraction of the background signal and baseline adjustment, the thermodynamic parameters were determined by curve fitting of ΔH vs. molar ratio.

Analytical size exclusion chromatography with static light scattering detection (SEC-SLS)

Static light scattering measurements were performed using a Viscotek TDA 305 triple array detector (*Malvern Instruments*) attached to an analytical size exclusion chromatography column. Prior to the analysis, the SLS detector was calibrated with a bovine serum albumin (BSA) solution at 1.9 $mg·mL^{-1}$ using 66.5 kDa for the BSA monomer and a dn/dc value of 0.185 $mL·g^{-1}$ for all protein samples. Samples were diluted to ca. 100 µM concentration in buffer (25 mM HEPES, 100 mM NaCl, pH 7.0) in the presence or absence of one equivalent of $TbCl_3$ and incubated for 1 h at 25°C prior to measurement. Analysis was performed on a Superdex 75 column at a flow rate of 0.5 mL/min and SEC buffer (25 mM HEPES 100 mM NaCl, pH 7.0). The molecular masses of the eluted species were calculated from the refractive index and right-angle light scattering signals using the Omnisec software (*Malvern Instruments*).

Mass spectrometry with peptides and proteins

All analyses were performed on an Agilent 1290 Infinity II UHPLC system (*Agilent Technologies*, Santa Clara, CA, USA) equipped with a diode array detector (G7117C)

and a XT single-quadrupole mass detector (G6135B) with an AJS (Agilent Jet Stream) electrospray ion source. Water (+ 0.1% formic acid; Solvent A) and acetonitrile (+ 0.1% formic acid; Solvent B) were used as eluents. Proteins were analyzed on a Poroshell 300SB C18 column (75 x 2.1 mm, 5 µm particle size; *Agilent Technologies*, Santa Clara, CA, USA) at 25 °C with following gradient program (flow rate of 0.8 $mL \cdot min^{-1}$): isocratic at 5% B for 1 min, gradient 5-40% B for 14 min, gradient 40-95% for 5 min, isocratic 95% B for 5 min and step gradient to 5% B in 0.1 min with 5 min post-run time for re-equilibration. Peptides were analyzed on a Poroshell 120 EC-C18 column (100 x 2.1 mm, 1.9 µm particle size; *Agilent Technologies*, Santa Clara, CA, USA) at 30 °C with following gradient program (flow rate of 0.4 $mL \cdot min^{-1}$): isocratic at 10% B for 1 min, gradient 10-50% B for 39 min, gradient 50-95% for 5 min, isocratic 95% B for 3 min, followed by 3 min post-run time for re-equilibration. Sample were diluted to 0.1 $mg \cdot mL^{-1}$ in H_2O (+ 1.0% formic acid) and 2 µL volume was injected. UV/Vis detection wavelength was set to 280 nm (proteins) and 205 nm (peptides). Mass detection was conducted in positive mode with a set range of 500 – 3000 m/z in full storage mode. Protein mass was determined by deconvolution in default settings using OpenLab CDS ChemStation Software (*Agilent Technologies*, Santa Clara, CA, USA).

Protein X-ray crystallography

<u>Protein crystallization:</u> The protein was crystallized in sitting drop vapor diffusion experiments. Droplets were pipetted by the Crystal Phoenix pipetting robot (*Art Robbins Instruments*), and initial hits were used for a seed stock. For this, the crystals were crushed, transferred in 40 µL of ice-cooled 2.9 M sodium malonate pH 4.0, and vortexed four times for 30 s each. Well diffracting crystals grew in 0.5 µL droplets consisting of 0.2 µL protein solution (46 mg/mL with 1.1 eq $TbCl_3$), 0.2 µL reservoir solution (0.96 M sodium citrate pH 7.0) and 0.1 µL of seed stock (1:10 dilution). First crystals were detected after 2 months of equilibration against 50 µL of reservoir solution at 20 °C. Crystals were cryoprotected by adding 1 µL of a 1:1 mixture of reservoir solution and 70 % (v/v) glycerol, vitrified, and stored in liquid nitrogen.

<u>Data collection and structure determination:</u> Diffraction images of MID1sc9_4xE were recorded using synchrotron radiation of $\lambda = 0.976$ Å at the P13 beamline from PETRAIII at DESY (Deutsches Elektronen-Synchrotron, Hamburg, Germany). The recorded reflections were processed using the XDS suite [4] (**Table S5**). Data reduction was performed with XSCALE.[4,5] The ACRIMBOLDO [6] algorithm was used for *ab initio*

phasing, and the model was built in COOT.[7] The model was then refined and completed by alternating restrained refinement with REFMAC5 [8] model building with COOT (v. 0.9).[7] Water molecules were placed with ARP/wARP 8.0,[9] and the structure was refined with anisotropic restraints to satisfactory R_{work} and R_{free} values. The geometry of the final structure was analyzed by the MOLPROBITY [10] online tool and deposited in the RCSB Protein Data Bank (PDB entry: 9S7R)

Table S5 | Crystallographic data collection and refinement statistics.

	MID1sc9_4xE
**Crystal parameters**	
Space group	$P1\ 2_1\ 1$
Cell constants	a= 29.1 Å
	b= 64.7 Å
	c= 46.7 Å
	β=107.9
**Data collection**	
Beam line	P13, PETRA III, DESY
Wavelength (Å)	0.976
Resolution range (Å)[b]	30.0-1.25 (1.35-1.25)
No. observations	153,189
No. unique reflections[c]	44,775
Completeness (%)[b]	98.4 (98.3)
R_{merge} (%)[b,d]	5.7 (69.7)
I/σ (I)[b]	11.4 (2.0)
**Refinement (REFMAC5)**	
Resolution range (Å)	27.61-1.25
No. refl. working set	42,536
No. refl. test set	2,239
No. non hydrogen	1,585
No. of ligand atoms	0
Solvent	119
R_{work}/R_{free} (%)[e]	14.9 / 17.4
r.m.s.d. bond (Å) / angle (°)[f]	0.006 / 1.3
Average B-factor (Å^2)	15.5
Ramachandran Plot (%)[g]	98.3 / 1.7/ 0
PDB accession code	9S7R

[a] Asymmetric unit
[b] The values in parentheses for resolution range, completeness, R_{merge} and I/σ (I) correspond to the highest resolution shell
[c] Data reduction was carried out with XDS and from a single crystal. Friedel pairs were treated as identical reflections
[d] $R_{merge}(I) = \Sigma_{hkl}\Sigma_j \mid I(hkl)_j - \langle I(hkl)\rangle \mid / \Sigma_{hkl} \Sigma_j I(hkl)_j$, where $I(hkl)_j$ is the jth measurement of the intensity of reflection hkl and <I(hkl)> is the average intensity
[e] $R = \Sigma_{hkl} \mid |F_{obs}| - |F_{calc}| \mid / \Sigma_{hkl} |Fobs|$, where R_{free} is calculated without a sigma cut off for a randomly chosen 5% of reflections, which were not used for structure refinement, and R_{work} is calculated for the remaining reflections
[f] Deviations from ideal bond lengths/angles
[g] Percentage of residues in favored region / allowed region / outlier region

Solid phase peptide synthesis

The peptides TrpZip2, Tz2H3 and LanPep1 were synthesized according to this procedure: A 10 mL fritted syringe was charged with 0.5 g chlorotrityl chloride resin (CTC; capacity: 1.01 mmol·g^{-1}) and swelled in 5 mL DCM for 20 min. 1.5 mol equivalents of the first Fmoc-protected amino acid and 3 equivalents of DIPEA were dissolved in 7 mL DCM. The solution was added to the drained resin and shaken for 1 h at room temperature. The resin was washed thoroughly with 2x 9 mL DCM and 2x 9 mL DMF. For N-terminal deprotection, the resin was treated twice with 6 mL of 20 % piperidine in DMF and 10 min incubation on a shaker. The resin was then washed with 3x 9 mL DMF. Subsequent amino acids were coupled with following sequence: 1) C-terminal activation: 2 – 4 equivalents of Fmoc-protected amino acid, 4 equivalents of DIPEA and 1.9 equivalents of HATU were dissolved in 7 mL DMF and incubated for 10 min at room temperature for activation, 2) Coupling: The resin was infused with the solution, incubated for 1 – 2 h at room temperature and washed with 2x 9 mL DCM and 2x 9 mL DMF, 3) N-terminal deprotection: 2x 6 mL of 20 % piperidine in DMF was added, incubated for 10 min with shaking and washed with 3x 9 mL DMF. A final wash with 3x 9 mL DMF and 3x 9 mL DCM was conducted. The peptide was cleaved from the resin by addition of 8 mL 20 % hexafluoroisopropanol in DCM and incubated for 20 min. This step was repeated once before washing with 2x 9 mL DCM. The eluted fractions were collected, and the solvent was evaporated *in vacuo*. For side chain deprotection, the peptide was resuspended in 20 mL solution of 90 % TFA, 5 % H_2O and 5 % DCM and stirred for 3-4 h. When the reaction was complete, the TFA was removed *in vacuo* yielding a brownish oil.

LanPep2 was synthesized with small deviation to the standard protocol: A 10 mL fritted syringe was charged with 0.25 g CTC resin (capacity: 1.2 mmol·g^{-1}) and swelled in 5 mL DCM for 20 min. 1.5 mol equivalents of Fmoc-Glu(OtBu) and 3 equivalents of DIPEA were dissolved in 3 mL DCM. The solution was added to the drained resin and incubated for 1 h at room temperature, then washed with 3x 9 mL DCM and 3x 9 mL DMF. The charged resin was transferred to a 250-mL fritted SPPS synthesis column for subsequent amino acids coupling. All steps were conducted under a constant stream of N_2: 1) N-terminal deprotection: 20 mL 5% (w/v) piperazine in DMF was added and heated on an oil bath at 70°C for 1 min. This step was repeated once with 5 min heating, followed by washing with DMF (4x 9 mL, 30 s residence time). 1) C-terminal

activation: A solution of 1.5 – 3 equivalents Fmoc-protected amino acid, 2 equivalents HCTU and 6 equivalents DIPEA in 3 mL DMF was prepared and incubated for at least 10 min at room temperature. 2) Coupling: Resin was infused with the activated amino acid solution, and the coupling was proceeded at 70°C for 7 min, followed by a wash with DMF (2x 10 mL, 1 min residence time). 4) Acetylation: Resin was infused with 10 mL solution of 10 equivalents of Ac_2O/DIPEA in DMF and incubated for 15 min, followed by resin wash with DMF (2x 10 mL, 1 min residence time). 80 – 90 mg of the charged resin was transferred to a 10-mL fritted syringe, and the peptide was cleaved by infusing with 2.5 mL cleavage solution (90% *v/v* TFA, 5% *v/v* dimethyl sulfide, 2.5% *v/v* H_2O, 2.5% *v/v* Triisopropylsilane) and incubating at room temperature for 4 h while shaking. The solution was drained into 45 mL of cold diethyl ether (-70°C) and the resin was washed with additional 0.5 mL TFA. The organic phase was centrifuged (11.000xg, 30 min, 4°C). The pellet was washed with an additional 20 mL cold diethyl ether and dried under vacuum. A crude solid of 142 – 189 mg was obtained.

For preparative reverse-phase HPLC purification, the crude peptides were dissolved in 10% ACN/0.5% TFA and either passed through a syringe filter (CA, 0.2 µm) or centrifuged for 5-10 min. All peptides were purified on a Waters Alliance 2695 quaternary gradient module equipped with a X-Bridge™ BEH C18 OBD Prep Column (30 x 150 mm, 5 µm), a Waters 2998 PDA detector and a Waters Fraction Collector III. Solvent A and B consisted of H_2O and ACN, with 0.1% TFA. Method: Equilibration with solvent A, sample injection (10 mL), 21 min gradient 5-20% solvent B, 5 min wash with solvent A; Flow rate: 50 $mL \cdot min^{-1}$. 10 mL fractions were collected at the gradient according to an absorbance threshold of 10% at 280 nm. Fractions containing the peptide were combined and lyophilized.

Prediction of the Ln^{3+} binding site in MID1sc9_4xE

To predict the location of the binding site for Ln^{3+} ions, the *BioMetAll* tool was utilized on the crystal structure of MID1sc9_4xE (PDB entry: 9S7R, Chain A) with default settings and consideration of three or four coordinating residues, respectively. Furthermore, AlphaFold3 was used with default settings for structure prediction with bound Tb^{3+}.

2) Supplementary Figures

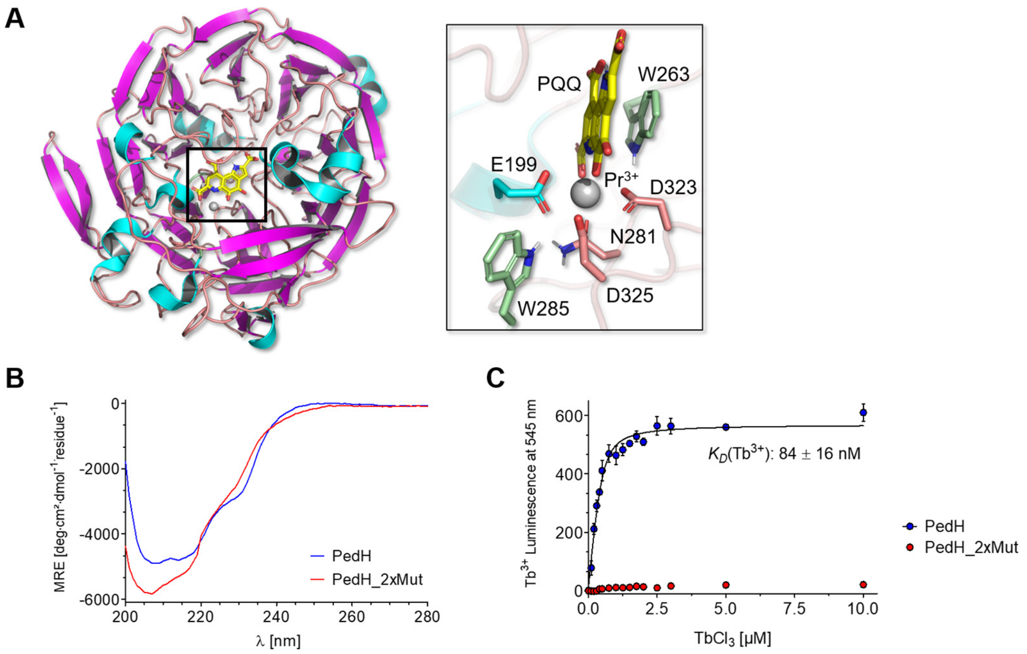

Figure S1 | The lanthanide-dependent alcohol dehydrogenase PedH. (A) Cartoon representation of the crystal structure (PDB entry: 6czv). The inset shows PQQ cofactor (yellow sticks), Pr^{3+} (grey sphere), coordinating residues E199 (cyan sticks), D323, D325 and N281 (salmon sticks) and the Trp residues W285 and W263 (green sticks). (B) CD spectra of PedH-His$_6$ and PedH_2xMut-His$_6$. (C) Tb^{3+} binding curve of PedH and PedH_2xMut (D323N/D325N), where 0.5 µM protein was titrated with 0 – 10 µM $TbCl_3$. The solid line represents a quadratic fit with the respective K_D = 84 nM.

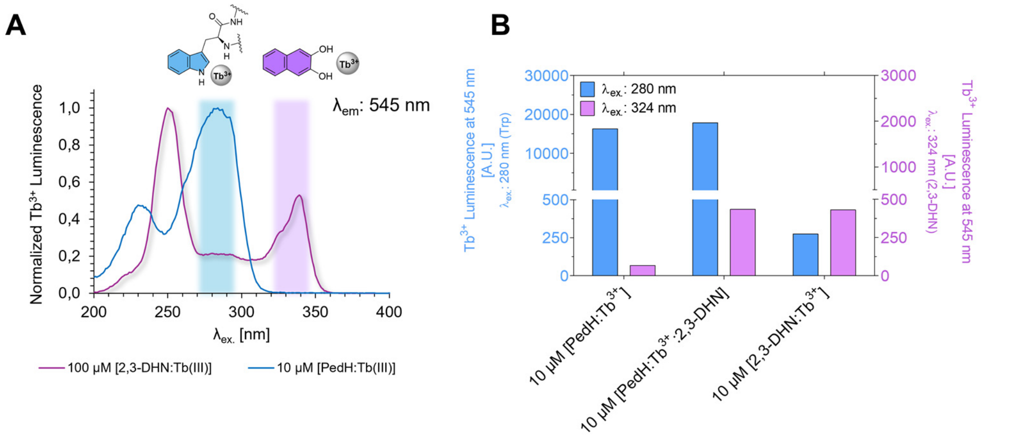

Figure S2 | Spectral characteristics of terbium luminescence signals sensitized by Trp and 2,3-DHN. (A) Excitation scans of PedH (blue, 10 µM) and 2,3-DHN (purple, 100 µM) in the presence of equimolar $TbCl_3$. The spectra were recorded at a fixed emission wavelength of λ = 545 nm in 25 mM HEPES, 100 mM NaCl, pH 7.0. The shaded areas indicate most suitable excitation wavelengths for Trp and 2,3-DHN sensitization, respectively. (B) Sensitized Tb^{3+} luminescence of equimolar mixtures of [PedH:Tb^{3+}], [2,3-DHN:Tb^{3+}] and [PedH:2,3-DHN:Tb^{3+}] (10 µM) at the specified excitation wavelengths.

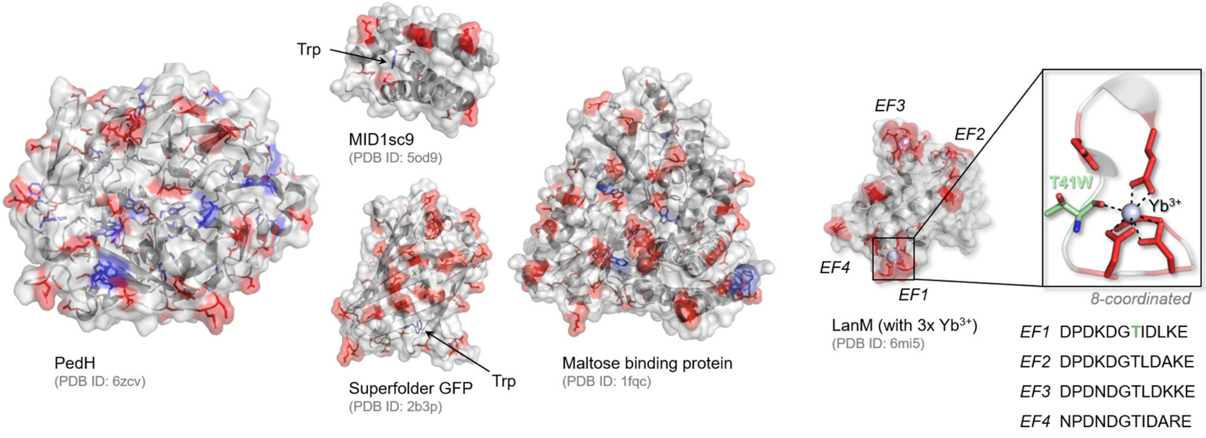

Figure S3 | Surface representation of control proteins selected for assay validation. Asp/Glu residues are shown as red sticks and Trp residues as blue sticks. Single intrinsic Trp residues of *sf*GFP and MID1sc9 are indicated. The EF hand motifs of LanM are mapped on the structure and the respective sequences are listed. The inset shows the EF1 loop with bound Yb^{3+} ion and coordinating residues (colored sticks). The T41W mutation was introduced to generate a Trp-sensitized terbium luminescence readout.

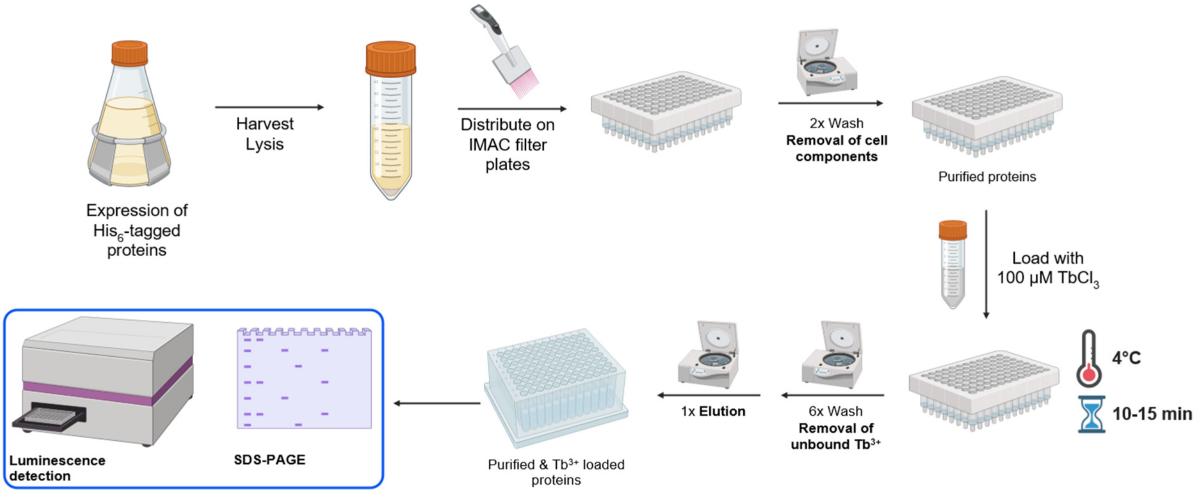

Figure S4 | Workflow for on-resin Tb^{3+} loading with uniform cell lysates for assay validation. The figure was created with BioRender (https://BioRender.com/g76mqpl).

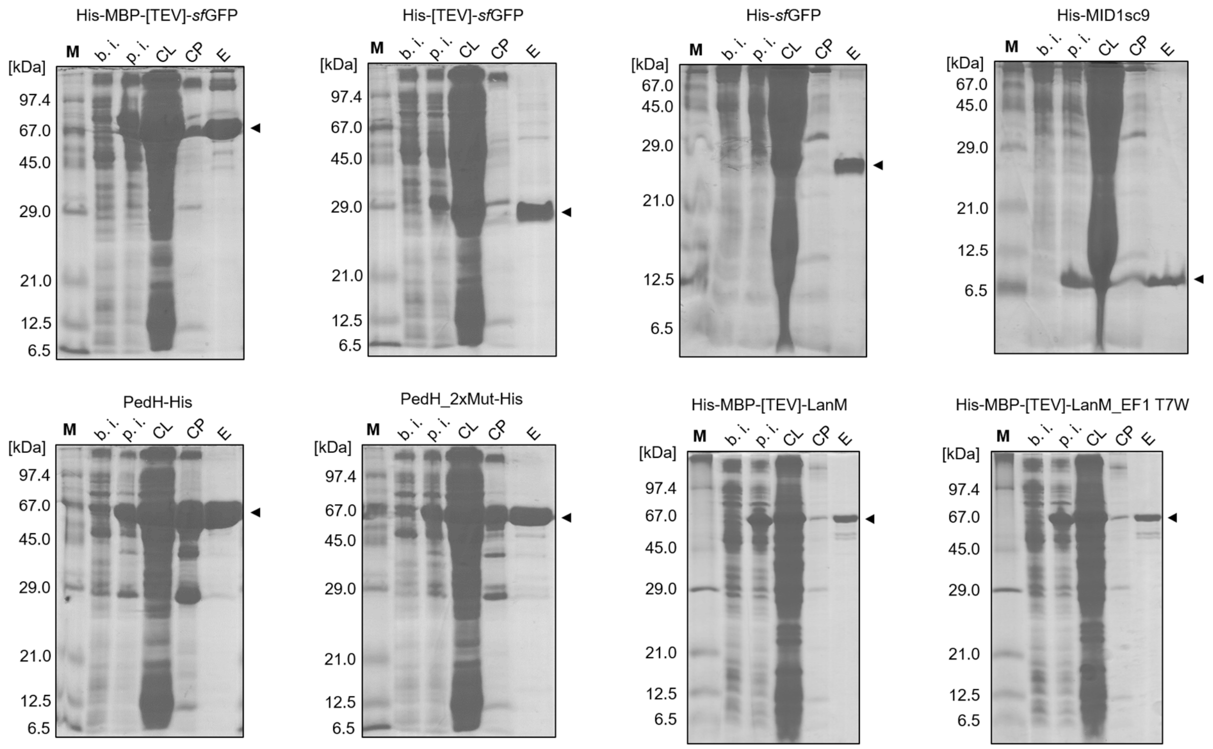

Figure S5 | Plate purification of control proteins. Indicated proteins were produced in shake flasks as described above. Samples of whole cells before induction (b. i.) and post induction (p. i.), cell lysate (CL) cell pellet (CP) and elution fraction (E) of Tb^{3+} loaded proteins were analyzed on denaturing polyacrylamide gel electrophoresis. Black triangles indicate the expected protein band.

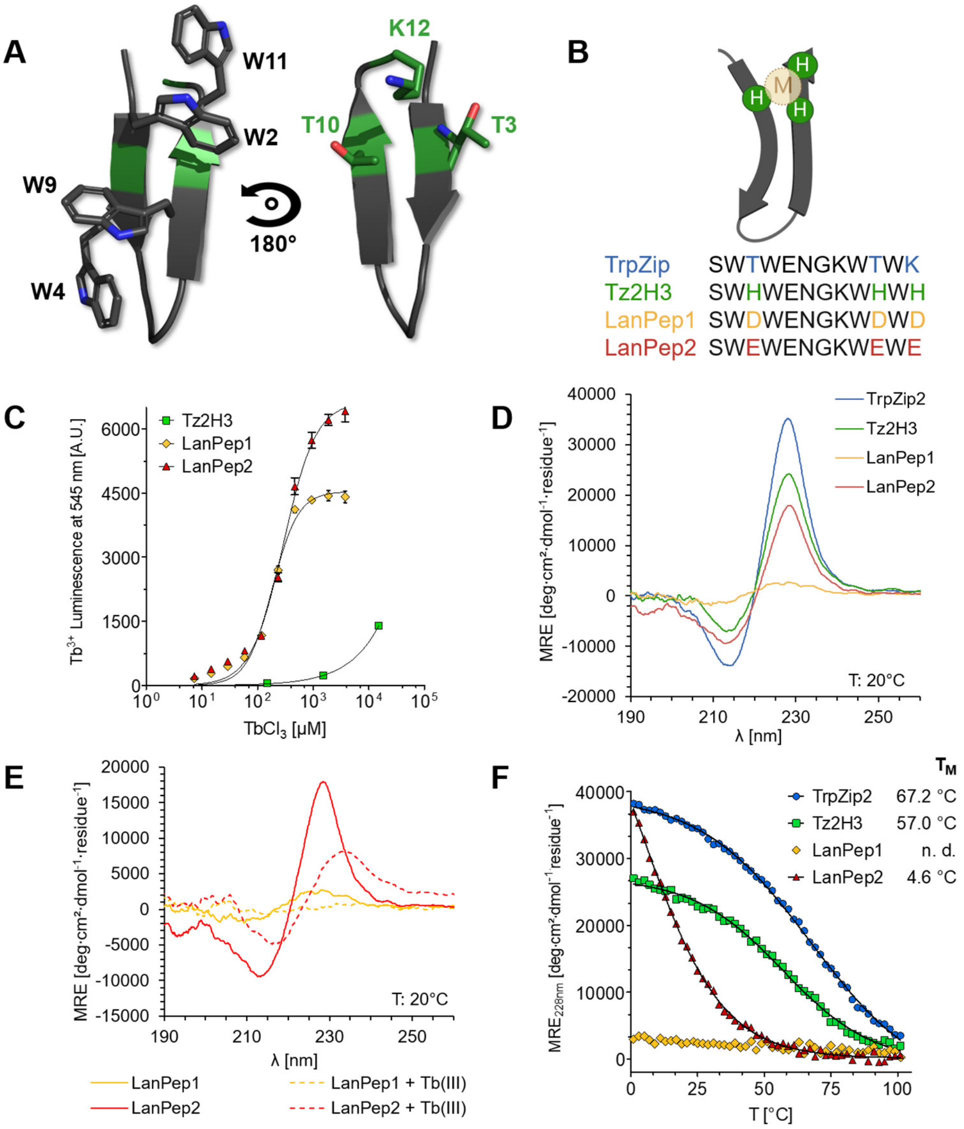

Figure S6 | Characterization of synthetic Trp zipper peptides. (A) NMR structure of the original TrpZip peptide (PDB ID: 1LE1) with Trp residues shown as black sticks. The metal-coordinating residues were later installed on the opposite face of the strands (residues are highlighted as green sticks). (B) Schematic representation of a Trp zipper with marked positions of the metal-coordinating residues. Sequences of the tested peptides are listed below. Parts of this figure were generated with BioRender: https://BioRender.com/npve054. (C) Tb^{3+} binding titrations using Trp-sensitized luminescence as the readout. 15 µM peptide was prepared in 25 mM HEPES, 100 mM NaCl, pH 7.0. The K_D values were determined using a quadratic equation fit, yielding a K_D of 184 ± 9 µM for LanPep1 and 292 ± 17 µM for LanPep2. For the control peptide Tz2H3, no K_D value could be determined within the titration range. (D) CD spectra of 50 µM peptide in 10 mM HEPES, 100 mM NaCl, pH 7.0 at 20°C. (E) CD spectra of 50 µM LanPep1 and LanPep2 in the absence and presence of 5 mM $TbCl_3$ at 20°C. The reduced spectral intensities observed for both peptides in the presence of $TbCl_3$ result from partial precipitation. (F) CD melting curves of respective peptide. The melting temperature T_M was determined using a sigmoidal Boltzman fit if applicable. n. d. – not determined

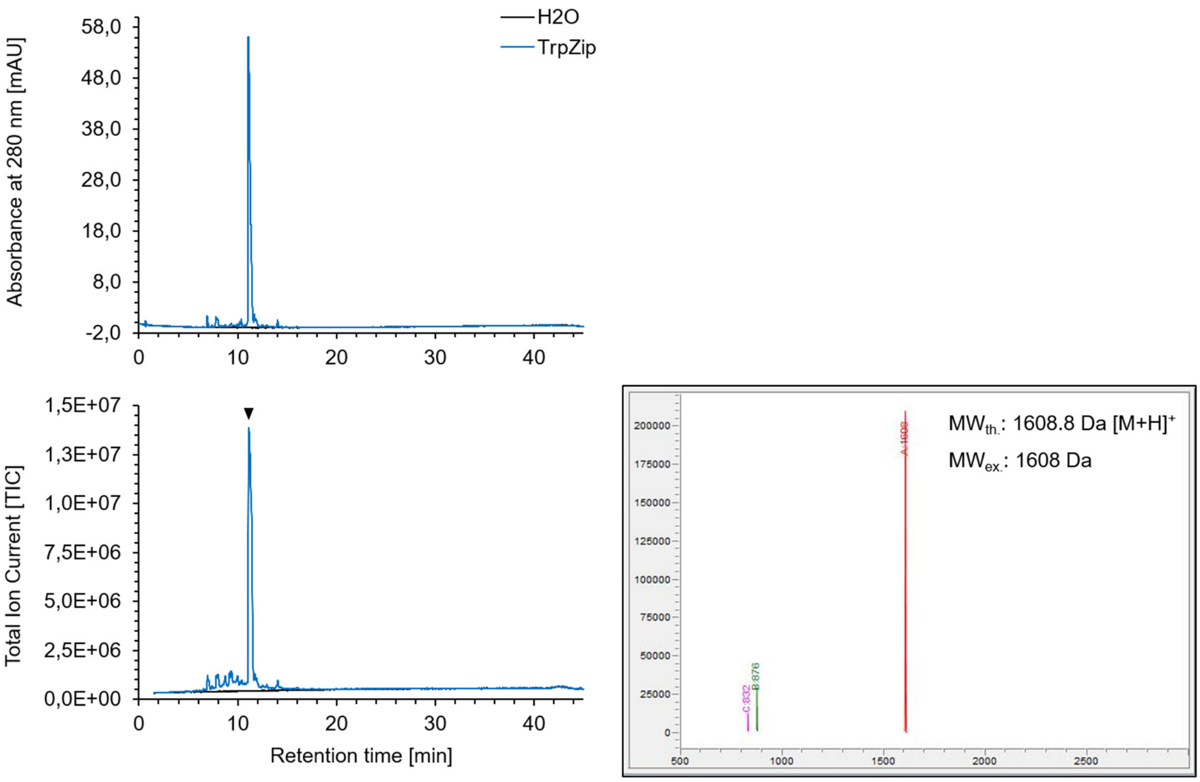

Figure S7 | TrpZip2 peptide. LC-MS analysis of 100 µM TrpZip. 2 µL were injected and analyzed on a Poroshell 300SB column. Shown traces are UV/Vis absorbance at 280 nm (upper) and total ion current in positive ionization mode (lower). The deconvoluted mass spectrum (right) was generated from the indicated peak. $MW_{theoretical}$: 1608.8 Da; $MW_{experimental}$: 1608 Da.

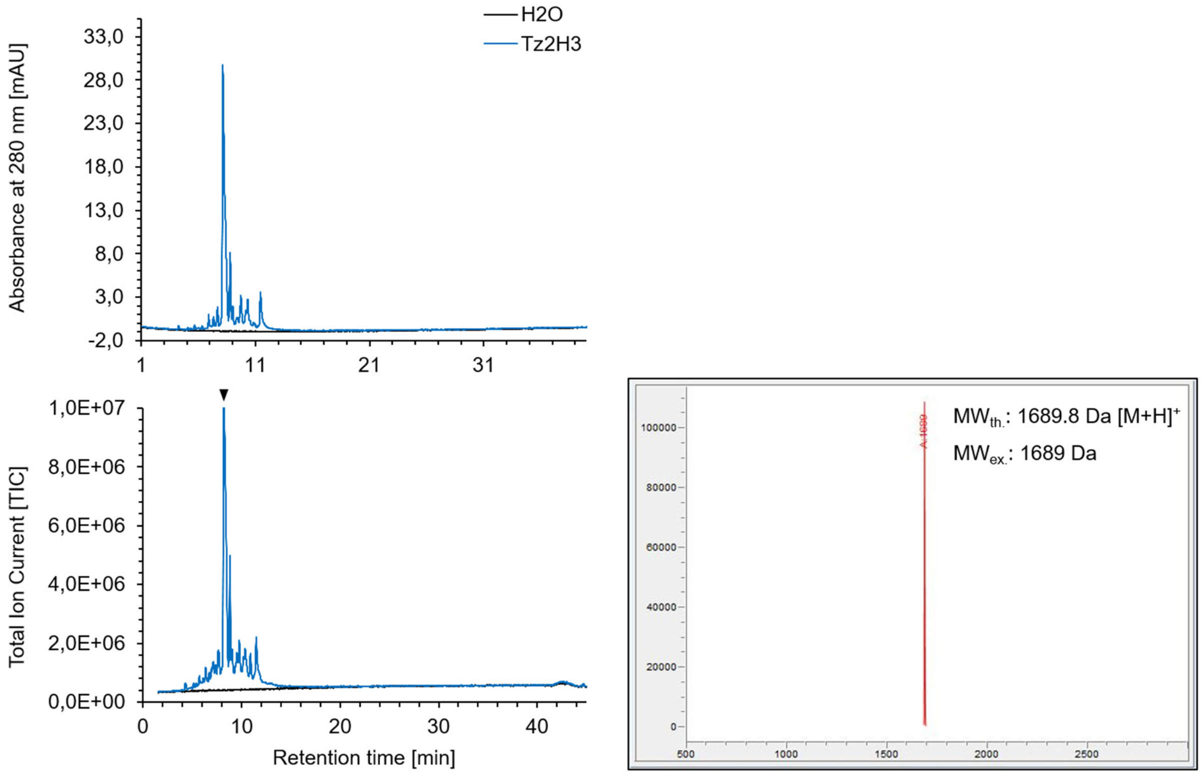

Figure S8 | Tz2H3 peptide. LC-MS analysis of 100 µM Tz2H3. 2 µL were injected and analyzed on a Poroshell 300SB column. Shown traces are UV/Vis absorbance at 280 nm (upper) and total ion current in positive ionization mode (lower). The deconvoluted mass spectrum (right) was generated from the indicated peak. $MW_{theoretical}$: 1689.8 Da; $MW_{experimental}$: 1689 Da.

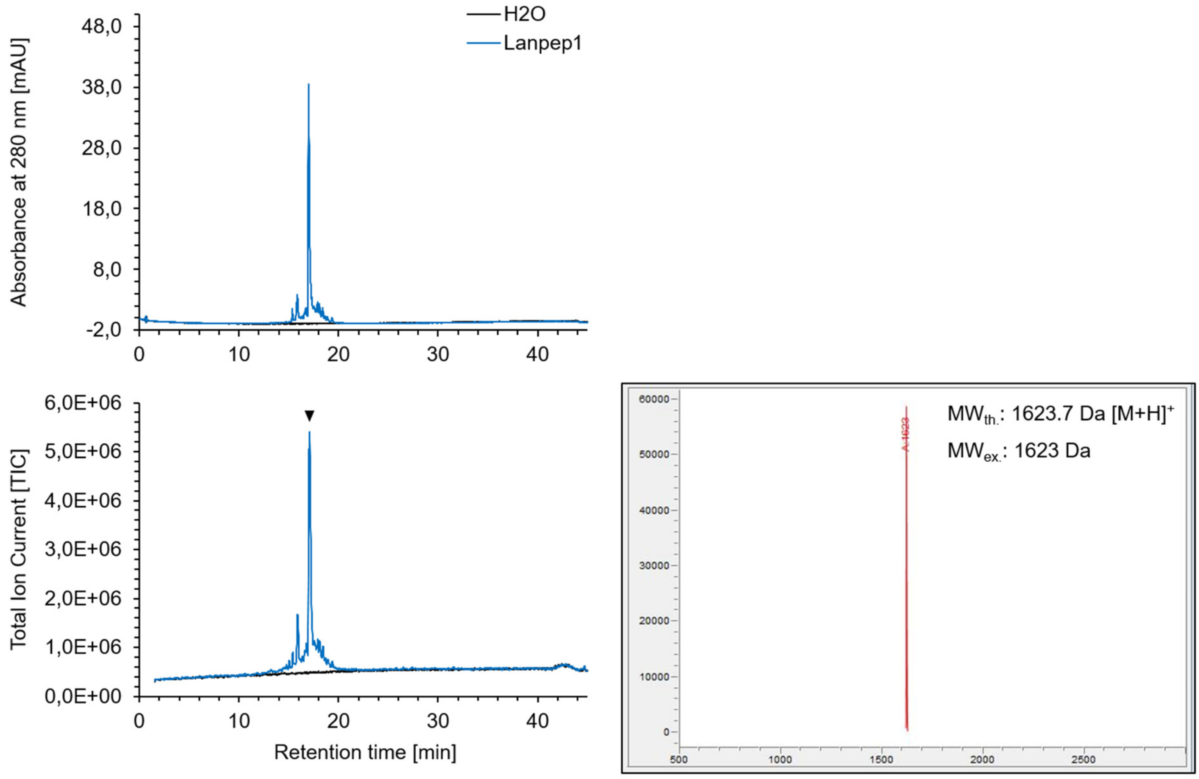

Figure S9 | LanPep1 peptide. LC-MS analysis of 100 µM LanPep1. 2 µL were injected and analyzed on a Poroshell 300SB column. Shown traces are UV/Vis absorbance at 280 nm (upper) and total ion current in positive ionization mode (lower). The deconvoluted mass spectrum (right) was generated from the indicated peak. $MW_{theoretical}$: 1623.7 Da; $MW_{experimental}$: 1623 Da.

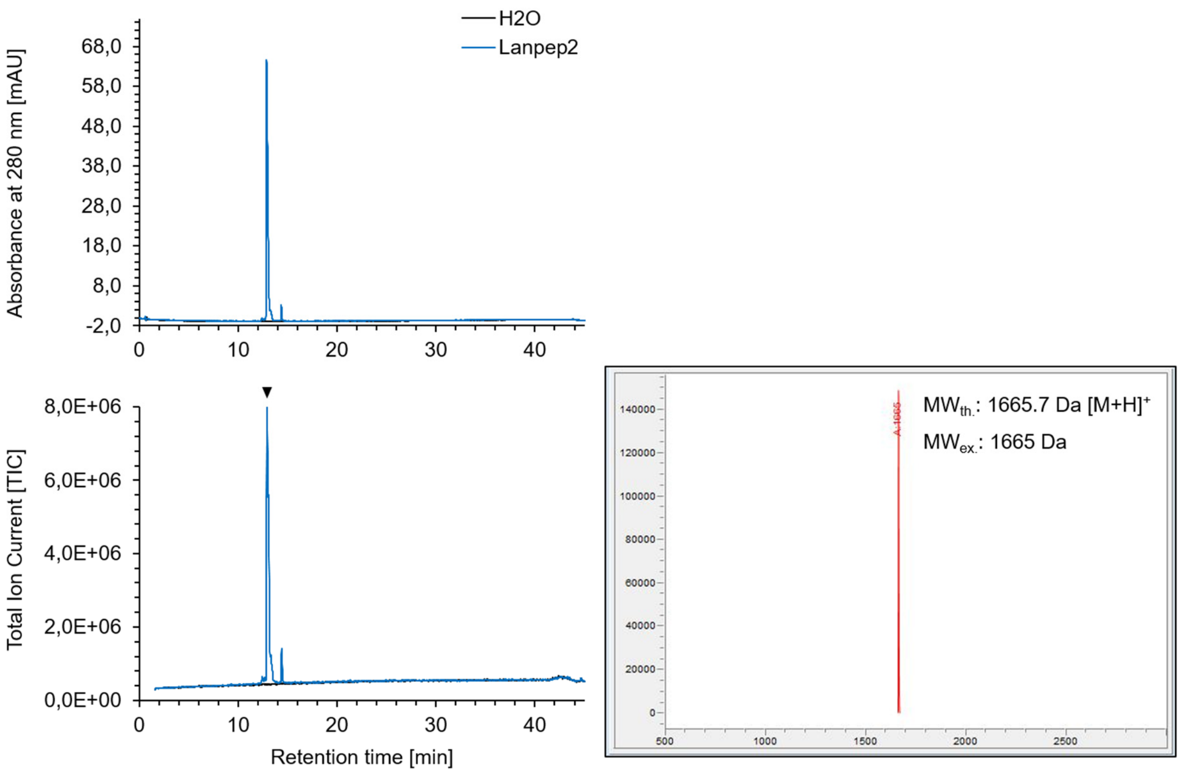

Figure S10 | LanPep2 peptide. LC-MS analysis of 100 µM LanPep2. 2 µL were injected and analyzed on a Poroshell 300SB column. Shown traces are UV/Vis absorbance at 280 nm (upper) and total ion current in positive ionization mode (lower). The deconvoluted mass spectrum (right) was generated from the indicated peak. $MW_{theoretical}$: 1665.7 Da; $MW_{experimental}$: 1665 Da.

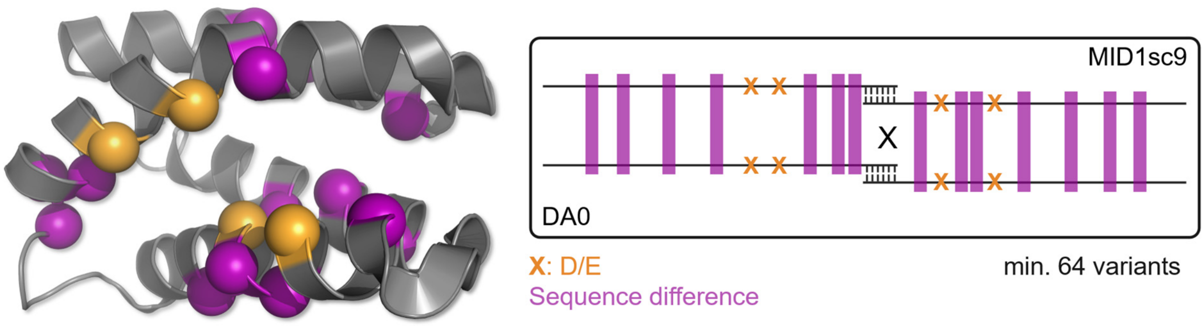

Figure S11 | Library design based on the MID1sc scaffold. The metal-coordinating positions 35, 39, 61, 65 are shown in orange. The positions in which the two template sequences differ are shown in pink. The genes were split to create the oligo pools and recombined via overlap-extension PCR (as described above).

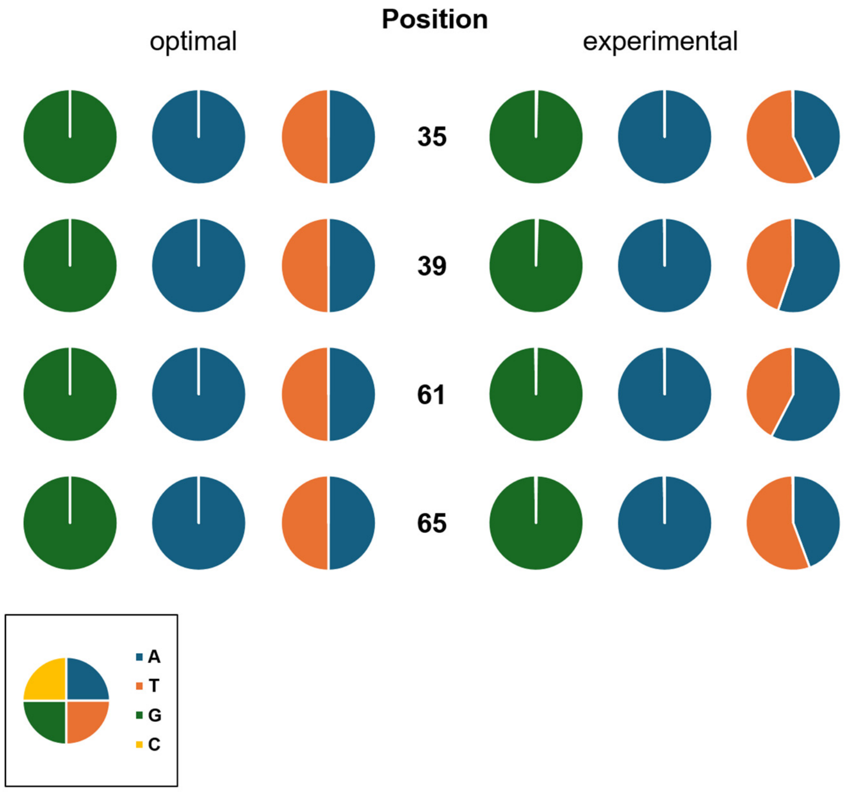

Figure S12 | Nucleotide distribution at the metal-coordinating position 35, 39, 61, 65 of the MID1 scaffold. The triplets GAT and GAA code for Asp and Glu, respectively.

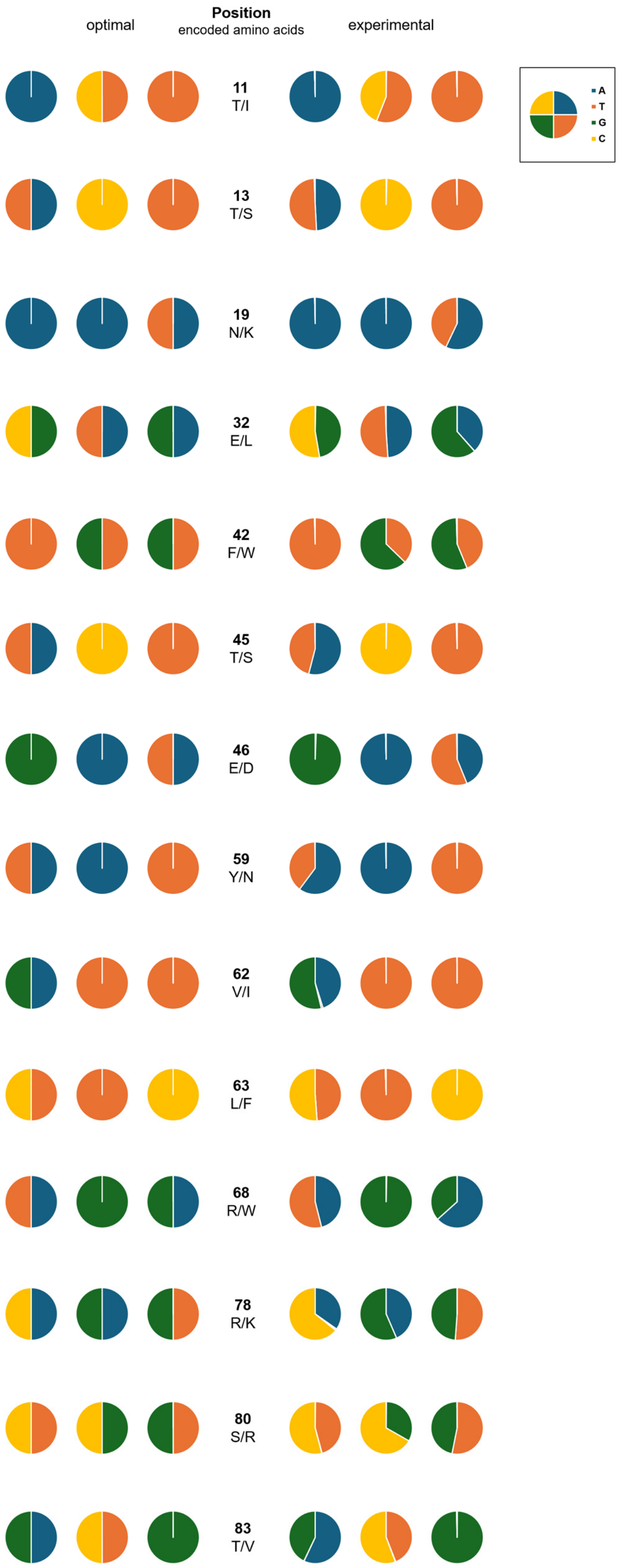

Figure S13 | Nucleotide distribution for the positions that differ between the two template sequences. Due to mutational shuffling in these positions, the actual library size likely increased significantly beyond the theoretical minimum of 64 variants, if no shuffling between the oligo pool sequences had occurred.

A

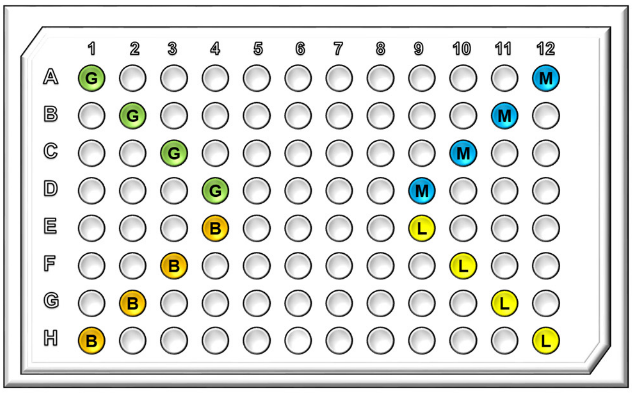

B

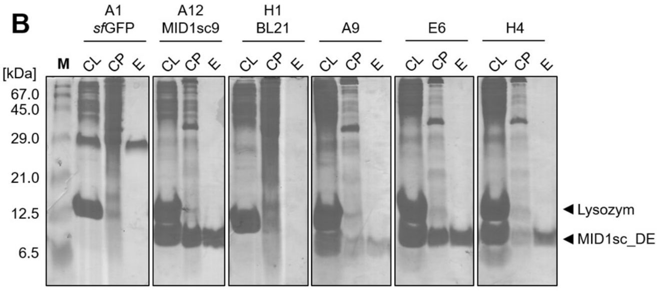

C

	1	2	3	4	5	6	7	8	9	10	11	12
A	83,38	234,3	5779	72,17	1055	111,4	67,4	168	12160	7110	59,67	53,82
B	10290	68,74	983	10970	267,7	821,9	236,4	5052	7892	1123	43,51	8669
C	159,7	1709	55,66	268,4	103,4	100,2	144,9	270,4	16180	44,73	25500	80,51
D	11890	927	29870	54,23	5149	128,9	228,9	153,4	42,17	1038	14660	140,8
E	62,74	17890	15150	52,95	65,86	23660	82,25	1297	31,56	474,5	615,5	947,9
F	768,4	5103	53,34	107,3	6053	2360	503,4	439,6	4802	40,49	1400	3119
G	18530	55,39	226,1	5925	103,7	195,2	288,8	6265	752	89,08	43,95	2904
H	56,76	66,02	63,14	15220	184,9	822,4	207	4301	2303	171,1	921,2	40,41

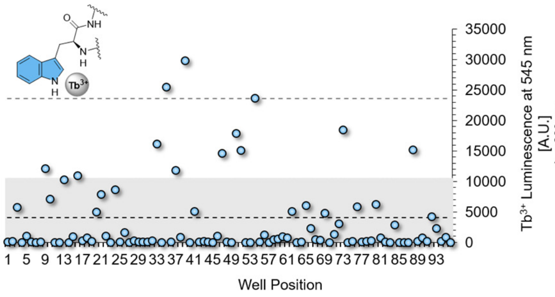

	1	2	3	4	5	6	7	8	9	10	11	12
A	70,94	88,49	413	65,32	103,3	77,97	64,69	71,66	427,2	131,2	59,29	75,41
B	176,3	68,71	104,6	162,9	60,26	104,9	57,7	129	161,8	95,3	53,63	279,5
C	85,77	155,1	51,89	66,94	50,47	63,99	60,4	88,13	280,6	50,78	306,1	64,23
D	412,9	113,6	293,9	54,59	315,2	62,55	84,92	56,04	42,71	87,56	147,5	65,47
E	68,94	377,8	139,2	58,55	59,57	615,1	60,33	91,45	36,54	87,77	80,7	76,98
F	101,1	306,7	55,25	69,7	362,6	123,4	73,19	93,47	305,6	49,94	299,7	102,9
G	517	56,56	75,27	343,1	52,92	62,94	59,47	429,7	82,58	69,7	47,79	130,9
H	65,78	74,12	61,72	145	88,42	124,2	66,31	146,7	89,8	76,31	229,3	46,13

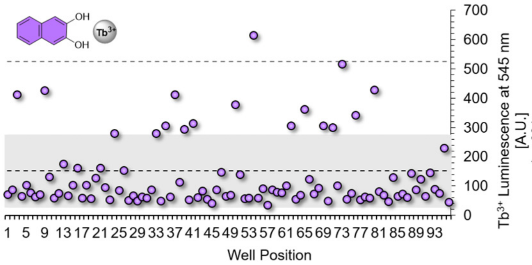

	1	2	3	4	5	6	7	8	9	10	11	12
A	5915	20733	2386727	4714	108982	8686	4360	12039	5194752	932832	3538	4059
B	1814127	4723	102822	1787013	16132	86217	13640	651708	1276926	107022	2333	2422986
C	13697	265066	2888	17967	5219	6412	8752	23830	4540108	2271	7805550	5171
D	4909381	105307	8778793	2960	1622965	8063	19438	8597	1801	90887	2162350	9218
E	4325	6758842	2108880	3100	3923	14553266	4962	118611	1153	41647	49671	72969
F	77685	1565090	2947	7479	2194818	291224	36844	41089	1467491	2022	419580	320945
G	9580010	3133	17019	2032868	5488	12286	17175	2692071	62100	6209	2100	380134
H	3734	4893	3897	2206900	16349	102142	13726	630957	206809	13057	211231	1864

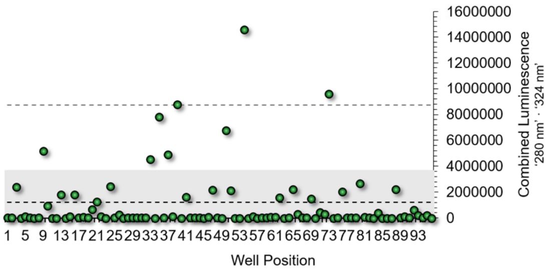

Figure S14 | Exemplary plate screening results for the MID1sc_DE library. (A) Plate layout of a typical screening plate. Control wells of *E. coli* BL21(DE3) GOLD expressing His$_6$-sfGFP (G) or His$_6$-MID1sc9 (M) as well as uninduced cells (B) and sterile controls with medium only (L) were included. (B) SDS-PAGE of cell-free extract (CL), cell pellet (CP), and elution fraction (E) samples taken from indicated wells. Lysozyme (14.0 kDa) and MID1sc_DE (~12 kDa) are indicated with black triangles. (C) Sensitized Tb^{3+} luminescence from Trp (top, internal antenna, excited at 280 nm), 2,3-DHN (middle, external antenna, excited at 324 nm) and combined values (bottom). The values were color coded from red (value = 0) to green (highest value) and are displayed in the adjacent plots. The black dashed line represents the overall average of the detected luminescence values (excluding the control wells). The grey area and the grey dashed line represent the 1x standard deviation (SD) and 3x SD, respectively, with latter one used as a threshold for hit identification. Potential hits from the individual channels were highlighted in pale blue and subjected to a second screening round. Additional wells close to the threshold (marked with bold lines) were also included for re-screening.

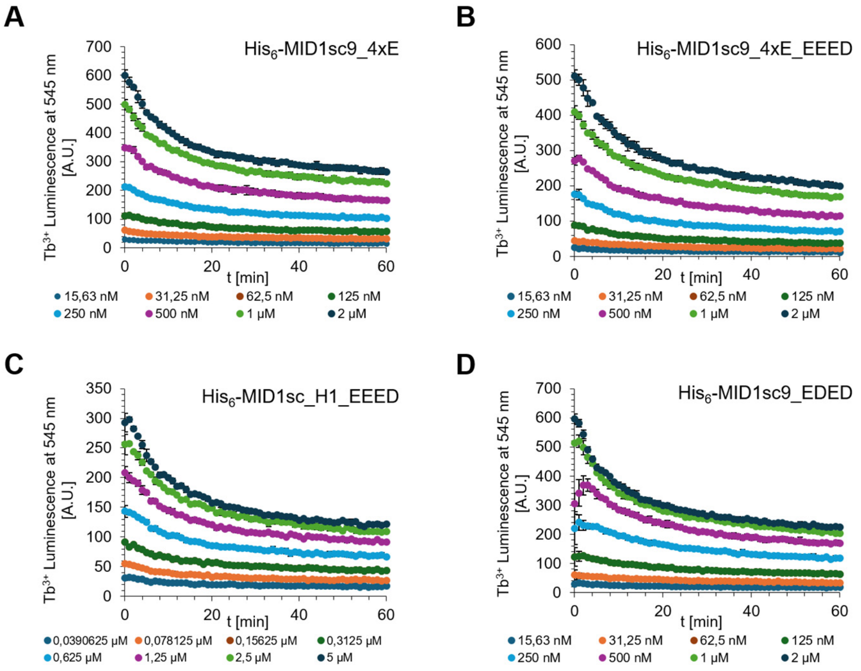

Figure S15 | Decline of the Tb^{3+} luminescence signal over time upon addition of $TbCl_3$ to the protein. (A) MID1sc9_4xE (B) MID1sc9_EEED (C) MID1sc9_H1_EEED (D) MID1sc9_EDED. Trp-sensitized Tb^{3+} luminescence was monitored at 545 nm for 1 h at 1 min intervals. The values shown are averages from technical triplicates and standard deviation represent error bars.

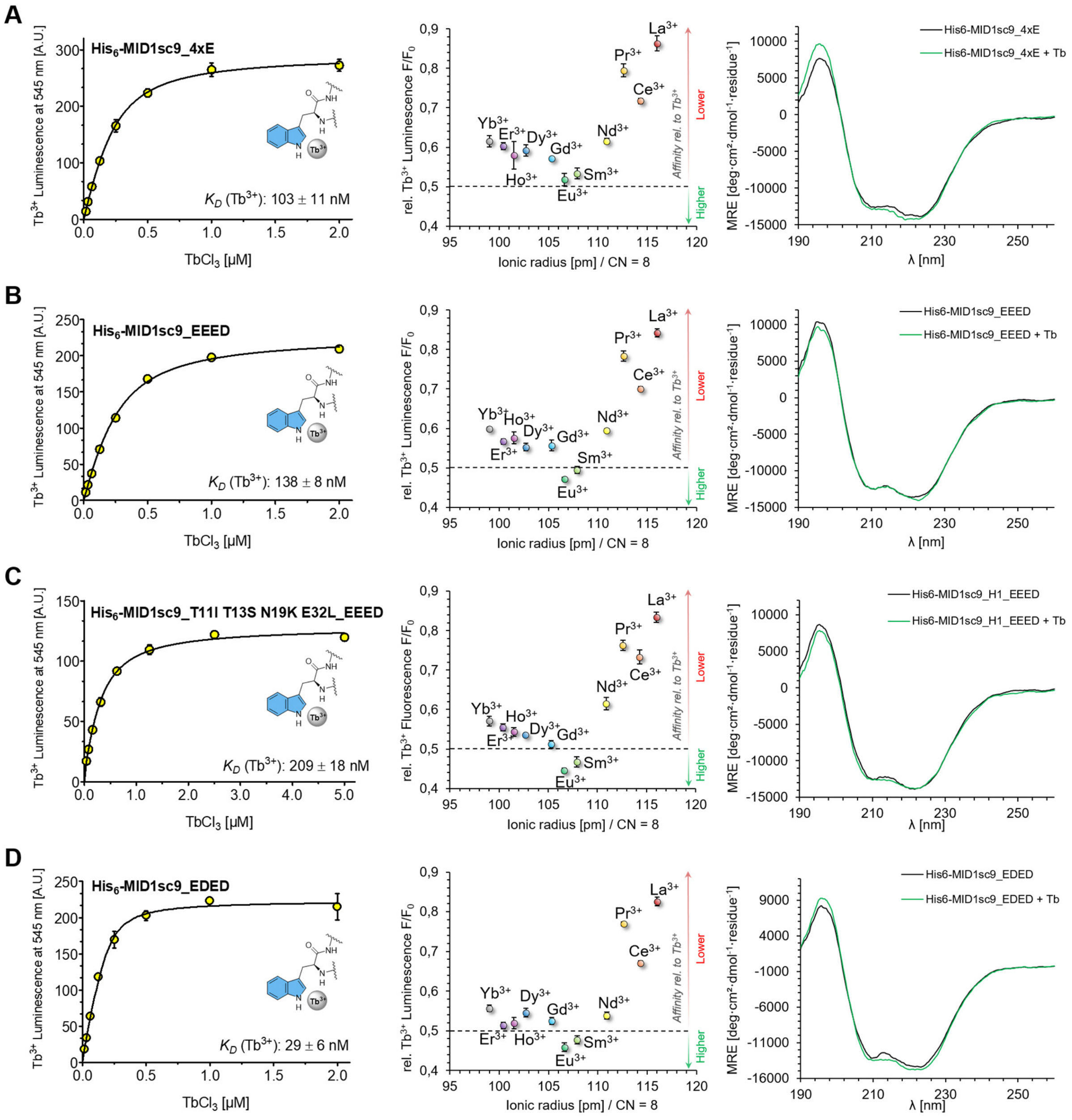

Figure S16 | Characterization of Tb^{3+} binding scaffolds identified in the screening assay. (A) MID1sc9_4xE, (B) MID1sc9_EEED, (C) MID1sc9_H1_EEED and (D) MID1sc9_EDED were studied by Tb^{3+} luminescence titration (left), Tb^{3+} displacement with other lanthanide ions (middle), and CD spectroscopy in the presence and absence of $TbCl_3$ (right). Tb^{3+} titration and displacement measurements were performed in technical triplicates and reported as average and standard deviation, represented by error bars.

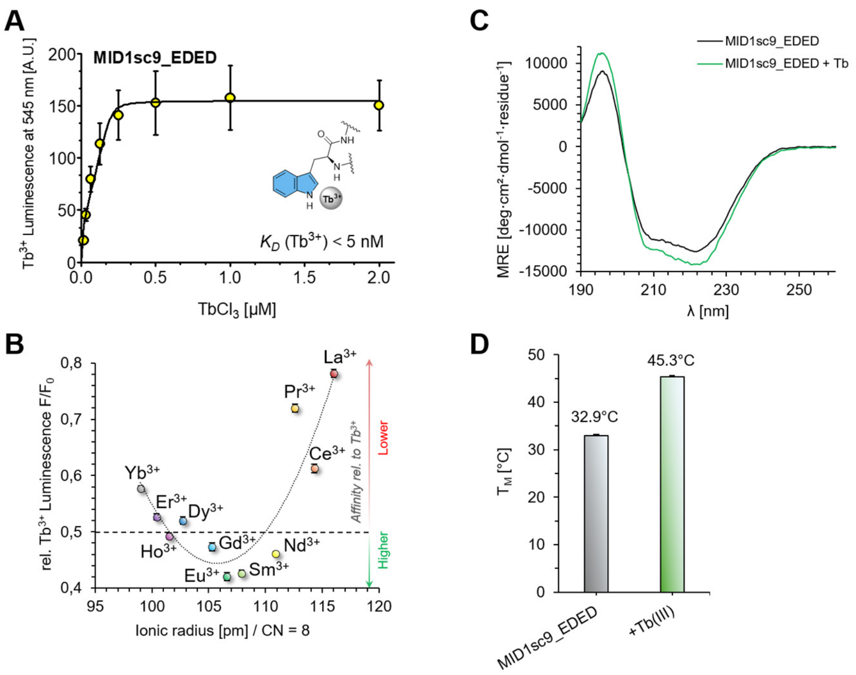

Figure S17 | Characterization of tagless MID1sc9_EDED. (A) The Tb^{3+} affinity was determined by Trp-sensitized luminescence. 200 nM protein was titrated with 15.6 nM to 2.0 µM $TbCl_3$. However, the low apparent K_D of MID1sc9_EDED could not be adequately fitted using a quadratic equation. Thus, the affinity was approximated to be lower than 5 nM. (B) Ln^{3+} selectivity was assessed by Tb^{3+} displacement. A 1:1 mix of 1 µM MID1sc9_4xE and $TbCl_3$ was mixed with equimolar $LnCl_3$ and incubated for 1 h at 25°C. The selectivity is expressed by the luminescence decrease, referenced to the signal of MID1sc9_4xE:Tb^{3+}, and plotted against the ionic radius of an octa-coordinated Ln^{3+} ion. The dashed line at 0.5 indicates that the Ln^{3+} affinity is equal to that of Tb^{3+}. The data are reported as average from technical triplicates with standard deviation as error bars. The dotted curve highlights the trend and does not represent an actual fit. (C) CD spectra of 10 µM MID1sc9_EDED in the presence and absence equimolar $TbCl_3$ in 20 mM NH_4HCO_3, pH 8.0 and at 20°C. (D) Thermal denaturation analyses of metal-free and $TbCl_3$ treated MID1sc9_EDED were performed to determine the melting temperatures T_M. The shown data are averages from technical duplicates with standard deviation as error bars.

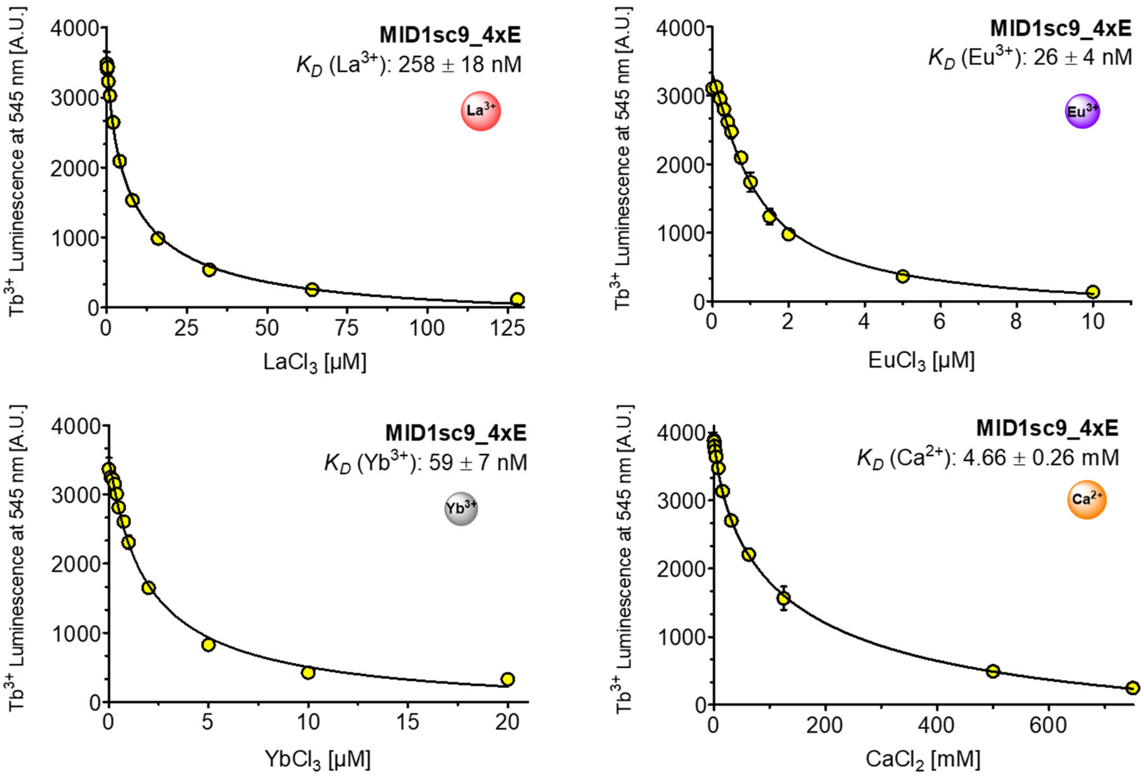

Figure S18 | Ln^{3+} and Ca^{2+} affinity of MID1sc9_4xE determined by Tb^{3+} displacement titrations. A pre-incubated solution of 1 µM MID1sc9_4xE and 1 µM $TbCl_3$ was titrated against 0-125 µM $LaCl_3$, 0-10 µM $EuCl_3$, 0-20 µM $YbCl_3$, and 0-750 mM $CaCl_2$, respectively, and the Trp-sensitized Tb^{3+} luminescence was measured. The affinities were determined using a cubic equation fit (with reference K_D = 32 nM for Tb^{3+}). The data are reported as averages from technical triplicates with standard deviation as error bars.

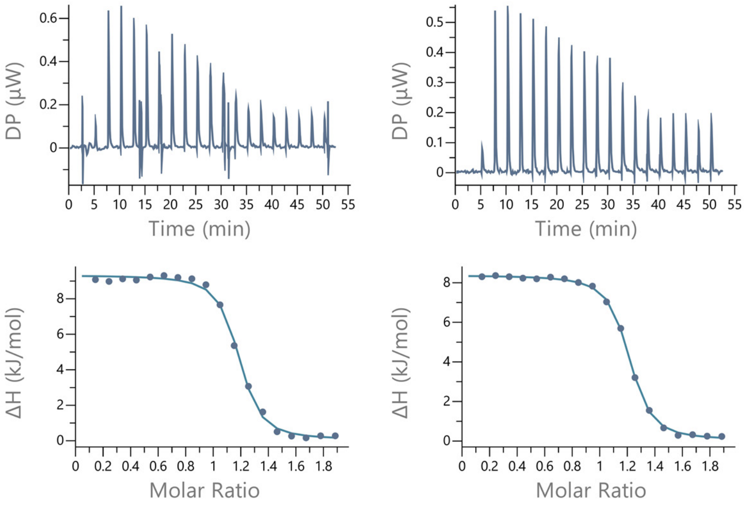

Tb^{3+} MID1sc9_4xE	1st Replicate	2nd Replicate	Average
N **(sites)**	1.13 ± 0.007	1.16 ± 0.003	**1.15**
K_D **(Tb^{3+}) [nM]**	133 ± 21.8	132 ± 8.4	**133**
ΔH **(kJ·mol^{-1})**	9.27 ± 0.16	8.33 ± 0.06	**8.8**
ΔG **(kJ·mol^{-1})**	-39.3	-39.3	**-39.3**
-TΔS **(kJ·mol^{-1})**	-48.6	-47.6	**-48.1**

Figure S19 | ITC measurement of Tb^{3+} binding to MID1sc9_4xE. 25 µM MID1sc9_4xE was titrated with freshly prepared 0.5 mM $TbCl_3$ in identical buffer (25 mM HEPES, 100 mM NaCl, pH 7.0), with injection volumes of 1x 0.4 µL and 18x 1 µL. Baseline and off-set of the thermograms were subtracted and differential enthalpy values were fitted. Results are derived from two independent measurements. The determined stoichiometry N, affinity K_D, and thermodynamic values ΔH, ΔG, and -TΔS are shown in the table. The average was calculated from both replicates.

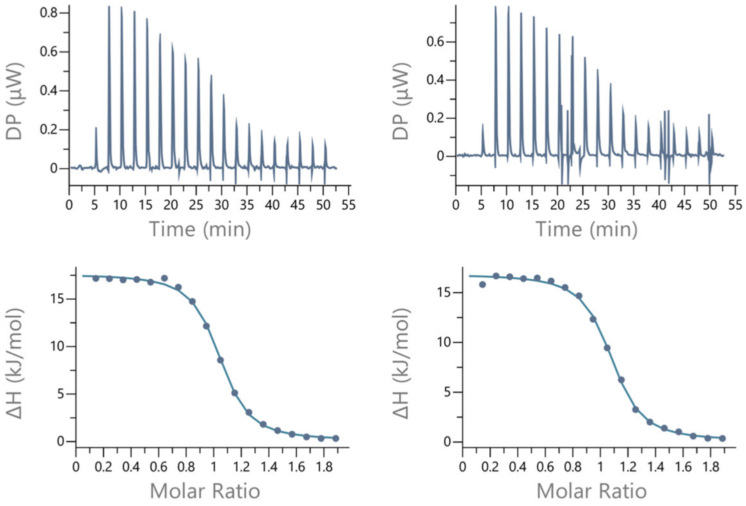

La^{3+} MID1sc9_4xE	1st Replicate	2nd Replicate	Average
N **(sites)**	1.00 ± 0.005	1.03 ± 0.007	**1.02**
K_D **(La^{3+}) [nM]**	288 ± 27.0	297 ± 34.1	**293**
ΔH **(kJ·mol^{-1})**	17.5 ± 0.21	16.8 ± 0.26	**17.2**
ΔG **(kJ·mol^{-1})**	-37.4	-37.3	**-37.4**
-TΔS **(kJ·mol^{-1})**	-54.9	-54.1	**-54.5**

Figure S20 | ITC measurement of La^{3+} binding to MID1sc9_4xE. 25 µM MID1sc9_4xE was titrated with freshly prepared 0.5 mM $LaCl_3$ in identical buffer (25 mM HEPES, 100 mM NaCl, pH 7.0), with injection volumes of 1x 0.4 µL and 18x 1 µL. Baseline and off-set of the thermograms were subtracted and differential enthalpy values were fitted. Results are derived from two independent measurements. The determined stoichiometry N, affinity K_D, and thermodynamic values ΔH, ΔG, and -TΔS are shown in the table. The average was calculated from both replicates.

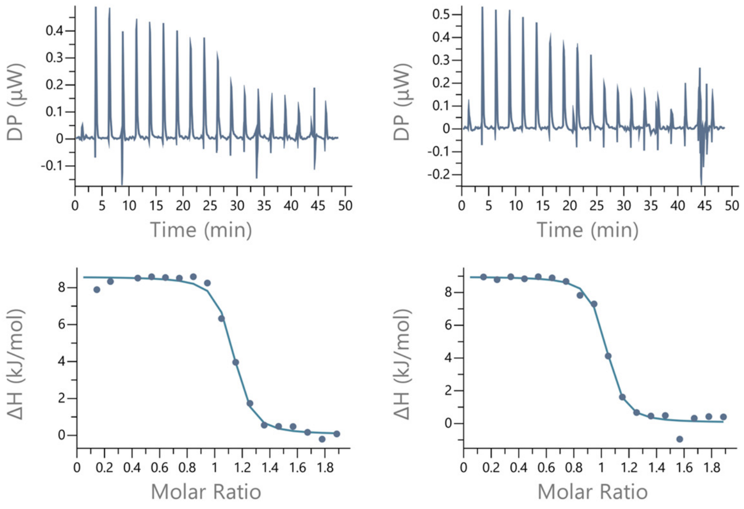

Eu^{3+}			MID1sc9_4xE
	1st Replicate	2nd Replicate	Average
N **(sites)**	0.99 ± 0.01	1.09 ± 0.009	**1.04**
K_D **(Eu^{3+}) [nM]**	92.1 ± 29.3	92.8 ± 25.7	**92.5**
ΔH **(kJ·mol^{-1})**	8.91 ± 0.24	8.54 ± 0.22	**8.73**
ΔG **(kJ·mol^{-1})**	-40.2	-40.2	**-40.2**
-TΔS **(kJ·mol^{-1})**	-49.1	-48.7	**-48.9**

Figure S21 | ITC measurement of Eu^{3+} binding to MID1sc9_4xE. 25 µM MID1sc9_4xE was titrated with freshly prepared 0.5 mM $EuCl_3$ in identical buffer (25 mM HEPES, 100 mM NaCl, pH 7.0), with injection volumes of 1x 0.4 µL and 18x 1 µL. Baseline and off-set of the thermograms were subtracted and differential enthalpy values were fitted. Results are derived from two independent measurements. The determined stoichiometry N, affinity K_D, and thermodynamic values ΔH, ΔG, and -TΔS are shown in the table. The average was calculated from both replicates. Here, the 3rd injection peak of the left thermogram was excluded for the evaluation.

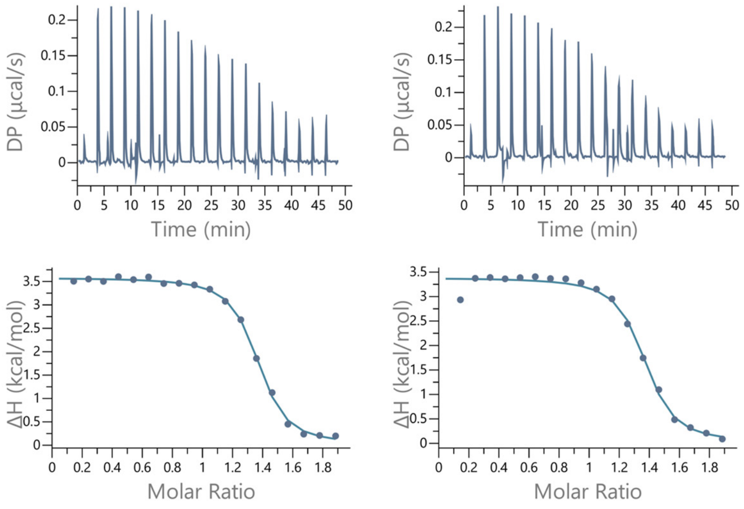

Yb^{3+}			MID1sc9_4xE
	1st Replicate	2nd Replicate	Average
N **(sites)**	1.32 ± 0.01	1.32 ± 0.02	**1.32**
K_D **(Yb^{3+}) [nM]**	167 ± 18.8	179 ± 49.8	**173**
ΔH **(kJ·mol^{-1})**	3.56 ± 0.06	3.36 ± 0.13	**3.46**
ΔG **(kJ·mol^{-1})**	-9.25	-9.20	**-9.23**
-TΔS **(kJ·mol^{-1})**	-12.8	-12.6	**-12.7**

Figure S22 | ITC measurement of Yb^{3+} binding to MID1sc9_4xE. 25 µM MID1sc9_4xE was titrated with freshly prepared 0.5 mM $YbCl_3$ in identical buffer (25 mM HEPES, 100 mM NaCl, pH 7.0), with injection volumes of 1x 0.4 µL and 18x 1 µL. Baseline and off-set of the thermograms were subtracted and differential enthalpy values were fitted. Results are derived from two independent measurements. The determined stoichiometry N, affinity K_D, and thermodynamic values ΔH, ΔG and -TΔS are shown in the table. The average was calculated from both replicates.

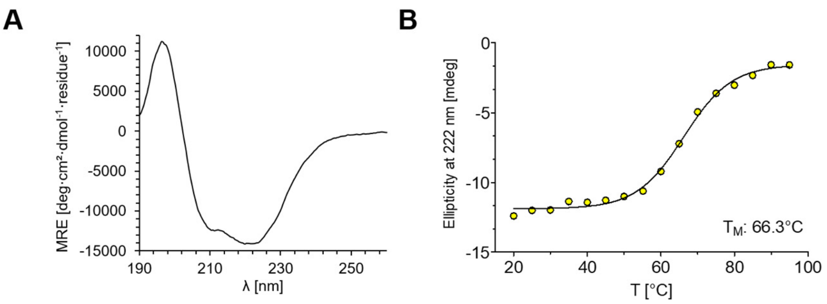

Figure S23 | CD spectroscopy of the original scaffold MID1sc9. (A) CD spectrum at 20°C and (B) melting curve of 10 µM MID1sc9 in 20 mM NH_4HCO_3, pH 8.0. The melting temperature T_M was determined using a sigmoidal Boltzman equation fit.

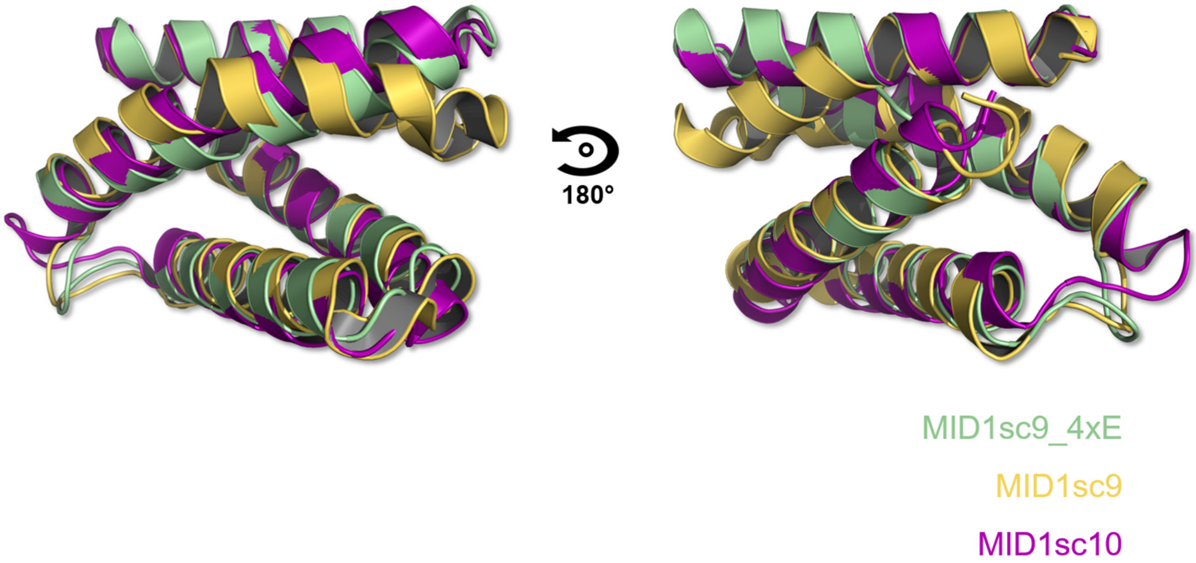

Figure S24 | Structural overlay of MID1 variants. The superimposed crystal structures of MID1sc9 (yellow, PDB ID: 5OD9, Chain A), MID1sc10 (violet, PDB ID: 5OD1) and MID1sc9_4xE (pale green, PDB ID: 9S7R, Chain A) with an RMSD between 1.1 – 1.4 Å.

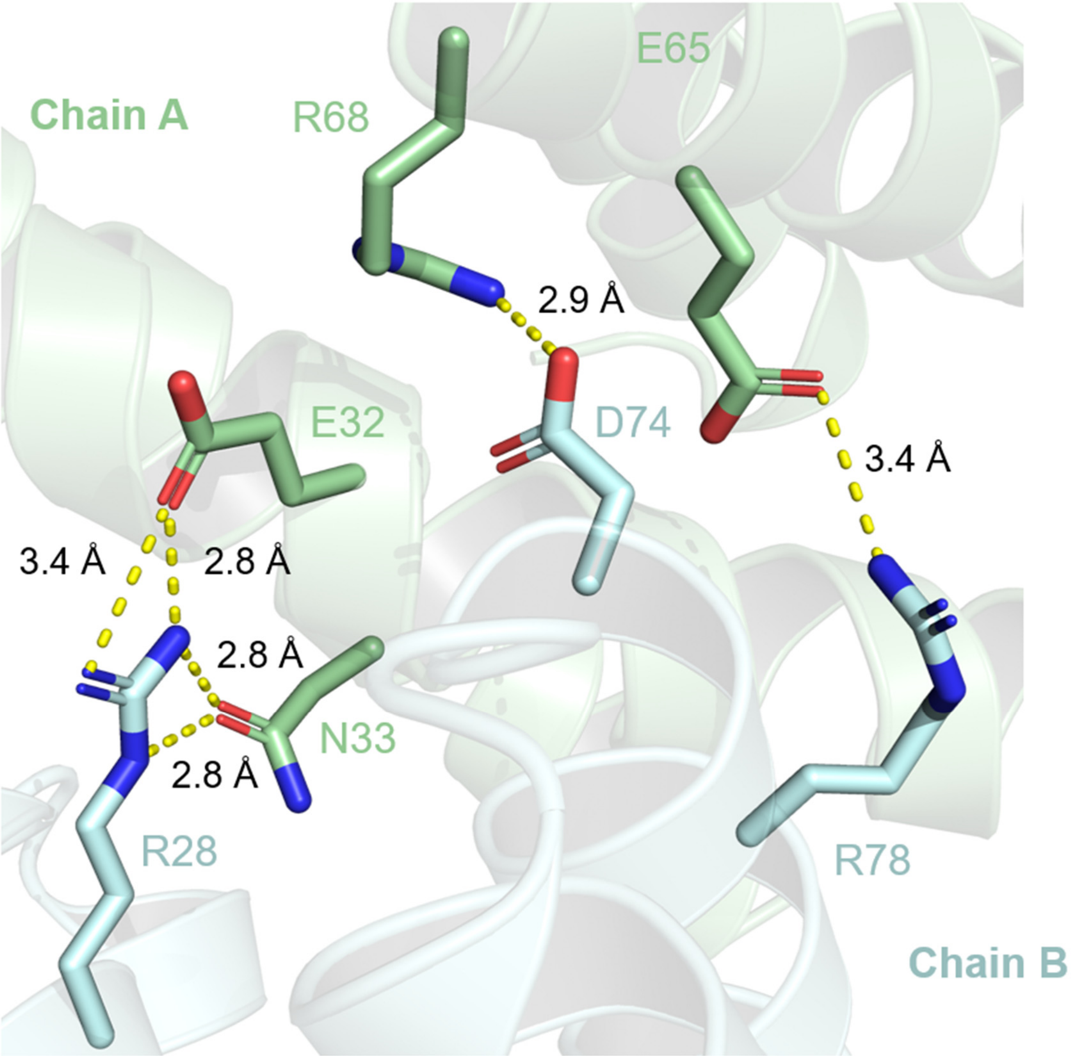

Figure S25| Interactions stabilizing the crystallographic dimer interface in the MID1sc9_4xE structure (PDB ID: 9S7R). Polar interactions between residues at the interface are shown with individual distances.

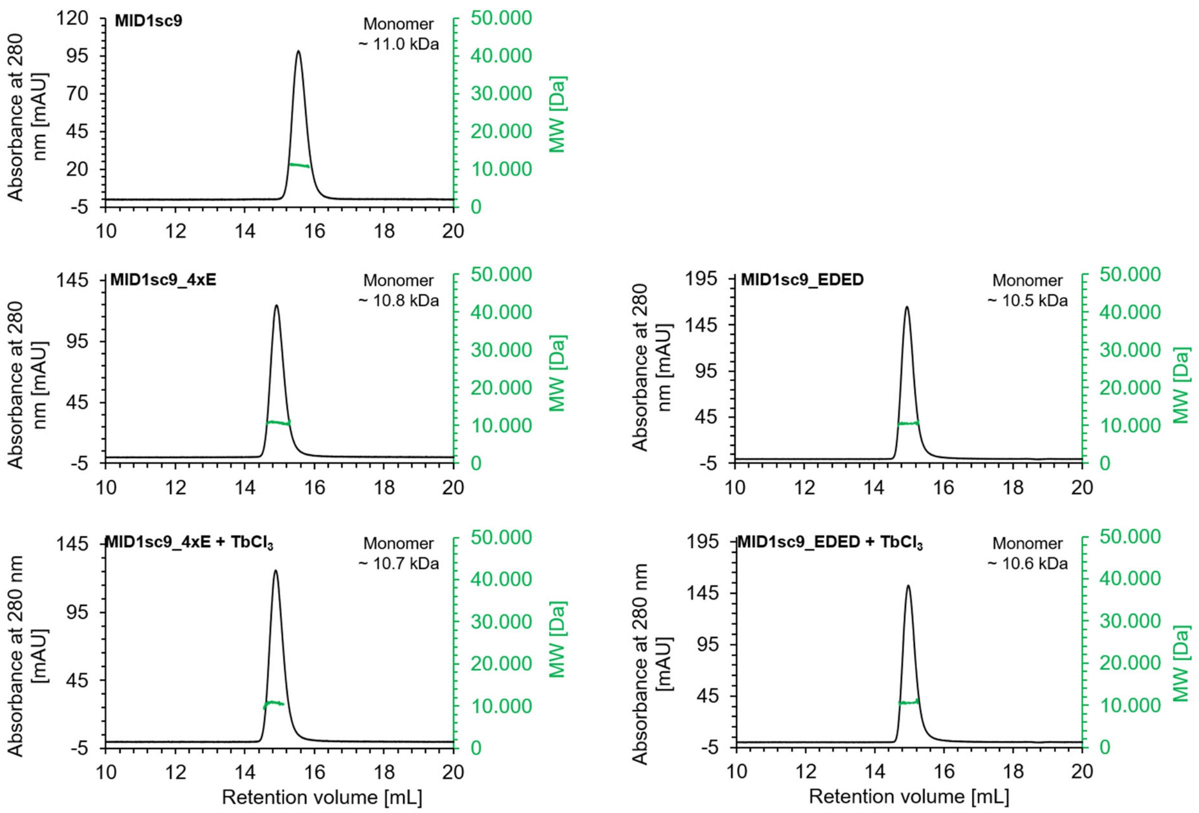

Figure S26 | SEC-SLS analysis of Tb^{3+} treated MID1sc9 proteins. 100 µM protein was prepared in buffer (25 mM HEPES, 100 mM NaCl, pH 7.0) with and without equimolar $TbCl_3$ and incubated for 1 h at rt prior to analysis. The molecular weight of the protein peaks indicates a monomeric state of the identified lanthanide binders MID1sc9_4xE and MID1sc9_EDED in absence and presence of $TbCl_3$. For comparison, the parental variant MID1sc9 was analyzed under the same conditions, showing a slightly shifted peak with monomeric mass.

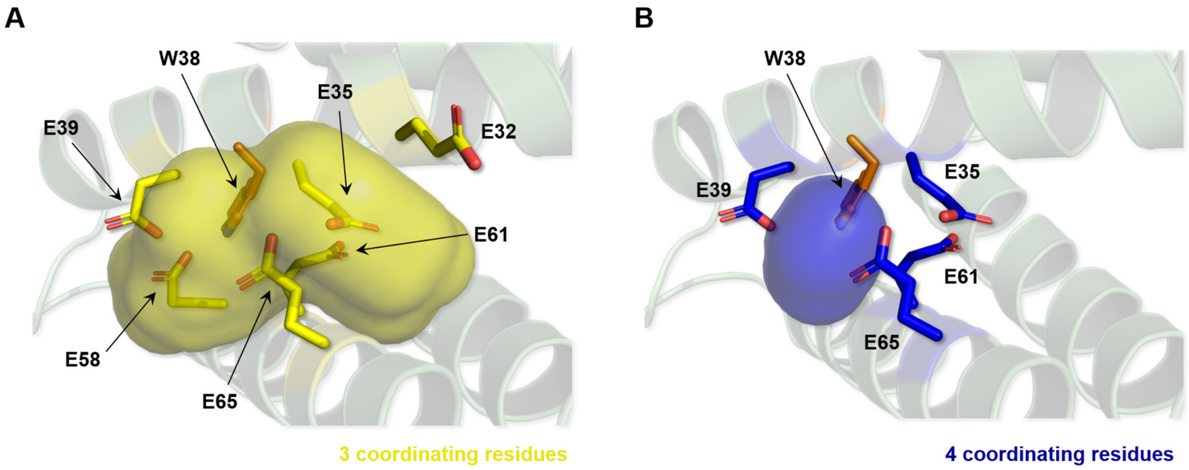

Figure S27 | BioMetAll prediction of putative Ln^{3+} binding sites in MID1sc9_4xE. Colored spheres indicate putative binding sites involving (A) three coordinating residues within 4 Å, and (B) four coordinating residues within 5 Å. The glutamic acid side chains are shown as sticks and highlighted in the respective color. The Trp residue W38, responsible for the sensitized Tb^{3+} luminescence, is shown in orange sticks.

3) Extended Discussion

The role of 2,3-DHN as a competing chelator in solution

2,3-DHN can form complexes with Ln^{3+} ions, as previously reported for related catechols.[11] We determined an apparent dissociation constant $K_{D,app}$ of 130 µM for Tb^{3+} free in solution and a Hill coefficient of 1.66, which hints at cooperative binding with more complex stoichiometry (**Figure S28**) Next, we assessed the effect of 2,3-DHN on the Trp-sensitized Tb^{3+} luminescence signal for the eluted, Tb^{3+} loaded control proteins from our initial assay validation (**Figure S29A**) and the MBP-peptide fusions (**Figure S29B**), respectively. We observed a slight decrease of the Trp-sensitized luminescence after 2,3-DHN was added. The effect was more pronounced for the weaker lanthanide-binding peptides, suggesting that 2,3-DHN can competitively sequester Tb^{3+}. This was also demonstrated for pre-equilibrated equimolar complexes of [PedH-His:Tb^{3+}] and [MID1sc9_4xE:Tb^{3+}] in the presence of excess 2,3-DHN, showing a decreasing Trp-sensitized and increasing in 2,3-DHN-sensitized luminescence signal with higher 2,3-DHN concentration (**Figure S30**).

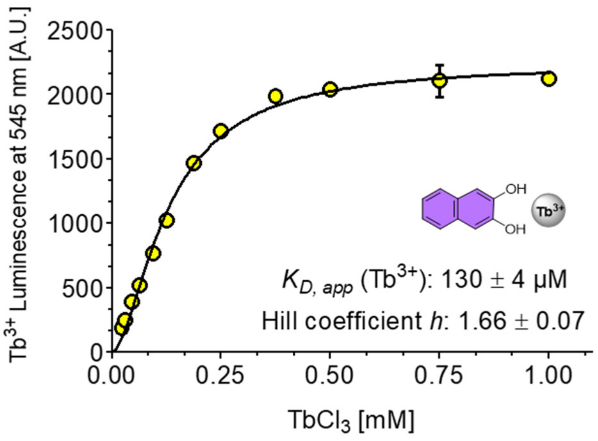

Figure S28 | Apparent affinity of external antenna 2,3-DHN to Tb^{3+}. 10 µM 2,3-DHN was titrated with 23 µM – 1.0 mM $TbCl_3$ in 25 mM HEPES, 100 mM NaCl, pH 7.0. The data was fitted using a Hill equation, assuming cooperative binding. The shown data are averages with standard deviation from a technical triplicate.

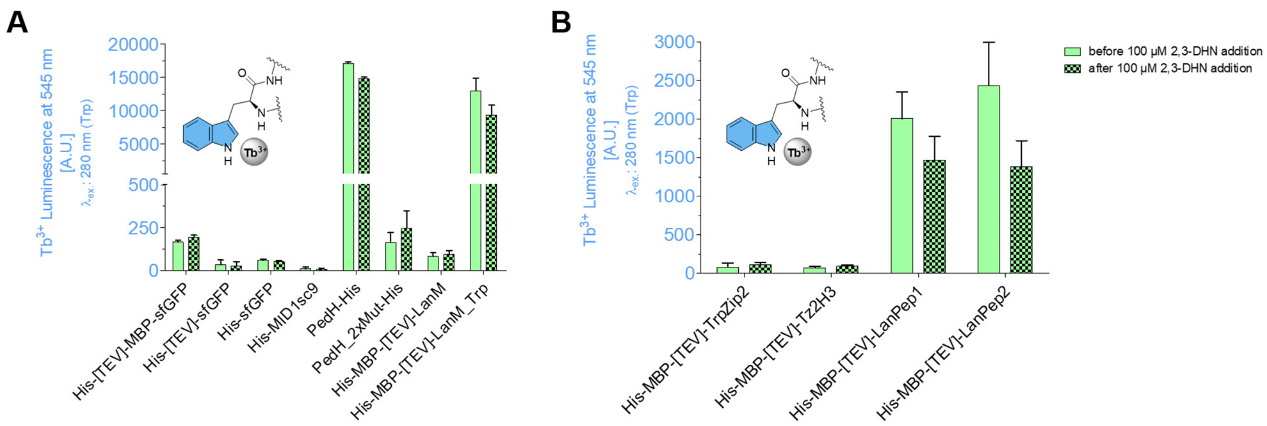

Figure S29 | Effect of 2,3-DHN addition on Trp-sensitized luminescence signals for Tb^{3+} loaded proteins. The sensitized Tb^{3+} luminescence of eluted protein fractions from the initial assay validation (A) and MBP-tagged peptides (B) were recorded before and after addition of 100 μM 2,3-DHN.

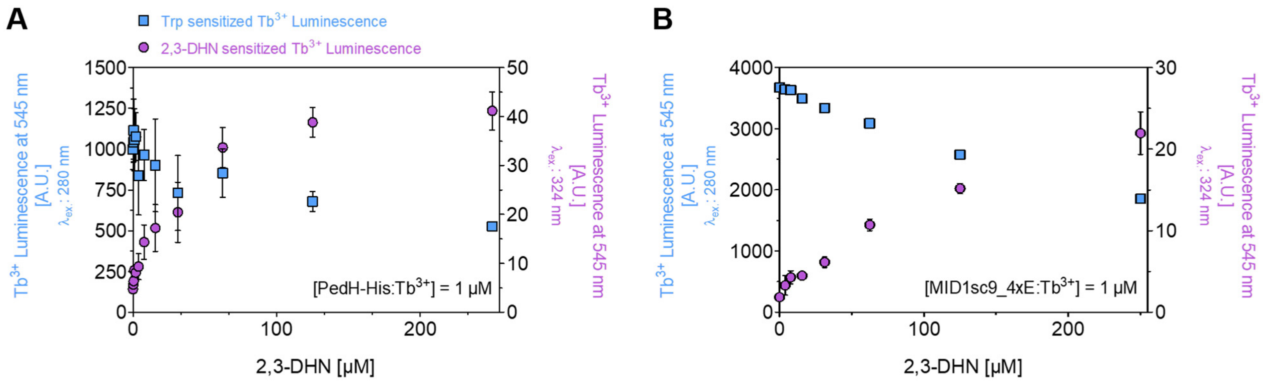

Figure S30 | Tb^{3+} sequestration by excess amounts of 2,3-DHN. 1 μM [PedH-His:Tb^{3+}] complex (A) and 1 μM [MID1sc9_4xE:Tb^{3+}] complex (B) were titrated with 0-250 μM 2,3-DHN and the sensitized Tb^{3+} luminescence signals for Trp (blue squares) and 2,3-DHN (purple circles) were recorded, respectively. The data shown are averages from technical triplicates, with the error bars representing the standard deviation.

4) References

(1) Gibson, D. G. Enzymatic Assembly of Overlapping DNA Fragments. *Methods Enzymol.* **2011**, *498*, 349–361.

(2) Marillonnet, S.; Grützner, R. Synthetic DNA Assembly Using Golden Gate Cloning and the Hierarchical Modular Cloning Pipeline. *Curr. Protoc. Mol. Biol.* **2020**, *130* (1), e115.

(3) Chiu, J.; Tillett, D.; Dawes, I. W.; March, P. E. Site-Directed, Ligase-Independent Mutagenesis (SLIM) for Highly Efficient Mutagenesis of Plasmids Greater than 8kb. *J. Microbiol. Methods* **2008**, *73* (2), 195–198.

(4) Kabsch, W. XDS. *Acta Crystallogr. Sect. D Biol. Crystallogr.* **2010**, *66* (2), 125–132.

(5) Kabsch, W. Automatic Processing of Rotation Diffraction Data from Crystals of Initially Unknown Symmetry Land Cell Constants. *J. Appl. Crystallogr.* **1993**, *26* (pt 6), 795–800.

(6) Rodríguez, D. D.; Grosse, C.; Himmel, S.; González, C.; de Ilarduya, I. M.; Becker, S.; Sheldrick, G. M.; Usón, I. Crystallographic Ab Initio Protein Structure Solution below Atomic Resolution. *Nat. Methods* **2009**, *6* (9), 651–653.

(7) Emsley, P.; Lohkamp, B.; Scott, W. G.; Cowtan, K. Features and Development of Coot. *Acta Crystallogr. Sect. D Biol. Crystallogr.* **2010**, *66* (4), 486–501.

(8) Murshudov, G. N.; Skubák, P.; Lebedev, A. A.; Pannu, N. S.; Steiner, R. A.; Nicholls, R. A.; Winn, M. D.; Long, F.; Vagin, A. A. REFMAC5 for the Refinement of Macromolecular Crystal Structures. *Acta Crystallogr. Sect. D Biol. Crystallogr.* **2011**, *67* (4), 355–367.

(9) Morris, R. J.; Perrakis, A.; Lamzin, V. S. ARP/WARP and Automatic Interpretation of Protein Electron Density Maps. In *Methods in Enzymology*; 2003; Vol. 374, pp 229–244.

(10) Williams, C. J.; Headd, J. J.; Moriarty, N. W.; Prisant, M. G.; Videau, L. L.; Deis, L. N.; Verma, V.; Keedy, D. A.; Hintze, B. J.; Chen, V. B.; Jain, S.; Lewis, S. M.; Arendall, W. B.; Snoeyink, J.; Adams, P. D.; Lovell, S. C.; Richardson, J. S.; Richardson, D. C. MolProbity: More and Better Reference Data for Improved All-Atom Structure Validation. *Protein Sci.* **2018**, *27* (1), 293–315.

(11) Zhu, D. H.; Kappel, M. J.; Raymond, K. N. Coordination Chemistry of Lanthanide Catecholates. *Inorganica Chim. Acta* **1988**, *147* (1), 115–121.
